# Pharmacological Mechanisms Underlying the Therapeutic Effects of Danhong Injection on Cerebral Ischemia

**DOI:** 10.1155/2021/5584809

**Published:** 2021-05-21

**Authors:** Yifei Qi, Yihuai Zou, Lin Chen, Jun Liu, Yingying Zhang, Zhong Wang

**Affiliations:** ^1^Dongzhimen Hospital, Beijing University of Chinese Medicine, Beijing 100700, China; ^2^Institute of Basic Research in Clinical Medicine, China Academy of Chinese Medical Sciences, Beijing 100700, China

## Abstract

**Background:**

Although Danhong injection (DHI) has been proved to be curative, the mechanism of its action against ischemia stroke (IS) is not clear. Here, we explored the therapeutic basis of DHI by network pharmacology.

**Methods:**

Putative targets and activity scores for each compound in DHI were obtained from the Traditional Chinese Medicine System Pharmacology Database, Encyclopedia of Traditional Chinese Medicine, and Quantitative Structure Activity Relationships. Next, target proteins of IS were identified on GeneCards and CTD. Overlapping targets of DHI associated with IS were acquired via Venn diagram. GO and KEGG pathway analyses were done using WebGestalt. Cytoscape software was used for PPI network construction and hub nodes screening. Several validation studies were carried out by using AutoDock-Vina, label-free mass spectrometry, and transcriptome RNA-sequencing.

**Results:**

The 37 active compounds and 66 targets were identified. Of these, 26 compounds and 41 targets belonged to diterpenoid quinones (DQs), which is the predominant category based on chemical structure. The results of enrichments analysis show that 8 DQs target proteins associated with IS were involved in several biological processes and signaling pathway such as apoptotic, cell cycle, cellular response to xenobiotic stimulus process, and the PI3K-Akt signaling. Moreover, 3 nodes in core module involved in PI3K-Akt signaling and 1 hub node were identified by PPI network analysis. Finally, the results of molecular docking and label-free mass spectrometry display good effect on hub node regulation in DHI treatment.

**Conclusions:**

DQs is the predominant category of DHI and play an important role in antiapoptotic activity mediated by modulating PI3K-Akt signaling. Our findings offer insight into future research and clinical applications in IS therapy.

## 1. Introduction

Cerebral stroke is the second most leading cause of death and the main cause of disability in worldwide. According to World Health Organization, it led to 6 million deaths in 2016 [1,2]. Ischemic stroke (IS) accounts for nearly 80% of cases and is characterized by occlusion of the cerebral artery, which leads to a temporary lack of glucose and oxygen supply in brain [3,4]. Standard IS therapies involve intravenous injection of recombinant tissue plasminogen activator (t-TPA), antiplatelet therapy, and anticoagulants for patients with atrial fibrillation, or interventions to limit cell damage [5–7]. These single target therapies are limited by the narrow time window of thrombolysis, hemorrhagic tendency, and high cost [8]. For this reason, novel therapeutic strategies are needed.

Danhong injection (DHI) is the most popular Chinese medicine for the treatment of IS and promotes blood circulation and resolves stasis to promote regeneration [9]. DHI is extracted from Radix *Salvia miltiorrhizae* (Danshen, DS) and Flos Carthami Tinctorii (Honghua, HH) [10]. Clinical studies show that DHI is efficacious and safe for IS management [11]. Pharmacological studies show that DHI is neuroprotective in rat models of cerebral ischemic reperfusion injury, possibly by enhancing angiogenesis [12], ameliorating blood-brain barrier (BBB) disruption, relieving brain swelling and hemorrhage [13], attenuating astrocytic dysfunction [14], and reversing neutrophil infiltration [15]. Although multiple studies have investigated the mechanisms underlying DHI action, its underlying pharmacological mechanisms have not been elucidated at the systematic level.

Network pharmacology characterized by systematization and wholeness and has potential to uncover TCM mechanism via biological networks construction. Chinese herbal medicines act on multitargets via multiple channels, which is very similar to the multipathways and multilevel features of network pharmacology [16]. Here, we used various publicly available bioinformatics resources to investigate the potential pharmacological mechanisms of DHI in IS treatment.

## 2. Materials and Methods

### 2.1. Screening for Active DHI Compounds

DS and HH chemical compound data were collected from the Traditional Chinese Medicine Systems Pharmacology (TCMSP, https://tcmspw.com/index.php) database and analysis platform and the Encyclopedia of Traditional Chinese Medicine (ETCM, http://www.tcmip.cn/ETCM/index.php/Home/) database [17,18]. Next, pharmacokinetic properties and comprehensive drug-likeness grading of candidate compounds in DHI were filtered. Frist, the effective compounds screening performed using oral bioavailability (OB) ≥30%, drug-likeness (DL) index ≥0.18, and BBB ≥ −0.3. Next, the second screening was performed using Lipinski Rule of Five. Two-dimensional (2D) structure and canonical smiles of the active compounds were demonstrated using PubChem.

### 2.2. Identification of Ischemic Stroke Targets and Collection of Putative Target Proteins

IS-associated targets were identified based on comparative toxicogenomics database (CTD, http://ctdbase.org/) [19] and the GeneCards (http://www.genecards.org/) [20], using scores >50% and 30% as cutoffs for higher correlation with IS, respectively. Prediction of proteins related to DHI active compounds was done using quantitative structure activity relationships: TargetNet (QSAR-TargetNet, http://targetnet.scbdd.com) [21] and identified targets transformed to gene symbols on R. The overlapping targets between IS-related targets and active compounds were retained for Venn analysis (https://bioinfogp.cnb.csic.es/tools/venny/index.html).

### 2.3. GO and KEGG Enrichment and Network Construction

Go and KEGG enrichment analysis was performed using WEB-based Gene Set Analysis Toolkit (WebGestalk, http://www.webgestalt.org/) [22,23]. The initial parameter setting is shown as follows: species parameter set to “Homo sapiens,” filter values parameter set to 0.05, and the term with fewer than 3 detected genes were filtered out. Based on the results of enrichment analysis, the TCM compound regulatory network and the compound-targets-pathway regulatory network were visualized on Cytoscape 3.7.2 software [24].

### 2.4. PPI Network Construction and Topological Analysis

Overlapping protein targets of DQs associated with IS were considered as initial nodes. Next, the “input nodes and its neighbors” method was used to construct the PPI network using the “bisoGenet” plugin on Cytoscape. The species parameter was set to “Homo sapiens.” “Database of Interacting Proteins,” “Human Protein Reference Database,” “Biological General Repository for Interaction Datasets,” “Molecular INTeraction Database,” “IntAct molecular interaction database,” and “Biomolecular Interaction Network Database” were the main resources for PPI network construction. To extract the core module, we used a method combining degree centrality (DC) and betweenness centrality (BC) values, which was effective in identifying key proteins [25]. DC and BC reflect the influence of corresponding nodes in the full network. The higher the DC and BC, the more significant the node.

### 2.5. Molecular Docking

3D structures of key active compounds were obtained from PubChem (https://pubchem.ncbi.nlm.nih.gov) and saved in SDF format and subsequently converted to PDB format using OpenBabal 2.3.0. Crystal structures of key receptors were downloaded from protein data bank (PDB, http://http://www.pdb.org) and processed by removing ligand and water motifs and adding hydrogen using the Discovery Studio software. Rotatable bonds were then set in the flexible residues and converted to PDBQT type and resulting grid center site information analyzed using AutoDockTools-1.5.6. Moreover, AutoDock-Vina software was used to calculate binding affinity and find probable binding sites.

### 2.6. Label-Free Mass Spectrometry Analyses

For evaluating the protein expression changes after DHI treatment in patients with acute ischemic stroke, a total of 6 acute ischemic stroke patients and 6 healthy volunteers without diseases including cerebral injury were enrolled from General Hospital of Northern Theater Command (Trial registration: ClinicalTrials identifier: NCT02176395). The peripheral venous blood samples of healthy volunteers, patients before treatment, and patients after day 14 of DHI treatment were obtained. Firstly, the albumin was removed from the serum using the Abundant Protein Depletion Spin Columns kit (ThermoScientific). Next, the label-free mass spectrometry analyses were carried out on the nLC-Easy1000-Orbitrap Fusion (ThermoScientific). Thirdly, protein identification was performed in the NCBI Database using Mascot2.3. Statistical differences between groups were analyzed using Kruskal–Wallis test for nonparametric values with SPSS23.0.

### 2.7. GEO Database Validation of DHI Treating IS

To further validate the effects of DHI for the treatment of IS, the genetic samples (series: GSE106680) were obtained from GEO databases (https://www.ncbi.nlm.nih.gov/geo/). In this study, Sprague Dawley (SD) rats were divided into 3 experimental groups as follows: sham group, vehicle group, and DHI group, each group with 3 samples. To study on protective effects of cerebral ischemia/reperfusion-induced damage, the expression changes of the key receptors after 14-day treatment in cerebral ischemia/reperfusion model were compared among the 3 groups [14]. Statistical differences between groups were analyzed using ANOVA with SPSS23.0.

## 3. Results

### 3.1. Identification of Active Compounds and Putative Target Proteins

**Figure 1:**
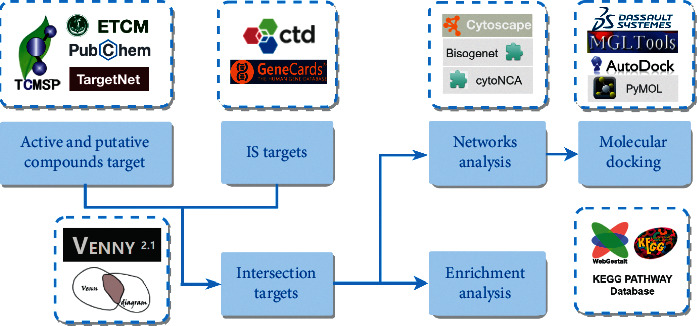
Network pharmacology approach for deciphering pharmacological mechanisms of DHI activity in cerebral ischemia.

**Figure 2:**
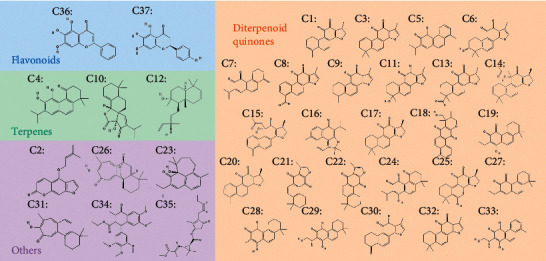
2-dimensional (2D) molecular structures and classification of 37 DHI candidate compounds. There are 4 classifications, including diterpenoid quinones (26), terpenes (3), flavonoids (2), and others (6).

Details on investigating the pharmacological mechanisms of DHI against IS are shown in Figure 1. DHI consists of Danshen and Honghua. 45 DS and 12 HH components were obtained from the TCMSP database. After screening by pharmacokinetic properties and the Lipinski rule of 5, duplicate removal, and verification on PubChem, 37 active compounds were obtained and the divided into 4 categories: 26 diterpenoid quinones (DQs) (e.g., dehydrotanshinone II A, Danshenol A, and tanshinaldehyde, among others), 3 terpenes (e.g., arucadiol, przewalskin B, and sclareol), 2 flavonoids (e.g., baicalein and carthamidin), and 6 others (e.g., isoimperatorin and Microstegiol) (Figure 2). Based on ETCM database analysis, 21 compounds exhibited a good grade using the ADMET criterion (Table S1).

Higher combining probability indicated a close integration between compounds and targets. A total of 371 putative targets were identified by combined probability score along with the 37 candidate compounds using QSAR-TargetNet. After duplicate removal and name conversion, 66 targets were obtained, 41 of which were putative target proteins of DQs.

**Figure 3:**
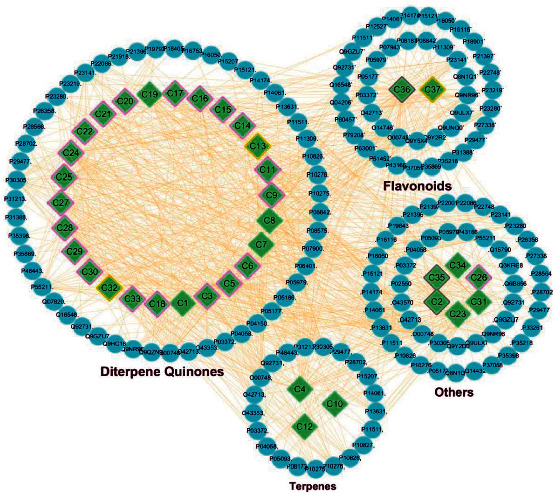
Construction of a compound-target regulatory network and the classification of putative target proteins of DHI compounds. The candidate compounds (diamond mesh node with green) are divided into groups by structural category. The candidate compounds are divided into good (purple border), moderate (yellow border), week (gray border), and N/A (no border). Similarly, the putative target proteins (the ellipse mesh node with blue) are grouped and surrounded with corresponding compounds.

Based on this, a compound-target (CT) network was constructed.  Figure 3 shows putative targets surrounded by various categories of grouped compounds. Candidate compounds are divided into good, moderate, week, and N/A, marked by purple, yellow, gray, and white borders, respectively. Of these, 20 compounds with good grade belong to DQs, 95% of total. We found that DQs have good pharmacokinetic properties based on the ADMET criterion, and that they may be the main active compounds driving DHI neuroprotective effects (Table S2).

### 3.2. Potential DHI Targets in Ischemic Stroke Treatment

**Figure 4:**
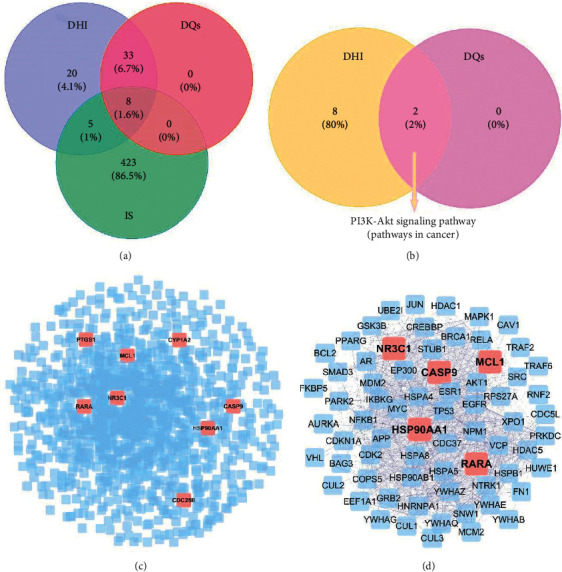
Topological analysis of the protein-protein interaction network. (a) Overlapping target proteins Venn diagram between DHI targets, DQs targets, and IS targets. (b) Overlapping KEGG signaling Venn diagram between DHI and DQs. (c) PPI network constructs based on 8 overlapping targets of DQs associated with IS. Herein, 1162 protein nodes were obtained. After extracted by top 20^th^ percentile of DC and top 6^th^ percentile BC, a total of 70 nodes were obtained. (d) Core module identified by PPI network analysis. 5 overlapping protein associated with PI3L/Akt signaling are marketed as red squares.

CTD and GeneCards analyses identified 107872 and 3443 IS-associated gene entries, respectively. With prioritized inference and relevance scores, 436 gene entries were identified from the 2 databases and merged. These targets served as key putative IS-associated proteins (Tables S3-S4). Of the predicted DHI targets, 13 target proteins associated with IS were found, including caspase-9, MAOB, MAOA, NR3C1, CDC25B, RARA, CYP1A2, MCL-1, HSP90AA1, PTGS1, CYP2C19, ABCB1, and RELA. Of these, 8 belonged to the DQs (Figure 4(a)). The putative proteins of DQs' targets associated with IS can uncover the potential functions of DHI treating IS.

### 3.3. Core Module and Hub Nodes in PPI Network

We obtained a PPI network comprising 1162 nodes and 19211 edges, based on 8 overlapping target proteins belonging to DQs (see Figure 4(c) and 4(d)). Next, a subnetwork comprising 233 nodes and 6148 edges was extracted by top 20th percentile of DC from the PPI network, and then a core module comprising 70 proteins was re-extracted by top 6th percentile of BC from the subnetwork above. We found that 5 of 8 overlapping protein targets of DQs, MCL-1, HSP90AA1, NR3C1, CASP9, and RARA were always in the subnetwork and the core module. Notably, HSP90AA1 was the hub node with highest DC and BC (Tables S5-S6), and 3 of those 5 overlapping protein targets were involved in phosphatidylinositol-3 kinase (PI3K)/protein kinase B (PKB/Akt) signaling.

### 3.4. Biological Function of DQs Targeting on IS

**Figure 5:**
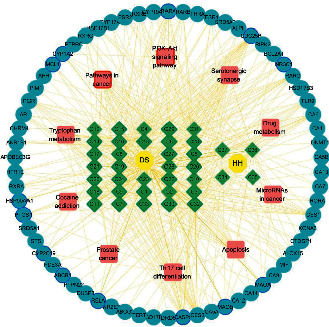
Construction of compound-target-pathway network. Yellow diamond represents herbs in DHI; green diamond represents active compounds, with green border representing good pharmacokinetic properties; red rectangle represents KEGG signaling enriched form 13 overlapping target proteins of DHI associated with IS, and the KEGG signaling enriched from 8 overlapping targets of the DQs associated with IS was annotated with red border; blue ellipse nodes indicate putative targets of DHI, and targets of DQs annotated with blue border.

To provide further insight into the mechanisms underlying DQs effects on IS at the systematic level, GO and KEGG pathway analyses were done and gene functions depicted based on effect on biological process (BP), cellular component (CC), and molecular function (MF). There were 50 GO terms and 2 KEGG pathways enriched from the 8 overlapping target proteins (Tables S7-S8). The GO-BPs mainly involve in oxygen-containing compound, organic cyclic compound, and apoptotic process, and the 2 KEGG pathways were PI3K-Akt signaling pathway and pathways in cancer. Notably, these two KEGG pathways were the common pathways enriched from the DQs target proteins and DHI target proteins (Figure 4(b) and Table S9). According to KEGG enrichment analysis and PPI network analysis, the PI3K-Akt signaling pathway may be the most important signaling related to the treatment of DHI for IS. Based on these, a compound-targets-pathway (CTP) network was constructed (Figure 5).

### 3.5. The Affinity between Compounds and Receptors

**Figure 6:**
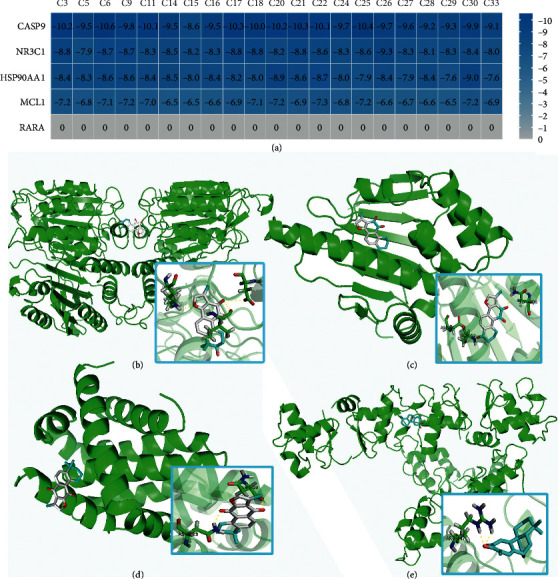
Results of molecular docking and docking simulation. (a) Heat map of the binding energies. (b) 3alpha-hydroxytanshinone IIa (C6) in the protein caspase-9 (PDB ID:2AR9). (c) Nortanshinone (C30) with HSP90AA1 (2BTH). (d) Isotanshinone IIa (C22) with MCL-1 (3MK8). (e) Miltipolone (C26) with NR3C1 (6CFN).

Binding energy can be calculated to predict affinity between 2 counterparts. Twenty-one active DHI compounds with good pharmacokinetic properties were molecularly docked with 5 key receptors including MCL-1 (PDB ID: 3MK8), HSP90AA1 (2BTH), caspase-9 (2AR9), RARA (5K13), and NR3C1 (6CFN). Binding energy less than 0 indicated that spontaneous combination occurred between 2 molecules. The lower the binding energy is, the stronger the affinity between compounds and targets is. A total of 105 ligand-receptor combinations were computed. Except RARA, most DHI components bind well with key receptors and 93 combinations had affinities of < –7 kcal/mol, accounting for 88.6%, with the strongest binding being with Casp9 (–10.6 kcal/mol) (Figure 6(a)). Molecular docking structures are detailed in Figure 6(b)–6(e).

### 3.6. Validation on the Targets of DHI for the Treatment of IS

**Figure 7:**
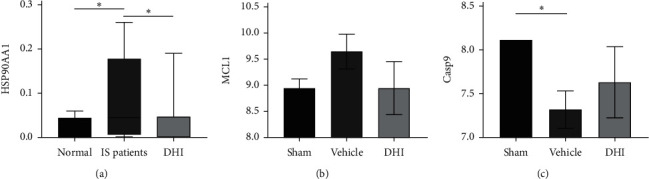
The expression results of 3 nodes in core module associated with PI3K/Akt signaling. (a) The mass spectrometric quantification result of HSP90AA1. (b, c) The MCL-1 and Casp9 mRNA expression levels from mRNA-sequencing results, respectively (^*∗*^*P* < 0.05).

According to the label-free mass spectrometry analyses from our previous trial, compared with healthy volunteers, the expression of HSP90AA1 was upregulated after IS (*P* < 0.05), and after treated with DHI for 14 days, the HSP90AA1 expression was downregulated (*P* < 0.05) (Figure 7(a)). Moreover, according to the expression of mRNA from another independent experiment, compared with the sham group, the expression of Casp9 was downregulated (*P* < 0.05), while MCL-1 showed an upregulation trend in the vehicle. After 14-day treatment of DHI, the expression of Casp9 was upregulated and MCL-1 was downregulated, although there was no significantly statistical difference compared with the vehicle (Figures 7(b) and 7(c)).

## 4. Discussion

Based on chemical structure analysis and network pharmacology, we find DQs are the major category in DHI compounds. Biological processes of DQs protein associated with IS were involved in regulation of apoptotic, positive regulation of molecular function, and regulation of cell proliferation process, and PI3K-Akt signaling pathway may closely involve in mechanism of action of DQs proteins associated with IS. This find is consistent with the previous studies [26, 27], which confirmed the neuroprotective effect of DHI via the PI3K-Akt pathway, since the specific inhibitor of PI3K-Akt pathway could weaken the neuroprotective effect on brain damage due to ischemic reperfusion in the rats with the middle cerebral artery occlusion.

To make further investigation, we find core module and hub node in PPI network by using DC and BC values in our work. Casp9, Hsp90AA1, and MCL1 were identified from the core module involved in PI3K-Akt signaling. HSP90AA1 was the hub node since highest DC and BC. To verify this, molecular docking simulation was used to predict the binding interaction of compounds in reporter's binding pocket. The results shown that most compounds docked well with all three targets. PKB/Akt can mediate resistance to hypoxia-ischemia through survival and inactivation of apoptosis-associated proteins [28]. HSP90AA1 as the hub node of DHI for the treatment of IS, which could protect Akt kinase activity from dephosphorylation [29]. The active Akt subsequently regulate various targets, including caspase-9 and myeloid cell leukemia-1 (MCL-1) [30–33]. MCL-1 involved in proapoptotic function and led to the activation of the downstream caspase cascade [34–36]. Finally, our label-free mass spectrometry results show that Hsp90aa1 may be modulating apoptosis via modulation of PI3K/Akt signaling.

**Figure 8:**
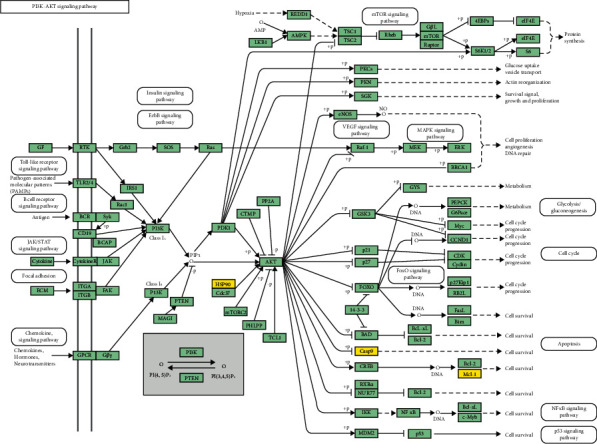
Modulating PI3K-Akt signaling pathway. Key receptors are shown in yellow, and other protein targets are in green (picture derived from KEGG database).

Therefore, our results indicated that DHI and its major category DQs effectively exert antiapoptosis functions by regulating HSP90AA1-induced PI3K/Akt signaling and other downstream molecules like MCL-1 and caspase-9 (Figure 8). In summary, our findings offer new insights on future DHI research and its applications in IS treatment. However, some shortcomings of our study should be considered. Apoptosis is a complicated process regulated by multitargets. Although our results were verified using label-free mass spectrometry and transcriptome RNA-sequencing, the experimental work with large sample size and multiple-time-point will be done in future study to find more evidence.

## 5. Conclusion

Here, we used network pharmacology to investigate the potential mechanisms underlying DHI effects on IS. Our data show that DHI is antiapoptotic via multifaceted activity. DHI, especially the main category (diterpenoid quinones), appears to promote cell survival effects via PI3K-Akt signaling. It targets on both upstream and downstream of the PI3K-Akt signaling, which may be its main mechanism against IS. These results offer rationale for future DHI research and applications in treating IS.

## Data Availability

All data used to support the findings of this study are included within the figure and supplementary tables.

## Abbreviations

IS: Ischemic stroke
r-TPA: Recombinant tissue plasminogen activator
DHI: Danhong injection
DS: Danshen
HH: Honghua
BBB: Blood-brain barrier
TCMSP: Traditional Chinese Medicine Systems Pharmacology database
ETCM: Encyclopedia of Traditional Chinese Medicine
OB: Oral bioavailability
DL: Drug-likeness
2D: Two-dimensional
CTD: Comparative Toxicogenomics database
QSAR-TargetNet: Quantitative structure activity relationships-TargetNet
WebGestalk: WEB-based gene set analysis toolkit
DQs: Diterpenoid quinones
GO: Gene Ontology
KEGG: Kyoto Encyclopedia of Genes and Genomes
3D: Three-dimensional
PDB: Protein data bank
CT: Compound-targets
BP: Biological process
CC: Cellular component
MF: Molecular function
CTP: Compound-targets-pathway
DC: Degree centrality
BC: Betweenness centrality
PI3K/Akt: Phosphatidylinositol-3 kinase/protein kinase B
HSP90: Heatshock protein 90
MCL-1: Promotes myeloid cell leukemia-1
NF-*κ*B: Nuclear factor kappa-B.

## Supplementary Materials

Supplementary Materials Table S1: the 37 candidate compounds of Danhong injection. Table S2: the 371 putative target proteins for the compounds. Table S3: the 413 IS-associated *Homo sapiens* target proteins from CTD with an inference score of ≥50. Table S4: the 61 IS-associated target proteins of *Homo sapiens* from Genecards with an inference score of ≥30. Table S5: degree centrality of nodes in PPI network. Table S6: betweenness centrality of nodes in the PPI network. Table S7: the GO functional enrichment analysis of diterpenoid quinones. Table S8: the KEGG pathway enrichment of diterpenoid quinones. Table S9: the KEGG pathway enrichment of DHI compounds.
