# Supplementary material for: Pharmacological Mechanisms Underlying the Therapeutic Effects of Danhong Injection on Cerebral Ischemia
Source: Evid Based Complement Alternat Med. 2021 May 21;2021:5584809. doi: 10.1155/2021/5584809 (PMC8163534; doi:10.1155/2021/5584809)
Supplement: Supplementary Materials — Table S1: the 37 candidate compounds of Danhong injection. Table S2: the 371 putative target proteins for the compounds. Table S3: the 413 IS-associated Homo sapiens target proteins from CTD with an inference score of ≥50. Table S4: the 61 IS-associated target proteins of Homo sapiens from Genecards with an inference score of ≥30. Table S5: degree centrality of nodes in PPI network. Table S6: betweenness centrality of nodes in the PPI network. Table S7: the GO functional enrichment analysis of diterpenoid quinones. Table S8: the KEGG pathway enrichment of diterpenoid quinones. Table S9: the KEGG pathway enrichment of DHI compounds. [file 5584809.f1.zip › 5584809.f1/S1 (2).pdf]

Table S1. The 37 candidate compounds of Danhong injection

MW: Molecular Weight; OB: Oral Bioavailability; Caco-2: Caco-2 permeability; DL: Drug-likeness; BBB: Blood-brain Barrier; HL: Drug half-life; MF: Molecular Formula;
Lipinski Rule of Five: the number presented the percentage of the entries that meet the criteria.
ADMET: Absorption,Distribution, Metabolism, Excretion and Toxicity evaluated by ETCM, QED ＞0.67 (Good), QED: 0.67-0.49 (Moderate), QED: ＜0.49 (Weak).

Ingredients	Code	Compound Name	Mol ID	MW	OB (%)	Caco-2	BBB	DL	HL	MF	PubChem CID	Drug-likeness Weight	ADMET	CAS	Lipinski Rule of Five	Classification	Canonical SMILES
DanShen	C1	1,2,5,6-tetrahydrotanshinone	MOL001601	280.34	38.75	0.96	0.39	0.36	18.05	C18H16O3	124416	NA	NA	126979-84-8	1	Diterpenoid Quinones	CC1COC2=C1C(=O)C(=O)C3=C2C=CC4=C3CCC=C4C
DanShen	C2	Isoimperatorin	MOL001942	270.3	45.46	0.97	0.66	0.23	-1.44	C16H14O4	68081	0.486	Weak	482-45-1	1	Others	CC(=CCOC1=C2C=CC(=O)OC2=CC3=C1C=CO3)C
DanShen	C3	Dehydrotanshinone II A	MOL002651	292.35	43.76	1.02	0.52	0.4	23.71	C19H16O3	128994	0.683	Good	119963-50-7	1	Diterpenoid Quinones	CC1=COC2=C1C(=O)C(=O)C3=C2C=CC4=C3C=CCC4(C)C
DanShen	C4	Arucadiol	MOL007036	298.41	33.77	1.19	0.8	0.29	14.91	C19H22O3	11011966	NA	NA	142546-15-4	1	Terpenes	CC(C)C1=C(C(=C2C(=C1)C=CC3=C2C(=O)CCC3(C)C)O)O
DanShen	C5	3,4-Phenanthrenedione, 8-methyl-2-(1-methylethyl)-	MOL007041	264.34	40.86	1.23	0.81	0.23	14.89	C18H16O2	135872	0.72	Good	87112-49-0	1	Diterpenoid Quinones	CC1=C2C=CC3=C(C2=CC=C1)C(=O)C(=O)C(=C3)C(C)C
DanShen	C6	3alpha-Hydroxytanshinone IIA	MOL007045	310.37	44.93	0.53	0.22	0.44	23.78	C19H18O4	14610644	0.757	Good	97399-71-8	1	Diterpenoid Quinones	CC1=COC2=C1C(=O)C(=O)C3=C2C=CC4=C3CCC(C4(C)C)O
DanShen	C7	4-Methylenemiltirone	MOL007049	266.36	34.35	1.25	0.87	0.23	14.6	C18H18O2	14609851	NA	NA	NA	1	Diterpenoid Quinones	CC(C)C1=CC2=C(C3=C(C=C2)C(=C)CCC3)C(=O)C1=O
DanShen	C8	formyltanshinone	MOL007058	290.28	73.44	0.54	-0.28	0.42	24.12	C18H10O4	14609847	NA	NA	NA	1	Diterpenoid Quinones	CC1=COC2=C1C(=O)C(=O)C3=C2C=CC4=C(C=CC=C43)C=O
DanShen	C9	Methylenetanshinquinone	MOL007061	278.32	37.07	1.03	0.46	0.36	24.33	C18H14O3	105118	0.676	Good	67656-29-5	1	Diterpenoid Quinones	CC1=COC2=C1C(=O)C(=O)C3=C2C=CC4=C3CCCC4=C
DanShen	C10	Przewalskin B	MOL007064	330.46	110.32	0.34	0.22	0.44	2.17	C20H26O4	16102114	NA	NA	NA	1	Terpenes	CC(C)C1=CC23CCC4C(=CCCC4(C)C)C2OC(=O)C3(C1=O)O
DanShen	C11	przewaquinone c	MOL007069	296.34	55.74	0.42	-0.3	0.4	23.7	C18H16O4	126071	0.745	Good	96839-29-1	1	Diterpenoid Quinones	CC1=COC2=C1C(=O)C(=O)C3=C2C=CC4=C3CCCC4(C)O
DanShen	C12	Sclareol	MOL007077	308.56	43.67	0.84	0.51	0.21	4.71	C20H36O2	163263	NA	NA	515-03-7	1	Terpenes	CC1(CCCC2(C1CCC(C2CCC(C)(C=C)O)(C)O)C)C
DanShen	C13	Tanshinaldehyde	MOL007079	308.35	52.47	0.57	-0.07	0.45	23.49	C19H18O4	124268	0.591	Moderate	142694-58-4	1	Diterpenoid Quinones	CC1COC2=C1C(=O)C(=O)C3=C2C=CC4=C3CCCC4(C)C=O
DanShen	C14	Danshenol B	MOL007081	354.48	57.95	0.53	0.11	0.56	4.28	C22H26O4	3083515	0.887	Good	189308-09-6	1	Diterpenoid Quinones	CC1COC2=C1C(=O)C(C3=C2C=CC4=C3CCCC4(C)C)(CC(=O)C)O
DanShen	C15	Danshenol A	MOL007082	336.41	56.97	0.33	-0.01	0.52	5.15	C21H20O4	3083514	0.915	Good	189308-08-5	1	Diterpenoid Quinones	CC1COC2=C1C(=O)C(C3=C2C=CC4=C(C=CC=C43)C)(CC(=O)C)O
DanShen	C16	Salvilenone	MOL007085	292.4	30.38	1.46	1.07	0.38	20.81	C20H20O2	389885	0.776	Good	57517-08-5	1	Diterpenoid Quinones	CC1=C2C=CC3=C4C2=C(C=C1)C(=O)C(=C4OC3(C)C)C(C)C
DanShen	C17	Cryptotanshinone	MOL007088	296.39	52.34	0.95	0.51	0.4	17.3	C19H20O3	160254	0.685	Good	35825-57-1	1	Diterpenoid Quinones	CC1COC2=C1C(=O)C(=O)C3=C2C=CC4=C3CCCC4(C)C
DanShen	C18	Danshenxinkun D	MOL007093	336.41	38.88	0.67	-0.15	0.55	30	C21H20O4	127172	0.869	Good	98873-76-8	1	Diterpenoid Quinones	CC1COC2=C(C1(C)CO)C(=O)C3=C(C2=O)C4=CC=CC(=C4C=C3)C
DanShen	C19	Deoxyneocryptotanshinone	MOL007098	298.41	49.4	0.85	0.24	0.29	27.17	C19H22O3	15690458	NA	NA	27468-20-8	1	Diterpenoid Quinones	CC(C)C1=C(C2=C(C3=C(C=C2)C(CCC3)(C)C)C(=O)C1=O)O
DanShen	C20	Dihydrotanshinone I	MOL007101	278.32	45.04	0.95	0.43	0.36	18.32	C18H14O3	11425923	0.695	Good	87205-99-0	1	Diterpenoid Quinones	CC1COC2=C1C(=O)C(=O)C3=C2C=CC4=C(C=CC=C43)C
DanShen	C21	Isocryptotanshinone	MOL007108	296.39	54.98	0.93	0.34	0.39	31.92	C19H20O3	626608	0.732	Good	22550-15-8	1	Diterpenoid Quinones	CC1COC2=C1C(=O)C3=C(C2=O)C4=C(C=C3)C(CCC4)(C)C
DanShen	C22	Isotanshinone IIA	MOL007111	294.37	49.92	1.03	0.45	0.4	24.73	C19H18O3	626354	0.709	Good	20958-15-0	1	Diterpenoid Quinones	CC1=COC2=C1C(=O)C3=C(C2=O)C4=C(C=C3)C(CCC4)(C)C
DanShen	C23	Microstegiol	MOL007118	298.46	39.61	1.05	0.99	0.28	4.52	C20H26O2	403772	NA	NA	143246-41-7	1	Others	CC1=C2CCCC(C3(C2=C(C=C1)C=C(C3=O)C(C)C)O)(C)C
DanShen	C24	miltionone Ⅰ	MOL007119	312.39	49.68	0.35	-0.11	0.32	41.49	C19H20O4	5319835	0.863	Good	NA	1	Diterpenoid Quinones	CC(C)C1=C(C2=C(C3=C(C=C2)C(CCC3=O)(C)C)C(=O)C1=O)O
DanShen	C25	miltionone Ⅱ	MOL007120	312.39	71.03	0.62	0.03	0.44	2.91	C19H20O4	5319836	0.747	Good	NA	1	Diterpenoid Quinones	CC1COC2=C1C(=O)C(=O)C3=C4CCCC(C4=C(C=C32)O)(C)C
DanShen	C26	Miltipolone	MOL007121	300.43	36.56	0.5	0.17	0.37	1.7	C19H24O3	10086184	0.748	Good	131086-61-8	1	Others	CC1=C(C(=O)C=C2C(=C1)C3CC4C2(CCCC4(C)C)CO3)O
DanShen	C27	Miltirone	MOL007122	282.41	38.76	1.23	0.87	0.25	14.82	C19H22O2	160142	0.688	Good	27210-57-7	1	Diterpenoid Quinones	CC(C)C1=CC2=C(C3=C(C=C2)C(CCC3)(C)C)C(=O)C1=O
DanShen	C28	neocryptotanshinone II	MOL007124	270.35	39.46	0.76	0.16	0.23	26.98	C17H18O3	5320066	0.783	Good	NA	1	Diterpenoid Quinones	CC1=C(C2=C(C3=C(C=C2)C(CCC3)(C)C)C(=O)C1=O)O
DanShen	C29	neocryptotanshinone	MOL007125	314.41	52.49	0.35	-0.13	0.32	14.46	C19H22O4	44425165	0.879	Good	NA	1	Diterpenoid Quinones	CC(CO)C1=C(C2=C(C3=C(C=C2)C(CCC3)(C)C)C(=O)C1=O)O
DanShen	C30	Nortanshinone	MOL007127	280.29	34.72	0.5	-0.27	0.37	37.89	C17H12O4	10062187	0.696	Good	97399-70-7	1	Diterpenoid Quinones	CC1=COC2=C1C(=O)C(=O)C3=C2C=CC4=C3CCCC4=O
DanShen	C31	Salviolone	MOL007145	268.38	31.72	1.04	0.72	0.24	0.33	C18H20O2	10355691	NA	NA	119400-86-1	1	Others	CC1=C(C(=O)C=C2C3=C(C=CC2=C1)C(CCC3)(C)C)O
DanShen	C32	Tanshinone IIA	MOL007154	294.37	49.89	1.05	0.7	0.4	23.56	C19H18O3	164676	0.663	Moderate	568-72-9	1	Diterpenoid Quinones	CC1=COC2=C1C(=O)C(=O)C3=C2C=CC4=C3CCCC4(C)C
DanShen	C33	tanshinone Ⅵ	MOL007156	296.34	45.64	0.48	-0.28	0.3	15.21	C18H16O4	98054352	0.835	Good	NA	1	Diterpenoid Quinones	CC1=C2C=CC3=C(C2=CC=C1)C(=O)C(=O)C(=C3O)C(C)CO
HongHua	C34	Lignan	MOL002695	458.55	43.32	0.42	-0.16	0.65	14.88	C25H30O8	261166	NA	NA	6549-68-4	1	Others	CCOC(=O)C1C(C(=O)C2=CC(=C(C=C2C1C3=CC(=C(C(=C3)OC)OC)OC)OC)OC)C
HongHua	C35	Pyrethrin II	MOL002710	372.5	48.36	0.53	-0.21	0.35	1.79	C22H28O5	5281555	0.349	Weak	121-29-9	1	Others	CC1=C(C(=O)CC1OC(=O)C2C(C2(C)C)C=C(C)C(=O)OC)CC=CC=C
HongHua	C36	Baicalein	MOL002714	270.25	33.52	0.63	-0.05	0.21	16.25	C15H10O5	5281605	0.428	Weak	491-67-8	1	Flavonoids	C1=CC=C(C=C1)C2=CC(=O)C3=C(O2)C=C(C(=C3O)O)O
HongHua	C37	Carthamidin	MOL002719	288.27	33.23	0.27	-0.27	0.24	15.67	C15H12O6	188308	0.599	Moderate	479-54-9	1	Flavonoids	C1C(OC2=C(C1=O)C(=C(C(=C2)O)O)O)C3=CC=C(C=C3)O
