# Supplementary material for: Pharmacological Mechanisms Underlying the Therapeutic Effects of Danhong Injection on Cerebral Ischemia
Source: Evid Based Complement Alternat Med. 2021 May 21;2021:5584809. doi: 10.1155/2021/5584809 (PMC8163534; doi:10.1155/2021/5584809)
Supplement: Supplementary Materials — Table S1: the 37 candidate compounds of Danhong injection. Table S2: the 371 putative target proteins for the compounds. Table S3: the 413 IS-associated Homo sapiens target proteins from CTD with an inference score of ≥50. Table S4: the 61 IS-associated target proteins of Homo sapiens from Genecards with an inference score of ≥30. Table S5: degree centrality of nodes in PPI network. Table S6: betweenness centrality of nodes in the PPI network. Table S7: the GO functional enrichment analysis of diterpenoid quinones. Table S8: the KEGG pathway enrichment of diterpenoid quinones. Table S9: the KEGG pathway enrichment of DHI compounds. [file 5584809.f1.zip › 5584809.f1/S2 (3).pdf]

Table S2. The 371 putative target proteins for the compounds

#N/A: present this uniprot ID lacks gene symbol, entrezID or ensembl in human.

Compound	Uniprot_ID	Protein name	Gene symbol	EntrezID	Ensembl	Organism	Classification
C1	P21918	D(1B) dopamine receptor	DRD5	1816	ENSG00000169676	Homo sapiens (Human)	Diterpenoid Quinones
C1	P55211	Caspase-9	CASP9	842	ENSG00000132906	Homo sapiens (Human)	Diterpenoid Quinones
C1	O00748	Cocaine esterase	CES2	8824	ENSG00000172831	Homo sapiens (Human)	Diterpenoid Quinones
C1	P22086	Alpha-2C adrenergic receptor	#N/A	#N/A	#N/A	Homo sapiens (Human)	Diterpenoid Quinones
C2	P16116	Aldose reductase	#N/A	#N/A	#N/A	Homo sapiens (Human)	Others
C2	P35218	Carbonic anhydrase 5A, mitochondrial	CA5A	763	ENSG00000174990	Homo sapiens (Human)	Others
C2	P27338	Amine oxidase [flavin-containing] B	MAOB	4129	ENSG00000069535	Homo sapiens (Human)	Others
C2	P19643	Amine oxidase [flavin-containing] B	#N/A	#N/A	#N/A	Homo sapiens (Human)	Others
C2	O43570	Carbonic anhydrase 12	CA12	771	ENSG00000074410	Homo sapiens (Human)	Others
C2	Q9ULX7	Carbonic anhydrase 14	CA14	23632	ENSG00000118298	Homo sapiens (Human)	Others
C2	P21397	Amine oxidase [flavin-containing] A	MAOA	4128	ENSG00000189221	Homo sapiens (Human)	Others
C2	P21396	Amine oxidase [flavin-containing] A	#N/A	#N/A	#N/A	Homo sapiens (Human)	Others
C2	Q16790	Carbonic anhydrase 9	CA9	768	ENSG00000107159	Homo sapiens (Human)	Others
C2	P14174	Macrophage migration inhibitory factor	MIF	4282	ENSG00000240972	Homo sapiens (Human)	Others
C2	P16050	Arachidonate 15-lipoxygenase	ALOX15	246	ENSG00000161905	Homo sapiens (Human)	Others
C2	Q9GZU7	Carboxy-terminal domain RNA polymerase II polypeptide A small phosphatase 1	CTDSP1	58190	ENSG00000144579	Homo sapiens (Human)	Others
C2	P22001	Potassium voltage-gated channel subfamily A member 3	KCNA3	3738	ENSG00000177272	Homo sapiens (Human)	Others
C2	P23141	Liver carboxylesterase 1	CES1	1066	ENSG00000198848	Homo sapiens (Human)	Others
C2	P35398	Nuclear receptor ROR-alpha	RORA	6095	ENSG00000069667	Homo sapiens (Human)	Others
C2	P43166	Carbonic anhydrase 7	CA7	766	ENSG00000168748	Homo sapiens (Human)	Others
C2	Q8N1Q1	Carbonic anhydrase 13	CA13	377677	ENSG00000185015	Homo sapiens (Human)	Others
C2	Q9Y2D0	Carbonic anhydrase 5B, mitochondrial	CA5B	11238	ENSG00000169239	Homo sapiens (Human)	Others
C2	P26358	DNA (cytosine-5)-methyltransferase 1	DNMT1	1786	ENSG00000130816	Homo sapiens (Human)	Others
C2	P22748	Carbonic anhydrase 4	CA4	762	ENSG00000167434	Homo sapiens (Human)	Others
C2	P23280	Carbonic anhydrase 6	CA6	765	ENSG00000131686	Homo sapiens (Human)	Others
C2	Q9NR96	Toll-like receptor 9	TLR9	54106	ENSG00000239732	Homo sapiens (Human)	Others
C2	P37058	Testosterone 17-beta-dehydrogenase 3	HSD17B3	3293	ENSG00000130948	Homo sapiens (Human)	Others
C3	P13631	Retinoic acid receptor gamma	RARG	5916	ENSG00000172819	Homo sapiens (Human)	Diterpenoid Quinones
C3	P23141	Liver carboxylesterase 1	CES1	1066	ENSG00000198848	Homo sapiens (Human)	Diterpenoid Quinones
C3	P04150	Glucocorticoid receptor	NR3C1	2908	ENSG00000113580	Homo sapiens (Human)	Diterpenoid Quinones
C3	P55211	Caspase-9	CASP9	842	ENSG00000132906	Homo sapiens (Human)	Diterpenoid Quinones
C3	O00748	Cocaine esterase	CES2	8824	ENSG00000172831	Homo sapiens (Human)	Diterpenoid Quinones
C3	Q16548	Bcl-2-related protein A1	BCL2A1	597	ENSG00000140379	Homo sapiens (Human)	Diterpenoid Quinones
C3	O43353	Receptor-interacting serine/threonine-protein kinase 2	RIPK2	8767	ENSG00000104312	Homo sapiens (Human)	Diterpenoid Quinones
C3	P30305	M-phase inducer phosphatase 2	CDC25B	994	ENSG00000101224	Homo sapiens (Human)	Diterpenoid Quinones
C3	P23280	Carbonic anhydrase 6	CA6	765	ENSG00000131686	Homo sapiens (Human)	Diterpenoid Quinones
C3	P05186	Alkaline phosphatase, tissue-nonspecific isozyme	ALPL	249	ENSG00000162551	Homo sapiens (Human)	Diterpenoid Quinones
C3	Q9NR96	Toll-like receptor 9	TLR9	54106	ENSG00000239732	Homo sapiens (Human)	Diterpenoid Quinones
C4	P31213	3-oxo-5-alpha-steroid 4-dehydrogenase 2	SRD5A2	6716	ENSG00000277893	Homo sapiens (Human)	Terpenes
C4	P03372	Estrogen receptor	ESR1	2099	ENSG00000091831	Homo sapiens (Human)	Terpenes
C4	P10827	Thyroid hormone receptor alpha	THRA	7067	ENSG00000126351	Homo sapiens (Human)	Terpenes
C4	P10826	Retinoic acid receptor beta	RARB	5915	ENSG00000077092	Homo sapiens (Human)	Terpenes
C4	P10276	Retinoic acid receptor alpha	RARA	5914	ENSG00000131759	Homo sapiens (Human)	Terpenes
C4	P13631	Retinoic acid receptor gamma	RARG	5916	ENSG00000172819	Homo sapiens (Human)	Terpenes
C4	P11511	Aromatase	CYP19A1	1588	ENSG00000137869	Homo sapiens (Human)	Terpenes
C4	P28702	Retinoic acid receptor RXR-beta	RXRB	6257	ENSG00000204231	Homo sapiens (Human)	Terpenes
C4	Q92731	Estrogen receptor beta	ESR2	2100	ENSG00000140009	Homo sapiens (Human)	Terpenes
C4	P05093	Steroid 17-alpha-hydroxylase/17,20 lyase	CYP17A1	1586	ENSG00000148795	Homo sapiens (Human)	Terpenes
C4	O42713	Polyphenol oxidase 2	#N/A	#N/A	#N/A	Homo sapiens (Human)	Terpenes
C4	O00748	Cocaine esterase	CES2	8824	ENSG00000172831	Homo sapiens (Human)	Terpenes
C4	P14061	Estradiol 17-beta-dehydrogenase 1	HSD17B1	3292	ENSG00000108786	Homo sapiens (Human)	Terpenes
C4	P48443	Retinoic acid receptor RXR-gamma	RXRG	6258	ENSG00000143171	Homo sapiens (Human)	Terpenes
C4	P30305	M-phase inducer phosphatase 2	CDC25B	994	ENSG00000101224	Homo sapiens (Human)	Terpenes
C5	P08575	Receptor-type tyrosine-protein phosphatase C	PTPRC	5788	ENSG00000081237	Homo sapiens (Human)	Diterpenoid Quinones
C5	P05177	Cytochrome P450 1A2	CYP1A2	1544	ENSG00000140505	Homo sapiens (Human)	Diterpenoid Quinones
C5	P05979	Prostaglandin G/H synthase 1	#N/A	#N/A	#N/A	Homo sapiens (Human)	Diterpenoid Quinones
C5	P14174	Macrophage migration inhibitory factor	MIF	4282	ENSG00000240972	Homo sapiens (Human)	Diterpenoid Quinones
C5	P23141	Liver carboxylesterase 1	CES1	1066	ENSG00000198848	Homo sapiens (Human)	Diterpenoid Quinones
C5	P55211	Caspase-9	CASP9	842	ENSG00000132906	Homo sapiens (Human)	Diterpenoid Quinones
C5	O00748	Cocaine esterase	CES2	8824	ENSG00000172831	Homo sapiens (Human)	Diterpenoid Quinones
C5	P30305	M-phase inducer phosphatase 2	CDC25B	994	ENSG00000101224	Homo sapiens (Human)	Diterpenoid Quinones
C5	Q9NR96	Toll-like receptor 9	TLR9	54106	ENSG00000239732	Homo sapiens (Human)	Diterpenoid Quinones
C6	P04150	Glucocorticoid receptor	NR3C1	2908	ENSG00000113580	Homo sapiens (Human)	Diterpenoid Quinones
C6	P55211	Caspase-9	CASP9	842	ENSG00000132906	Homo sapiens (Human)	Diterpenoid Quinones
C6	O00748	Cocaine esterase	CES2	8824	ENSG00000172831	Homo sapiens (Human)	Diterpenoid Quinones
C6	P30305	M-phase inducer phosphatase 2	CDC25B	994	ENSG00000101224	Homo sapiens (Human)	Diterpenoid Quinones
C6	P23280	Carbonic anhydrase 6	CA6	765	ENSG00000131686	Homo sapiens (Human)	Diterpenoid Quinones
C7	P04058	Acetylcholinesterase	#N/A	#N/A	#N/A	Homo sapiens (Human)	Diterpenoid Quinones
C7	P23141	Liver carboxylesterase 1	CES1	1066	ENSG00000198848	Homo sapiens (Human)	Diterpenoid Quinones
C7	P55211	Caspase-9	CASP9	842	ENSG00000132906	Homo sapiens (Human)	Diterpenoid Quinones
C7	O00748	Cocaine esterase	CES2	8824	ENSG00000172831	Homo sapiens (Human)	Diterpenoid Quinones
C7	P30305	M-phase inducer phosphatase 2	CDC25B	994	ENSG00000101224	Homo sapiens (Human)	Diterpenoid Quinones
C7	P23280	Carbonic anhydrase 6	CA6	765	ENSG00000131686	Homo sapiens (Human)	Diterpenoid Quinones
C7	Q9NR96	Toll-like receptor 9	TLR9	54106	ENSG00000239732	Homo sapiens (Human)	Diterpenoid Quinones
C8	Q07820	Induced myeloid leukemia cell differentiation protein Mcl-1	MCL1	4170	ENSG00000143384	Homo sapiens (Human)	Diterpenoid Quinones
C8	P21396	Amine oxidase [flavin-containing] A	#N/A	#N/A	#N/A	Homo sapiens (Human)	Diterpenoid Quinones
C8	P14174	Macrophage migration inhibitory factor	MIF	4282	ENSG00000240972	Homo sapiens (Human)	Diterpenoid Quinones
C8	Q9GZU7	Carboxy-terminal domain RNA polymerase II polypeptide A small phosphatase 1	CTDSP1	58190	ENSG00000144579	Homo sapiens (Human)	Diterpenoid Quinones
C8	P35869	Aryl hydrocarbon receptor	AHR	196	ENSG00000106546	Homo sapiens (Human)	Diterpenoid Quinones
C8	P23141	Liver carboxylesterase 1	CES1	1066	ENSG00000198848	Homo sapiens (Human)	Diterpenoid Quinones
C8	P55211	Caspase-9	CASP9	842	ENSG00000132906	Homo sapiens (Human)	Diterpenoid Quinones
C8	O00748	Cocaine esterase	CES2	8824	ENSG00000172831	Homo sapiens (Human)	Diterpenoid Quinones
C8	Q16548	Bcl-2-related protein A1	BCL2A1	597	ENSG00000140379	Homo sapiens (Human)	Diterpenoid Quinones
C8	P30305	M-phase inducer phosphatase 2	CDC25B	994	ENSG00000101224	Homo sapiens (Human)	Diterpenoid Quinones
C8	P11309	Serine/threonine-protein kinase pim-1	PIM1	5292	ENSG00000137193	Homo sapiens (Human)	Diterpenoid Quinones
C8	P26358	DNA (cytosine-5)-methyltransferase 1	DNMT1	1786	ENSG00000130816	Homo sapiens (Human)	Diterpenoid Quinones
C8	P16753	Capsid scaffolding protein	#N/A	#N/A	#N/A	Homo sapiens (Human)	Diterpenoid Quinones
C8	P23280	Carbonic anhydrase 6	CA6	765	ENSG00000131686	Homo sapiens (Human)	Diterpenoid Quinones
C8	P05186	Alkaline phosphatase, tissue-nonspecific isozyme	ALPL	249	ENSG00000162551	Homo sapiens (Human)	Diterpenoid Quinones
C8	Q9NR96	Toll-like receptor 9	TLR9	54106	ENSG00000239732	Homo sapiens (Human)	Diterpenoid Quinones
C9	P23141	Liver carboxylesterase 1	CES1	1066	ENSG00000198848	Homo sapiens (Human)	Diterpenoid Quinones
C9	P55211	Caspase-9	CASP9	842	ENSG00000132906	Homo sapiens (Human)	Diterpenoid Quinones
C9	O00748	Cocaine esterase	CES2	8824	ENSG00000172831	Homo sapiens (Human)	Diterpenoid Quinones
C9	P35398	Nuclear receptor ROR-alpha	RORA	6095	ENSG00000069667	Homo sapiens (Human)	Diterpenoid Quinones
C9	P22086	Alpha-2C adrenergic receptor	#N/A	#N/A	#N/A	Homo sapiens (Human)	Diterpenoid Quinones
C9	P30305	M-phase inducer phosphatase 2	CDC25B	994	ENSG00000101224	Homo sapiens (Human)	Diterpenoid Quinones
C9	P23280	Carbonic anhydrase 6	CA6	765	ENSG00000131686	Homo sapiens (Human)	Diterpenoid Quinones
C9	P05186	Alkaline phosphatase, tissue-nonspecific isozyme	ALPL	249	ENSG00000162551	Homo sapiens (Human)	Diterpenoid Quinones
C9	Q9NR96	Toll-like receptor 9	TLR9	54106	ENSG00000239732	Homo sapiens (Human)	Diterpenoid Quinones

C10	P05093	Steroid 17-alpha-hydroxylase/17,20 lyase	CYP17A1	1586	ENSG00000148795	Homo sapiens (Human)	Terpenes
C10	P29477	Nitric oxide synthase, inducible	#N/A	#N/A	#N/A	Homo sapiens (Human)	Terpenes
C11	P03372	Estrogen receptor	ESR1	2099	ENSG00000091831	Homo sapiens (Human)	Diterpenoid Quinones
C11	P06401	Progesterone receptor	PGR	5241	ENSG00000082175	Homo sapiens (Human)	Diterpenoid Quinones
C11	P13631	Retinoic acid receptor gamma	RARG	5916	ENSG00000172819	Homo sapiens (Human)	Diterpenoid Quinones
C11	P23141	Liver carboxylesterase 1	CES1	1066	ENSG00000198848	Homo sapiens (Human)	Diterpenoid Quinones
C11	P04150	Glucocorticoid receptor	NR3C1	2908	ENSG00000113580	Homo sapiens (Human)	Diterpenoid Quinones
C11	P55211	Caspase-9	CASP9	842	ENSG00000132906	Homo sapiens (Human)	Diterpenoid Quinones
C11	O00748	Cocaine esterase	CES2	8824	ENSG00000172831	Homo sapiens (Human)	Diterpenoid Quinones
C11	Q16548	Bcl-2-related protein A1	BCL2A1	597	ENSG00000140379	Homo sapiens (Human)	Diterpenoid Quinones
C11	P30305	M-phase inducer phosphatase 2	CDC25B	994	ENSG00000101224	Homo sapiens (Human)	Diterpenoid Quinones
C11	P23280	Carbonic anhydrase 6	CA6	765	ENSG00000131686	Homo sapiens (Human)	Diterpenoid Quinones
C12	P10275	Androgen receptor	AR	367	ENSG00000169083	Homo sapiens (Human)	Terpenes
C12	P04058	Acetylcholinesterase	#N/A	#N/A	#N/A	Homo sapiens (Human)	Terpenes
C12	P15207	Androgen receptor	#N/A	#N/A	#N/A	Homo sapiens (Human)	Terpenes
C12	P08173	Muscarinic acetylcholine receptor M4	CHRM4	1132	ENSG00000180720	Homo sapiens (Human)	Terpenes
C12	O43353	Receptor-interacting serine/threonine-protein kinase 2	RIPK2	8767	ENSG00000104312	Homo sapiens (Human)	Terpenes
C12	P30305	M-phase inducer phosphatase 2	CDC25B	994	ENSG00000101224	Homo sapiens (Human)	Terpenes
C13	P06401	Progesterone receptor	PGR	5241	ENSG00000082175	Homo sapiens (Human)	Diterpenoid Quinones
C13	P04150	Glucocorticoid receptor	NR3C1	2908	ENSG00000113580	Homo sapiens (Human)	Diterpenoid Quinones
C13	P55211	Caspase-9	CASP9	842	ENSG00000132906	Homo sapiens (Human)	Diterpenoid Quinones
C13	O00748	Cocaine esterase	CES2	8824	ENSG00000172831	Homo sapiens (Human)	Diterpenoid Quinones
C13	P22086	Alpha-2C adrenergic receptor	#N/A	#N/A	#N/A	Homo sapiens (Human)	Diterpenoid Quinones
C13	P30305	M-phase inducer phosphatase 2	CDC25B	994	ENSG00000101224	Homo sapiens (Human)	Diterpenoid Quinones
C14	P10826	Retinoic acid receptor beta	RARB	5915	ENSG00000077092	Homo sapiens (Human)	Diterpenoid Quinones
C14	P10276	Retinoic acid receptor alpha	RARA	5914	ENSG00000131759	Homo sapiens (Human)	Diterpenoid Quinones
C14	P13631	Retinoic acid receptor gamma	RARG	5916	ENSG00000172819	Homo sapiens (Human)	Diterpenoid Quinones
C14	P28702	Retinoic acid receptor RXR-beta	RXRB	6257	ENSG00000204231	Homo sapiens (Human)	Diterpenoid Quinones
C14	Q9QZN9	Cannabinoid receptor 2	#N/A	#N/A	#N/A	Homo sapiens (Human)	Diterpenoid Quinones
C14	O00748	Cocaine esterase	CES2	8824	ENSG00000172831	Homo sapiens (Human)	Diterpenoid Quinones
C14	P48443	Retinoic acid receptor RXR-gamma	RXRG	6258	ENSG00000143171	Homo sapiens (Human)	Diterpenoid Quinones
C15	Q9QZN9	Cannabinoid receptor 2	#N/A	#N/A	#N/A	Homo sapiens (Human)	Diterpenoid Quinones
C15	O00748	Cocaine esterase	CES2	8824	ENSG00000172831	Homo sapiens (Human)	Diterpenoid Quinones
C16	P15121	Aldose reductase	AKR1B1	231	ENSG00000085662	Homo sapiens (Human)	Diterpenoid Quinones
C16	P06401	Progesterone receptor	PGR	5241	ENSG00000082175	Homo sapiens (Human)	Diterpenoid Quinones
C16	Q9HC16	DNA dC->dU-editing enzyme APOBEC-3G	APOBEC3G	60489	ENSG00000239713	Homo sapiens (Human)	Diterpenoid Quinones
C16	P04058	Acetylcholinesterase	#N/A	#N/A	#N/A	Homo sapiens (Human)	Diterpenoid Quinones
C16	P55211	Caspase-9	CASP9	842	ENSG00000132906	Homo sapiens (Human)	Diterpenoid Quinones
C16	O00748	Cocaine esterase	CES2	8824	ENSG00000172831	Homo sapiens (Human)	Diterpenoid Quinones
C16	O43353	Receptor-interacting serine/threonine-protein kinase 2	RIPK2	8767	ENSG00000104312	Homo sapiens (Human)	Diterpenoid Quinones
C16	P30305	M-phase inducer phosphatase 2	CDC25B	994	ENSG00000101224	Homo sapiens (Human)	Diterpenoid Quinones
C16	Q9NR96	Toll-like receptor 9	TLR9	54106	ENSG00000239732	Homo sapiens (Human)	Diterpenoid Quinones
C17	P10276	Retinoic acid receptor alpha	RARA	5914	ENSG00000131759	Homo sapiens (Human)	Diterpenoid Quinones
C17	P06401	Progesterone receptor	PGR	5241	ENSG00000082175	Homo sapiens (Human)	Diterpenoid Quinones
C17	P13631	Retinoic acid receptor gamma	RARG	5916	ENSG00000172819	Homo sapiens (Human)	Diterpenoid Quinones
C17	Q9QZN9	Cannabinoid receptor 2	#N/A	#N/A	#N/A	Homo sapiens (Human)	Diterpenoid Quinones
C17	P28566	5-hydroxytryptamine receptor 1E	HTR1E	3354	ENSG00000168830	Homo sapiens (Human)	Diterpenoid Quinones
C17	P23141	Liver carboxylesterase 1	CES1	1066	ENSG00000198848	Homo sapiens (Human)	Diterpenoid Quinones
C17	P04150	Glucocorticoid receptor	NR3C1	2908	ENSG00000113580	Homo sapiens (Human)	Diterpenoid Quinones
C17	O00748	Cocaine esterase	CES2	8824	ENSG00000172831	Homo sapiens (Human)	Diterpenoid Quinones
C17	P30305	M-phase inducer phosphatase 2	CDC25B	994	ENSG00000101224	Homo sapiens (Human)	Diterpenoid Quinones
C18	Q9QZN9	Cannabinoid receptor 2	#N/A	#N/A	#N/A	Homo sapiens (Human)	Diterpenoid Quinones
C18	P55211	Caspase-9	CASP9	842	ENSG00000132906	Homo sapiens (Human)	Diterpenoid Quinones
C19	P10826	Retinoic acid receptor beta	RARB	5915	ENSG00000077092	Homo sapiens (Human)	Diterpenoid Quinones
C19	P10275	Androgen receptor	AR	367	ENSG00000169083	Homo sapiens (Human)	Diterpenoid Quinones
C19	P10276	Retinoic acid receptor alpha	RARA	5914	ENSG00000131759	Homo sapiens (Human)	Diterpenoid Quinones
C19	P06401	Progesterone receptor	PGR	5241	ENSG00000082175	Homo sapiens (Human)	Diterpenoid Quinones
C19	P13631	Retinoic acid receptor gamma	RARG	5916	ENSG00000172819	Homo sapiens (Human)	Diterpenoid Quinones
C19	P28702	Retinoic acid receptor RXR-beta	RXRB	6257	ENSG00000204231	Homo sapiens (Human)	Diterpenoid Quinones
C19	P19793	Retinoic acid receptor RXR-alpha	RXRA	6256	ENSG00000186350	Homo sapiens (Human)	Diterpenoid Quinones
C19	P04058	Acetylcholinesterase	#N/A	#N/A	#N/A	Homo sapiens (Human)	Diterpenoid Quinones
C19	P23141	Liver carboxylesterase 1	CES1	1066	ENSG00000198848	Homo sapiens (Human)	Diterpenoid Quinones
C19	O00748	Cocaine esterase	CES2	8824	ENSG00000172831	Homo sapiens (Human)	Diterpenoid Quinones
C19	P48443	Retinoic acid receptor RXR-gamma	RXRG	6258	ENSG00000143171	Homo sapiens (Human)	Diterpenoid Quinones
C19	P30305	M-phase inducer phosphatase 2	CDC25B	994	ENSG00000101224	Homo sapiens (Human)	Diterpenoid Quinones
C20	P23141	Liver carboxylesterase 1	CES1	1066	ENSG00000198848	Homo sapiens (Human)	Diterpenoid Quinones
C20	P55211	Caspase-9	CASP9	842	ENSG00000132906	Homo sapiens (Human)	Diterpenoid Quinones
C20	O00748	Cocaine esterase	CES2	8824	ENSG00000172831	Homo sapiens (Human)	Diterpenoid Quinones
C20	P23280	Carbonic anhydrase 6	CA6	765	ENSG00000131686	Homo sapiens (Human)	Diterpenoid Quinones
C21	P10275	Androgen receptor	AR	367	ENSG00000169083	Homo sapiens (Human)	Diterpenoid Quinones
C21	P10276	Retinoic acid receptor alpha	RARA	5914	ENSG00000131759	Homo sapiens (Human)	Diterpenoid Quinones
C21	P06401	Progesterone receptor	PGR	5241	ENSG00000082175	Homo sapiens (Human)	Diterpenoid Quinones
C21	P13631	Retinoic acid receptor gamma	RARG	5916	ENSG00000172819	Homo sapiens (Human)	Diterpenoid Quinones
C21	Q9QZN9	Cannabinoid receptor 2	#N/A	#N/A	#N/A	Homo sapiens (Human)	Diterpenoid Quinones
C21	P55211	Caspase-9	CASP9	842	ENSG00000132906	Homo sapiens (Human)	Diterpenoid Quinones
C21	O00748	Cocaine esterase	CES2	8824	ENSG00000172831	Homo sapiens (Human)	Diterpenoid Quinones
C21	P22086	Alpha-2C adrenergic receptor	#N/A	#N/A	#N/A	Homo sapiens (Human)	Diterpenoid Quinones
C21	P07900	Heat shock protein HSP 90-alpha	HSP90AA1	3320	ENSG00000080824	Homo sapiens (Human)	Diterpenoid Quinones
C21	P30305	M-phase inducer phosphatase 2	CDC25B	994	ENSG00000101224	Homo sapiens (Human)	Diterpenoid Quinones
C22	P10276	Retinoic acid receptor alpha	RARA	5914	ENSG00000131759	Homo sapiens (Human)	Diterpenoid Quinones
C22	P06401	Progesterone receptor	PGR	5241	ENSG00000082175	Homo sapiens (Human)	Diterpenoid Quinones
C22	P13631	Retinoic acid receptor gamma	RARG	5916	ENSG00000172819	Homo sapiens (Human)	Diterpenoid Quinones
C22	Q9QZN9	Cannabinoid receptor 2	#N/A	#N/A	#N/A	Homo sapiens (Human)	Diterpenoid Quinones
C22	P28566	5-hydroxytryptamine receptor 1E	HTR1E	3354	ENSG00000168830	Homo sapiens (Human)	Diterpenoid Quinones
C22	P23141	Liver carboxylesterase 1	CES1	1066	ENSG00000198848	Homo sapiens (Human)	Diterpenoid Quinones
C22	P55211	Caspase-9	CASP9	842	ENSG00000132906	Homo sapiens (Human)	Diterpenoid Quinones
C22	P31388	5-hydroxytryptamine receptor 6	#N/A	#N/A	#N/A	Homo sapiens (Human)	Diterpenoid Quinones
C22	O00748	Cocaine esterase	CES2	8824	ENSG00000172831	Homo sapiens (Human)	Diterpenoid Quinones
C22	P30305	M-phase inducer phosphatase 2	CDC25B	994	ENSG00000101224	Homo sapiens (Human)	Diterpenoid Quinones
C23	P10826	Retinoic acid receptor beta	RARB	5915	ENSG00000077092	Homo sapiens (Human)	Others
C23	P10276	Retinoic acid receptor alpha	RARA	5914	ENSG00000131759	Homo sapiens (Human)	Others
C23	P05093	Steroid 17-alpha-hydroxylase/17,20 lyase	CYP17A1	1586	ENSG00000148795	Homo sapiens (Human)	Others
C23	P04058	Acetylcholinesterase	#N/A	#N/A	#N/A	Homo sapiens (Human)	Others
C23	P55211	Caspase-9	CASP9	842	ENSG00000132906	Homo sapiens (Human)	Others
C23	O00748	Cocaine esterase	CES2	8824	ENSG00000172831	Homo sapiens (Human)	Others
C23	P30305	M-phase inducer phosphatase 2	CDC25B	994	ENSG00000101224	Homo sapiens (Human)	Others
C24	P31213	3-oxo-5-alpha-steroid 4-dehydrogenase 2	SRD5A2	6716	ENSG00000277893	Homo sapiens (Human)	Diterpenoid Quinones
C24	P03372	Estrogen receptor	ESR1	2099	ENSG00000091831	Homo sapiens (Human)	Diterpenoid Quinones
C24	P10826	Retinoic acid receptor beta	RARB	5915	ENSG00000077092	Homo sapiens (Human)	Diterpenoid Quinones
C24	P10275	Androgen receptor	AR	367	ENSG00000169083	Homo sapiens (Human)	Diterpenoid Quinones
C24	P10276	Retinoic acid receptor alpha	RARA	5914	ENSG00000131759	Homo sapiens (Human)	Diterpenoid Quinones
C24	P13631	Retinoic acid receptor gamma	RARG	5916	ENSG00000172819	Homo sapiens (Human)	Diterpenoid Quinones
C24	P11511	Aromatase	CYP19A1	1588	ENSG00000137869	Homo sapiens (Human)	Diterpenoid Quinones
C24	P28702	Retinoic acid receptor RXR-beta	RXRB	6257	ENSG00000204231	Homo sapiens (Human)	Diterpenoid Quinones
C24	Q92731	Estrogen receptor beta	ESR2	2100	ENSG00000140009	Homo sapiens (Human)	Diterpenoid Quinones

C24	P19793	Retinoic acid receptor RXR-alpha	RXRA	6256	ENSG00000186350	Homo sapiens (Human)	Diterpenoid Quinones
C24	P23219	Prostaglandin G/H synthase 1	PTGS1	5742	ENSG00000095303	Homo sapiens (Human)	Diterpenoid Quinones
C24	O42713	Polyphenol oxidase 2	#N/A	#N/A	#N/A	Homo sapiens (Human)	Diterpenoid Quinones
C24	P15207	Androgen receptor	#N/A	#N/A	#N/A	Homo sapiens (Human)	Diterpenoid Quinones
C24	P18405	3-oxo-5-alpha-steroid 4-dehydrogenase 1	SRD5A1	6715	ENSG00000145545	Homo sapiens (Human)	Diterpenoid Quinones
C24	O00748	Cocaine esterase	CES2	8824	ENSG00000172831	Homo sapiens (Human)	Diterpenoid Quinones
C24	P48443	Retinoic acid receptor RXR-gamma	RXRG	6258	ENSG00000143171	Homo sapiens (Human)	Diterpenoid Quinones
C24	P30305	M-phase inducer phosphatase 2	CDC25B	994	ENSG00000101224	Homo sapiens (Human)	Diterpenoid Quinones
C24	Q9NR96	Toll-like receptor 9	TLR9	54106	ENSG00000239732	Homo sapiens (Human)	Diterpenoid Quinones
C25	P08842	Steryl-sulfatase	STS	412	ENSG00000101846	Homo sapiens (Human)	Diterpenoid Quinones
C25	P10275	Androgen receptor	AR	367	ENSG00000169083	Homo sapiens (Human)	Diterpenoid Quinones
C25	P29477	Nitric oxide synthase, inducible	#N/A	#N/A	#N/A	Homo sapiens (Human)	Diterpenoid Quinones
C25	O42713	Polyphenol oxidase 2	#N/A	#N/A	#N/A	Homo sapiens (Human)	Diterpenoid Quinones
C25	P16050	Arachidonate 15-lipoxygenase	ALOX15	246	ENSG00000161905	Homo sapiens (Human)	Diterpenoid Quinones
C25	P23141	Liver carboxylesterase 1	CES1	1066	ENSG00000198848	Homo sapiens (Human)	Diterpenoid Quinones
C25	O00748	Cocaine esterase	CES2	8824	ENSG00000172831	Homo sapiens (Human)	Diterpenoid Quinones
C25	P22086	Alpha-2C adrenergic receptor	#N/A	#N/A	#N/A	Homo sapiens (Human)	Diterpenoid Quinones
C25	P14061	Estradiol 17-beta-dehydrogenase 1	HSD17B1	3292	ENSG00000108786	Homo sapiens (Human)	Diterpenoid Quinones
C25	P30305	M-phase inducer phosphatase 2	CDC25B	994	ENSG00000101224	Homo sapiens (Human)	Diterpenoid Quinones
C25	P23280	Carbonic anhydrase 6	CA6	765	ENSG00000131686	Homo sapiens (Human)	Diterpenoid Quinones
C26	P05177	Cytochrome P450 1A2	CYP1A2	1544	ENSG00000140505	Homo sapiens (Human)	Others
C26	P04058	Acetylcholinesterase	#N/A	#N/A	#N/A	Homo sapiens (Human)	Others
C26	P16050	Arachidonate 15-lipoxygenase	ALOX15	246	ENSG00000161905	Homo sapiens (Human)	Others
C26	P33261	Cytochrome P450 2C19	CYP2C19	1557	ENSG00000165841	Homo sapiens (Human)	Others
C27	P10826	Retinoic acid receptor beta	RARB	5915	ENSG00000077092	Homo sapiens (Human)	Diterpenoid Quinones
C27	P10276	Retinoic acid receptor alpha	RARA	5914	ENSG00000131759	Homo sapiens (Human)	Diterpenoid Quinones
C27	P06401	Progesterone receptor	PGR	5241	ENSG00000082175	Homo sapiens (Human)	Diterpenoid Quinones
C27	P13631	Retinoic acid receptor gamma	RARG	5916	ENSG00000172819	Homo sapiens (Human)	Diterpenoid Quinones
C27	P28702	Retinoic acid receptor RXR-beta	RXRB	6257	ENSG00000204231	Homo sapiens (Human)	Diterpenoid Quinones
C27	P19793	Retinoic acid receptor RXR-alpha	RXRA	6256	ENSG00000186350	Homo sapiens (Human)	Diterpenoid Quinones
C27	P04058	Acetylcholinesterase	#N/A	#N/A	#N/A	Homo sapiens (Human)	Diterpenoid Quinones
C27	P23141	Liver carboxylesterase 1	CES1	1066	ENSG00000198848	Homo sapiens (Human)	Diterpenoid Quinones
C27	O00748	Cocaine esterase	CES2	8824	ENSG00000172831	Homo sapiens (Human)	Diterpenoid Quinones
C27	P48443	Retinoic acid receptor RXR-gamma	RXRG	6258	ENSG00000143171	Homo sapiens (Human)	Diterpenoid Quinones
C27	P30305	M-phase inducer phosphatase 2	CDC25B	994	ENSG00000101224	Homo sapiens (Human)	Diterpenoid Quinones
C28	P03372	Estrogen receptor	ESR1	2099	ENSG00000091831	Homo sapiens (Human)	Diterpenoid Quinones
C28	P10826	Retinoic acid receptor beta	RARB	5915	ENSG00000077092	Homo sapiens (Human)	Diterpenoid Quinones
C28	P10275	Androgen receptor	AR	367	ENSG00000169083	Homo sapiens (Human)	Diterpenoid Quinones
C28	P10276	Retinoic acid receptor alpha	RARA	5914	ENSG00000131759	Homo sapiens (Human)	Diterpenoid Quinones
C28	P06401	Progesterone receptor	PGR	5241	ENSG00000082175	Homo sapiens (Human)	Diterpenoid Quinones
C28	P13631	Retinoic acid receptor gamma	RARG	5916	ENSG00000172819	Homo sapiens (Human)	Diterpenoid Quinones
C28	P28702	Retinoic acid receptor RXR-beta	RXRB	6257	ENSG00000204231	Homo sapiens (Human)	Diterpenoid Quinones
C28	P19793	Retinoic acid receptor RXR-alpha	RXRA	6256	ENSG00000186350	Homo sapiens (Human)	Diterpenoid Quinones
C28	P04058	Acetylcholinesterase	#N/A	#N/A	#N/A	Homo sapiens (Human)	Diterpenoid Quinones
C28	P23141	Liver carboxylesterase 1	CES1	1066	ENSG00000198848	Homo sapiens (Human)	Diterpenoid Quinones
C28	P55211	Caspase-9	CASP9	842	ENSG00000132906	Homo sapiens (Human)	Diterpenoid Quinones
C28	O00748	Cocaine esterase	CES2	8824	ENSG00000172831	Homo sapiens (Human)	Diterpenoid Quinones
C28	P48443	Retinoic acid receptor RXR-gamma	RXRG	6258	ENSG00000143171	Homo sapiens (Human)	Diterpenoid Quinones
C28	P30305	M-phase inducer phosphatase 2	CDC25B	994	ENSG00000101224	Homo sapiens (Human)	Diterpenoid Quinones
C29	P10276	Retinoic acid receptor alpha	RARA	5914	ENSG00000131759	Homo sapiens (Human)	Diterpenoid Quinones
C29	P06401	Progesterone receptor	PGR	5241	ENSG00000082175	Homo sapiens (Human)	Diterpenoid Quinones
C29	P13631	Retinoic acid receptor gamma	RARG	5916	ENSG00000172819	Homo sapiens (Human)	Diterpenoid Quinones
C29	P19793	Retinoic acid receptor RXR-alpha	RXRA	6256	ENSG00000186350	Homo sapiens (Human)	Diterpenoid Quinones
C29	P28566	5-hydroxytryptamine receptor 1E	HTR1E	3354	ENSG00000168830	Homo sapiens (Human)	Diterpenoid Quinones
C29	O00748	Cocaine esterase	CES2	8824	ENSG00000172831	Homo sapiens (Human)	Diterpenoid Quinones
C29	P30305	M-phase inducer phosphatase 2	CDC25B	994	ENSG00000101224	Homo sapiens (Human)	Diterpenoid Quinones
C30	P23219	Prostaglandin G/H synthase 1	PTGS1	5742	ENSG00000095303	Homo sapiens (Human)	Diterpenoid Quinones
C30	P23141	Liver carboxylesterase 1	CES1	1066	ENSG00000198848	Homo sapiens (Human)	Diterpenoid Quinones
C30	P55211	Caspase-9	CASP9	842	ENSG00000132906	Homo sapiens (Human)	Diterpenoid Quinones
C30	O00748	Cocaine esterase	CES2	8824	ENSG00000172831	Homo sapiens (Human)	Diterpenoid Quinones
C30	Q16548	Bcl-2-related protein A1	BCL2A1	597	ENSG00000140379	Homo sapiens (Human)	Diterpenoid Quinones
C30	P30305	M-phase inducer phosphatase 2	CDC25B	994	ENSG00000101224	Homo sapiens (Human)	Diterpenoid Quinones
C30	P23280	Carbonic anhydrase 6	CA6	765	ENSG00000131686	Homo sapiens (Human)	Diterpenoid Quinones
C30	P05186	Alkaline phosphatase, tissue-nonspecific isozyme	ALPL	249	ENSG00000162551	Homo sapiens (Human)	Diterpenoid Quinones
C30	Q9NR96	Toll-like receptor 9	TLR9	54106	ENSG00000239732	Homo sapiens (Human)	Diterpenoid Quinones
C31	P03372	Estrogen receptor	ESR1	2099	ENSG00000091831	Homo sapiens (Human)	Others
C31	P10826	Retinoic acid receptor beta	RARB	5915	ENSG00000077092	Homo sapiens (Human)	Others
C31	P10276	Retinoic acid receptor alpha	RARA	5914	ENSG00000131759	Homo sapiens (Human)	Others
C31	P15121	Aldose reductase	AKR1B1	231	ENSG00000085662	Homo sapiens (Human)	Others
C31	P13631	Retinoic acid receptor gamma	RARG	5916	ENSG00000172819	Homo sapiens (Human)	Others
C31	P11511	Aromatase	CYP19A1	1588	ENSG00000137869	Homo sapiens (Human)	Others
C31	P28702	Retinoic acid receptor RXR-beta	RXRB	6257	ENSG00000204231	Homo sapiens (Human)	Others
C31	Q92731	Estrogen receptor beta	ESR2	2100	ENSG00000140009	Homo sapiens (Human)	Others
C31	P29477	Nitric oxide synthase, inducible	#N/A	#N/A	#N/A	Homo sapiens (Human)	Others
C31	P14174	Macrophage migration inhibitory factor	MIF	4282	ENSG00000240972	Homo sapiens (Human)	Others
C31	O42713	Polyphenol oxidase 2	#N/A	#N/A	#N/A	Homo sapiens (Human)	Others
C31	P28564	5-hydroxytryptamine receptor 1B	#N/A	#N/A	#N/A	Homo sapiens (Human)	Others
C31	O00748	Cocaine esterase	CES2	8824	ENSG00000172831	Homo sapiens (Human)	Others
C31	P22086	Alpha-2C adrenergic receptor	#N/A	#N/A	#N/A	Homo sapiens (Human)	Others
C31	P14061	Estradiol 17-beta-dehydrogenase 1	HSD17B1	3292	ENSG00000108786	Homo sapiens (Human)	Others
C31	P30305	M-phase inducer phosphatase 2	CDC25B	994	ENSG00000101224	Homo sapiens (Human)	Others
C31	P37058	Testosterone 17-beta-dehydrogenase 3	HSD17B3	3293	ENSG00000130948	Homo sapiens (Human)	Others
C32	P10826	Retinoic acid receptor beta	RARB	5915	ENSG00000077092	Homo sapiens (Human)	Diterpenoid Quinones
C32	P10276	Retinoic acid receptor alpha	RARA	5914	ENSG00000131759	Homo sapiens (Human)	Diterpenoid Quinones
C32	P06401	Progesterone receptor	PGR	5241	ENSG00000082175	Homo sapiens (Human)	Diterpenoid Quinones
C32	P13631	Retinoic acid receptor gamma	RARG	5916	ENSG00000172819	Homo sapiens (Human)	Diterpenoid Quinones
C32	P28702	Retinoic acid receptor RXR-beta	RXRB	6257	ENSG00000204231	Homo sapiens (Human)	Diterpenoid Quinones
C32	P19793	Retinoic acid receptor RXR-alpha	RXRA	6256	ENSG00000186350	Homo sapiens (Human)	Diterpenoid Quinones
C32	P28566	5-hydroxytryptamine receptor 1E	HTR1E	3354	ENSG00000168830	Homo sapiens (Human)	Diterpenoid Quinones
C32	P23141	Liver carboxylesterase 1	CES1	1066	ENSG00000198848	Homo sapiens (Human)	Diterpenoid Quinones
C32	P55211	Caspase-9	CASP9	842	ENSG00000132906	Homo sapiens (Human)	Diterpenoid Quinones
C32	O00748	Cocaine esterase	CES2	8824	ENSG00000172831	Homo sapiens (Human)	Diterpenoid Quinones
C32	P30305	M-phase inducer phosphatase 2	CDC25B	994	ENSG00000101224	Homo sapiens (Human)	Diterpenoid Quinones
C32	P23280	Carbonic anhydrase 6	CA6	765	ENSG00000131686	Homo sapiens (Human)	Diterpenoid Quinones
C33	Q07820	Induced myeloid leukemia cell differentiation protein Mcl-1	MCL1	4170	ENSG00000143384	Homo sapiens (Human)	Diterpenoid Quinones
C33	P55211	Caspase-9	CASP9	842	ENSG00000132906	Homo sapiens (Human)	Diterpenoid Quinones
C33	O00748	Cocaine esterase	CES2	8824	ENSG00000172831	Homo sapiens (Human)	Diterpenoid Quinones
C33	P30305	M-phase inducer phosphatase 2	CDC25B	994	ENSG00000101224	Homo sapiens (Human)	Diterpenoid Quinones
C34	Q6B856	Tubulin beta-2B chain	#N/A	#N/A	#N/A	Homo sapiens (Human)	Others
C34	P05979	Prostaglandin G/H synthase 1	#N/A	#N/A	#N/A	Homo sapiens (Human)	Others
C34	P04058	Acetylcholinesterase	#N/A	#N/A	#N/A	Homo sapiens (Human)	Others
C34	Q3KRE8	Tubulin beta-2B chain	#N/A	#N/A	#N/A	Homo sapiens (Human)	Others
C34	Q14432	cGMP-inhibited 3',5'-cyclic phosphodiesterase A	PDE3A	5139	ENSG00000172572	Homo sapiens (Human)	Others
C34	P35398	Nuclear receptor ROR-alpha	RORA	6095	ENSG00000069667	Homo sapiens (Human)	Others
C34	P02550	Tubulin alpha-1A chain	#N/A	#N/A	#N/A	Homo sapiens (Human)	Others
C35	P29477	Nitric oxide synthase, inducible	#N/A	#N/A	#N/A	Homo sapiens (Human)	Others

C35	P04058	Acetylcholinesterase	#N/A	#N/A	#N/A	Homo sapiens (Human)	Others
C36	P03372	Estrogen receptor	ESR1	2099	ENSG00000091831	Homo sapiens (Human)	Flavonoids
C36	P08183	Multidrug resistance protein 1	ABCB1	5243	ENSG00000085563	Homo sapiens (Human)	Flavonoids
C36	P79208	Prostaglandin G/H synthase 2	#N/A	#N/A	#N/A	Homo sapiens (Human)	Flavonoids
C36	Q9Y2R2	Tyrosine-protein phosphatase non-receptor type 22	PTPN22	26191	ENSG00000134242	Homo sapiens (Human)	Flavonoids
C36	P05177	Cytochrome P450 1A2	CYP1A2	1544	ENSG00000140505	Homo sapiens (Human)	Flavonoids
C36	P63001	Ras-related C3 botulinum toxin substrate 1	#N/A	#N/A	#N/A	Homo sapiens (Human)	Flavonoids
C36	P16116	Aldose reductase	#N/A	#N/A	#N/A	Homo sapiens (Human)	Flavonoids
C36	P15121	Aldose reductase	AKR1B1	231	ENSG00000085662	Homo sapiens (Human)	Flavonoids
C36	P51452	Dual specificity protein phosphatase 3	DUSP3	1845	ENSG00000108861	Homo sapiens (Human)	Flavonoids
C36	Q04206	Transcription factor p65	RELA	5970	ENSG00000173039	Homo sapiens (Human)	Flavonoids
C36	P11511	Aromatase	CYP19A1	1588	ENSG00000137869	Homo sapiens (Human)	Flavonoids
C36	Q92731	Estrogen receptor beta	ESR2	2100	ENSG00000140009	Homo sapiens (Human)	Flavonoids
C36	P80457	Xanthine dehydrogenase/oxidase	#N/A	#N/A	#N/A	Homo sapiens (Human)	Flavonoids
C36	P27338	Amine oxidase [flavin-containing] B	MAOB	4129	ENSG00000069535	Homo sapiens (Human)	Flavonoids
C36	P29477	Nitric oxide synthase, inducible	#N/A	#N/A	#N/A	Homo sapiens (Human)	Flavonoids
C36	P21397	Amine oxidase [flavin-containing] A	MAOA	4128	ENSG00000189221	Homo sapiens (Human)	Flavonoids
C36	P05979	Prostaglandin G/H synthase 1	#N/A	#N/A	#N/A	Homo sapiens (Human)	Flavonoids
C36	P23219	Prostaglandin G/H synthase 1	PTGS1	5742	ENSG00000095303	Homo sapiens (Human)	Flavonoids
C36	P14174	Macrophage migration inhibitory factor	MIF	4282	ENSG00000240972	Homo sapiens (Human)	Flavonoids
C36	O42713	Polyphenol oxidase 2	#N/A	#N/A	#N/A	Homo sapiens (Human)	Flavonoids
C36	P16050	Arachidonate 15-lipoxygenase	ALOX15	246	ENSG00000161905	Homo sapiens (Human)	Flavonoids
C36	Q9GZU7	Carboxy-terminal domain RNA polymerase II polypeptide A small phosphatase 1	CTDSP1	58190	ENSG00000144579	Homo sapiens (Human)	Flavonoids
C36	P35869	Aryl hydrocarbon receptor	AHR	196	ENSG00000106546	Homo sapiens (Human)	Flavonoids
C36	P23141	Liver carboxylesterase 1	CES1	1066	ENSG00000198848	Homo sapiens (Human)	Flavonoids
C36	Q9Y5X4	Photoreceptor-specific nuclear receptor	NR2E3	10002	ENSG00000278570	Homo sapiens (Human)	Flavonoids
C36	O00748	Cocaine esterase	CES2	8824	ENSG00000172831	Homo sapiens (Human)	Flavonoids
C36	Q16548	Bcl-2-related protein A1	BCL2A1	597	ENSG00000140379	Homo sapiens (Human)	Flavonoids
C36	Q9UNQ0	ATP-binding cassette sub-family G member 2	ABCG2	9429	ENSG00000118777	Homo sapiens (Human)	Flavonoids
C36	P43166	Carbonic anhydrase 7	CA7	766	ENSG00000168748	Homo sapiens (Human)	Flavonoids
C36	P14061	Estradiol 17-beta-dehydrogenase 1	HSD17B1	3292	ENSG00000108786	Homo sapiens (Human)	Flavonoids
C36	P12527	Arachidonate 5-lipoxygenase	#N/A	#N/A	#N/A	Homo sapiens (Human)	Flavonoids
C36	P07943	Aldose reductase	#N/A	#N/A	#N/A	Homo sapiens (Human)	Flavonoids
C36	Q8N1Q1	Carbonic anhydrase 13	CA13	377677	ENSG00000185015	Homo sapiens (Human)	Flavonoids
C36	O14746	Telomerase reverse transcriptase	TERT	7015	ENSG00000164362	Homo sapiens (Human)	Flavonoids
C36	P11309	Serine/threonine-protein kinase pim-1	PIM1	5292	ENSG00000137193	Homo sapiens (Human)	Flavonoids
C36	P23280	Carbonic anhydrase 6	CA6	765	ENSG00000131686	Homo sapiens (Human)	Flavonoids
C36	Q9NR96	Toll-like receptor 9	TLR9	54106	ENSG00000239732	Homo sapiens (Human)	Flavonoids
C36	P37059	Estradiol 17-beta-dehydrogenase 2	HSD17B2	3294	ENSG00000086696	Homo sapiens (Human)	Flavonoids
C37	P03372	Estrogen receptor	ESR1	2099	ENSG00000091831	Homo sapiens (Human)	Flavonoids
C37	P08842	Steryl-sulfatase	STS	412	ENSG00000101846	Homo sapiens (Human)	Flavonoids
C37	P15121	Aldose reductase	AKR1B1	231	ENSG00000085662	Homo sapiens (Human)	Flavonoids
C37	P11511	Aromatase	CYP19A1	1588	ENSG00000137869	Homo sapiens (Human)	Flavonoids
C37	P35218	Carbonic anhydrase 5A, mitochondrial	CA5A	763	ENSG00000174990	Homo sapiens (Human)	Flavonoids
C37	Q92731	Estrogen receptor beta	ESR2	2100	ENSG00000140009	Homo sapiens (Human)	Flavonoids
C37	P27338	Amine oxidase [flavin-containing] B	MAOB	4129	ENSG00000069535	Homo sapiens (Human)	Flavonoids
C37	Q9ULX7	Carbonic anhydrase 14	CA14	23632	ENSG00000118298	Homo sapiens (Human)	Flavonoids
C37	P23219	Prostaglandin G/H synthase 1	PTGS1	5742	ENSG00000095303	Homo sapiens (Human)	Flavonoids
C37	P14174	Macrophage migration inhibitory factor	MIF	4282	ENSG00000240972	Homo sapiens (Human)	Flavonoids
C37	O42713	Polyphenol oxidase 2	#N/A	#N/A	#N/A	Homo sapiens (Human)	Flavonoids
C37	P16050	Arachidonate 15-lipoxygenase	ALOX15	246	ENSG00000161905	Homo sapiens (Human)	Flavonoids
C37	P31388	5-hydroxytryptamine receptor 6	#N/A	#N/A	#N/A	Homo sapiens (Human)	Flavonoids
C37	P18901	D(1A) dopamine receptor	#N/A	#N/A	#N/A	Homo sapiens (Human)	Flavonoids
C37	O00748	Cocaine esterase	CES2	8824	ENSG00000172831	Homo sapiens (Human)	Flavonoids
C37	Q9UNQ0	ATP-binding cassette sub-family G member 2	ABCG2	9429	ENSG00000118777	Homo sapiens (Human)	Flavonoids
C37	P43166	Carbonic anhydrase 7	CA7	766	ENSG00000168748	Homo sapiens (Human)	Flavonoids
C37	P14061	Estradiol 17-beta-dehydrogenase 1	HSD17B1	3292	ENSG00000108786	Homo sapiens (Human)	Flavonoids
C37	P07943	Aldose reductase	#N/A	#N/A	#N/A	Homo sapiens (Human)	Flavonoids
C37	P22748	Carbonic anhydrase 4	CA4	762	ENSG00000167434	Homo sapiens (Human)	Flavonoids
C37	P23280	Carbonic anhydrase 6	CA6	765	ENSG00000131686	Homo sapiens (Human)	Flavonoids
C37	P37059	Estradiol 17-beta-dehydrogenase 2	HSD17B2	3294	ENSG00000086696	Homo sapiens (Human)	Flavonoids
