# Supplementary material for: Pharmacological Mechanisms Underlying the Therapeutic Effects of Danhong Injection on Cerebral Ischemia
Source: Evid Based Complement Alternat Med. 2021 May 21;2021:5584809. doi: 10.1155/2021/5584809 (PMC8163534; doi:10.1155/2021/5584809)
Supplement: Supplementary Materials — Table S1: the 37 candidate compounds of Danhong injection. Table S2: the 371 putative target proteins for the compounds. Table S3: the 413 IS-associated Homo sapiens target proteins from CTD with an inference score of ≥50. Table S4: the 61 IS-associated target proteins of Homo sapiens from Genecards with an inference score of ≥30. Table S5: degree centrality of nodes in PPI network. Table S6: betweenness centrality of nodes in the PPI network. Table S7: the GO functional enrichment analysis of diterpenoid quinones. Table S8: the KEGG pathway enrichment of diterpenoid quinones. Table S9: the KEGG pathway enrichment of DHI compounds. [file 5584809.f1.zip › 5584809.f1/S3 (2).pdf]

Table S3. The 413 IS-associated Homo sapiens target proteins from CTD with an inference score of ≥50

Gene	Disease	Inference Score
NOS2	Brain Ischemia	193.35
TNF	Brain Ischemia	171.14
FOS	Brain Ischemia	170.41
CASP3	Brain Ischemia	163.84
MAPK3	Brain Ischemia	163.34
BAX	Brain Ischemia	162.03
BCL2	Brain Ischemia	159.17
MAPK1	Brain Ischemia	156.24
IL1B	Brain Ischemia	153.26
SOD2	Brain Ischemia	147.03
CAT	Brain Ischemia	146.01
NFKBIA	Brain Ischemia	141.41
IL6	Brain Ischemia	141.29
TGFB1	Brain Ischemia	139.84
HMOX1	Brain Ischemia	135.67
NOS3	Brain Ischemia	130.87
AGT	Brain Ischemia	129.94
JUN	Cerebral Infarction	128.64
CASP9	Brain Ischemia	124.63
PTGS2	Brain Ischemia	123.85
RELA	Brain Ischemia	120.86
VEGFA	Brain Ischemia	120.76
MMP9	Brain Ischemia	120.16
GSK3B	Brain Ischemia	120.14
GFAP	Brain Ischemia	117.8
BDNF	Brain Ischemia	115.1
TP53	Brain Ischemia	113.04
CCL2	Brain Ischemia	112.3
EDN1	Brain Ischemia	112.14
AKT1	Brain Ischemia	110.88
HSPA5	Brain Ischemia	108.17
NOS1	Brain Ischemia	107.58
FGF2	Brain Ischemia	106.23
CREB1	Brain Ischemia	105.8
CCND1	Brain Ischemia	104.49
ICAM1	Brain Ischemia	104.15
NFKB1	Brain Ischemia	103.76
NFE2L2	Brain Ischemia	103.72
MPO	Brain Ischemia	103.43
TLR4	Brain Ischemia	101.36
TH	Brain Ischemia	100.45
COL1A1	Brain Ischemia	100.31
BCL2L1	Brain Ischemia	100.21
IGF1	Brain Ischemia	99.63
CTNNB1	Brain Ischemia	97.56
IFNG	Brain Ischemia	97.51
MAPK8	Brain Ischemia	97.2
APP	Brain Ischemia	96.9
SOD1	Brain Ischemia	96.19
PPARG	Brain Ischemia	95.44
CYP3A4	Cerebral Infarction	94.79
MMP2	Brain Ischemia	93.62
EGR1	Cerebral Infarction	93.49
IL10	Brain Ischemia	91.81
REN	Brain Ischemia	90.98
BAD	Brain Ischemia	90.73
CCL5	Brain Ischemia	89.95
GSR	Brain Ischemia	89.2
CXCL8	Brain Ischemia	88.53
PARP1	Brain Ischemia	88.22
DDIT3	Cerebral Infarction	88.07
CASP8	Brain Ischemia	86.28
CYBB	Brain Ischemia	85.05
NPPA	Brain Ischemia	84.94
ALB	Brain Ischemia	84.15
VCAM1	Brain Ischemia	83.95
HSPB1	Brain Ischemia	82.77
PRKCD	Brain Ischemia	82.5
PLAT	Brain Ischemia	81.67
COL3A1	Brain Ischemia	80.89
HIF1A	Brain Ischemia	80.78

FASLG	Brain Ischemia	80.44
SIRT1	Brain Ischemia	80.17
PTK2	Brain Ischemia	79.7
CCL3	Brain Ischemia	79.51
MTOR	Brain Ischemia	79.5
TRP53	Brain Ischemia	79.5
CYBA	Brain Ischemia	79.33
NGF	Brain Ischemia	78.98
TGFB2	Cerebral Infarction	78.96
CDKN1A	Brain Ischemia	78.27
SREBF1	Brain Ischemia	77.36
CCN2	Brain Ischemia	77.14
STAT3	Brain Ischemia	76.97
CYP1A2	Cerebral Infarction	76.78
FOSB	Cerebral Infarction	76.65
CEBPB	Brain Ischemia	76.02
ABCC2	Brain Ischemia	75.86
CYCS	Brain Ischemia	75.63
NPPB	Cerebral Infarction	75.48
TNFSF10	Cerebral Infarction	75.13
CXCL10	Brain Ischemia	74.71
ATF3	Cerebral Infarction	74.62
EIF4EBP1	Brain Ischemia	74.62
PPARGC1A	Brain Ischemia	74.54
SPP1	Brain Ischemia	74.41
INS1	Brain Ischemia	74.3
MMP3	Cerebral Infarction	74.13
FAS	Brain Ischemia	73.78
IL4	Cerebral Infarction	73.72
TFRC	Brain Ischemia	73.36
LEP	Brain Ischemia	73.35
NCF1	Brain Ischemia	73.34
CXCL1	Brain Ischemia	73.28
CDKN1B	Brain Ischemia	72.96
NQO1	Brain Ischemia	72.82
DUSP1	Cerebral Infarction	72.43
MYC	Brain Ischemia	72.4
MAP2K1	Brain Ischemia	72.38
ABCB1	Brain Ischemia	72.26
APOE	Brain Ischemia	72.08
NR3C1	Cerebral Infarction	72.03
SERPINE1	Cerebral Infarction	71.99
MKI67	Brain Ischemia	71.51
XDH	Brain Ischemia	71.51
JUNB	Brain Ischemia	71.47
CASP7	Brain Ischemia	71.46
CALCA	Brain Ischemia	71.35
CEBPA	Brain Ischemia	71.29
CXCL12	Brain Ischemia	71.2
CYP2E1	Brain Ischemia	70.91
BIRC5	Brain Ischemia	70.86
ABCC1	Brain Ischemia	70.79
ATP2A2	Cerebral Infarction	70.68
DNM1L	Brain Ischemia	70.54
PPP1R15A	Cerebral Infarction	70.41
TNFRSF1A	Cerebral Infarction	70.18
IL2	Brain Ischemia	69.75
LHB	Brain Ischemia	69.72
BNIP3	Brain Ischemia	69.62
SLC2A4	Brain Ischemia	69.61
GCLC	Brain Ischemia	69.42
IL1A	Brain Ischemia	69.12
GSTP1	Cerebral Infarction	68.95
GPX1	Brain Ischemia	68.81
MAOA	Cerebral Infarction	68.69
ADIPOQ	Brain Ischemia	68.32
THBS1	Cerebral Infarction	68.18
COL1A2	Brain Ischemia	67.99
FN1	Brain Ischemia	67.83
PCNA	Brain Ischemia	67.65
VIM	Brain Ischemia	67.14
MBP	Cerebral Infarction	67.13
HSPD1	Cerebral Infarction	66.82

TAC1	Brain Ischemia	66.72
RHOA	Brain Ischemia	66.66
EDNRA	Brain Ischemia	66.6
CXCL2	Cerebral Infarction	66.58
FOXO1	Brain Ischemia	66.55
PER1	Cerebral Infarction	66.37
CAV1	Brain Ischemia	66.2
ATF4	Cerebral Infarction	66.11
AIFM1	Brain Ischemia	65.76
BID	Cerebral Infarction	65.65
TIMP1	Brain Ischemia	65.63
FDFT1	Cerebral Infarction	65.56
FOSL1	Brain Ischemia	65.49
GADD45A	Cerebral Infarction	65.24
POMC	Brain Ischemia	65.16
RPS6KB1	Brain Ischemia	65.13
GJA1	Cerebral Infarction	65.1
SQSTM1	Brain Ischemia	65
BBC3	Brain Ischemia	64.94
TIMP2	Cerebral Infarction	64.71
CD44	Brain Ischemia	64.63
LAMC1	Cerebral Infarction	64.34
PDGFB	Brain Ischemia	64.23
PRKCB	Cerebral Infarction	64.19
CYP2C9	Cerebral Infarction	64.14
EGFR	Brain Ischemia	63.93
CNR1	Brain Ischemia	63.72
PPARA	Brain Ischemia	63.66
BAK1	Brain Ischemia	63.63
MYH7	Cerebral Infarction	63.59
ACTA2	Brain Ischemia	63.45
MFN2	Brain Ischemia	63.39
VCAN	Cerebral Infarction	63.09
SNCA	Brain Ischemia	63
NR1I2	Cerebral Infarction	62.81
EGR2	Cerebral Infarction	62.79
PTGS1	Brain Ischemia	62.78
MMP1	Brain Ischemia	62.76
GRIN2B	Brain Ischemia	62.71
CYP2C19	Cerebral Infarction	62.51
LPL	Brain Ischemia	62.5
XBP1	Brain Ischemia	62.45
ACE	Brain Ischemia	62.3
F3	Cerebral Infarction	62.09
AREG	Cerebral Infarction	62.04
ALDH1A1	Cerebral Infarction	62
TJP1	Brain Ischemia	61.9
RUNX2	Brain Ischemia	61.87
SRC	Brain Ischemia	61.85
ID1	Brain Ischemia	61.52
CYP2D6	Cerebral Infarction	61.39
TGFA	Cerebral Infarction	61.38
FABP4	Brain Ischemia	61.33
CDK1	Cerebral Infarction	61.3
PRDX2	Cerebral Infarction	61.2
UCP2	Brain Ischemia	61.13
IGF1R	Brain Ischemia	61.05
PPFIBP1	Cerebral Infarction	60.63
ID3	Cerebral Infarction	59.99
MAOB	Cerebral Infarction	59.9
HSP90AA1	Cerebral Infarction	59.84
STAT1	Brain Ischemia	59.78
IRS1	Brain Ischemia	59.72
DDIT4	Cerebral Infarction	59.7
MYH6	Cerebral Infarction	59.58
F2	Brain Ischemia	59.55
RB1	Brain Ischemia	59.49
RGS4	Cerebral Infarction	59.46
GSTM1	Brain Ischemia	59.38
MAPK14	Brain Ischemia	59.31
RAF1	Brain Ischemia	59.04
DRD2	Brain Ischemia	58.89
PTEN	Cerebral Infarction	58.87

AVP	Brain Ischemia	58.86
GAPDH	Brain Ischemia	58.82
SMAD3	Brain Ischemia	58.79
PSAT1	Cerebral Infarction	58.59
GPT	Brain Ischemia	58.57
HSPA8	Cerebral Infarction	58.57
AGTR1	Brain Ischemia	58.55
PRKAA1	Brain Ischemia	58.48
S100A4	Cerebral Infarction	58.4
MAFF	Cerebral Infarction	58.39
ODC1	Brain Ischemia	58.38
MAPK9	Brain Ischemia	58.34
F2R	Cerebral Infarction	58.31
OCLN	Brain Ischemia	58.3
TNFRSF11B	Brain Ischemia	58.21
TLR2	Cerebral Infarction	58.09
GCLM	Cerebral Infarction	57.93
CYP1A1	Brain Ischemia	57.86
CASP1	Brain Ischemia	57.76
ENO1	Cerebral Infarction	57.64
RELB	Cerebral Infarction	57.6
ALDOA	Cerebral Infarction	57.5
BTG2	Cerebral Infarction	57.5
SLC2A1	Brain Ischemia	57.47
ANGPTL4	Cerebral Infarction	57.42
ID2	Cerebral Infarction	57.42
RARA	Brain Ischemia	57.39
UGT1A1	Cerebral Infarction	57.37
CASP2	Cerebral Infarction	57.33
ABCA1	Cerebral Infarction	57.32
PMEPA1	Cerebral Infarction	57.27
KLF4	Cerebral Infarction	57.24
EEF1A1	Cerebral Infarction	57.17
CRP	Brain Ischemia	57.13
IKBKB	Cerebral Infarction	57.07
HGF	Brain Ischemia	57.06
CCNA2	Brain Ischemia	56.96
ADORA2A	Brain Ischemia	56.92
PIK3R1	Brain Ischemia	56.89
TXNRD1	Cerebral Infarction	56.86
ANXA4	Cerebral Infarction	56.85
RGS2	Cerebral Infarction	56.81
HSPA1A	Brain Ischemia	56.79
KLF5	Cerebral Infarction	56.75
AGTR1A	Brain Ischemia	56.71
CLU	Brain Ischemia	56.53
ATF2	Brain Ischemia	56.46
TGFBR1	Cerebral Infarction	56.44
EDNRB	Cerebral Infarction	56.4
INS	Brain Ischemia	56.25
CCNG1	Cerebral Infarction	56.23
ENO2	Brain Ischemia	56.13
TNFRSF1B	Cerebral Infarction	56.13
ACO2	Brain Ischemia	56.05
HMGB1	Cerebral Infarction	56.04
ATP1A1	Cerebral Infarction	56.03
DNAJA1	Cerebral Infarction	56.03
TRIB3	Cerebral Infarction	55.77
ACSS2	Cerebral Infarction	55.47
GEM	Cerebral Infarction	55.47
KDR	Cerebral Infarction	55.38
HDAC1	Cerebral Infarction	55.36
CYP2B6	Cerebral Infarction	55.13
CFLAR	Brain Ischemia	55.01
PRKCE	Brain Ischemia	54.96
MDM2	Brain Ischemia	54.95
FGFR1	Cerebral Infarction	54.89
CCR2	Cerebral Infarction	54.7
PRL	Brain Ischemia	54.69
IL18	Cerebral Infarction	54.68
FOXO3	Brain Ischemia	54.66
PKM	Brain Ischemia	54.66
ARC	Cerebral Infarction	54.65

G0S2	Cerebral Infarction	54.61
TNFAIP3	Cerebral Infarction	54.53
RPL6	Cerebral Infarction	54.52
GPX3	Cerebral Infarction	54.39
ELK1	Brain Ischemia	54.33
BMP4	Cerebral Infarction	54.26
PHGDH	Cerebral Infarction	54.26
RRM2	Cerebral Infarction	54.24
SLC5A3	Cerebral Infarction	54.23
EIF2S1	Brain Ischemia	54.14
PER2	Cerebral Infarction	54.08
EIF2AK3	Brain Ischemia	54.06
MAP1LC3B	Cerebral Infarction	54.03
G6PD	Brain Ischemia	54.02
PRKCA	Brain Ischemia	54.02
IGFBP4	Cerebral Infarction	53.98
ABCC3	Cerebral Infarction	53.87
SESN1	Cerebral Infarction	53.87
OAT	Cerebral Infarction	53.82
BIRC3	Brain Ischemia	53.8
CDH2	Cerebral Infarction	53.74
PDGFRA	Cerebral Infarction	53.73
EPHA4	Cerebral Infarction	53.67
SCARB1	Cerebral Infarction	53.67
EPO	Brain Ischemia	53.66
VWF	Cerebral Infarction	53.65
SP1	Brain Ischemia	53.64
MMP13	Cerebral Infarction	53.6
CASP6	Brain Ischemia	53.56
CDH1	Cerebral Infarction	53.54
MGMT	Cerebral Infarction	53.47
BMP6	Cerebral Infarction	53.44
ALDH2	Brain Ischemia	53.39
SOCS3	Brain Ischemia	53.39
RAC1	Brain Ischemia	53.37
LDHB	Cerebral Infarction	53.23
PDIA4	Cerebral Infarction	53.23
SERPINB2	Cerebral Infarction	53.23
IGFBP5	Cerebral Infarction	53.15
SGK1	Cerebral Infarction	53.06
SELE	Brain Ischemia	52.99
BECN1	Brain Ischemia	52.94
CDC25B	Cerebral Infarction	52.92
CDK2	Brain Ischemia	52.91
BGLAP	Brain Ischemia	52.82
PLA2G4A	Cerebral Infarction	52.82
UNG	Cerebral Infarction	52.82
ACTB	Cerebral Infarction	52.79
S100B	Brain Ischemia	52.72
OPA1	Brain Ischemia	52.6
ABCG1	Brain Ischemia	52.54
ABCC5	Cerebral Infarction	52.52
NT5E	Cerebral Infarction	52.52
XRCC5	Cerebral Infarction	52.47
ACHE	Brain Ischemia	52.42
BHLHE40	Cerebral Infarction	52.34
PENK	Brain Ischemia	52.33
NFE2L1	Brain Ischemia	52.29
BUB1B	Cerebral Infarction	52.23
MCL1	Cerebral Infarction	52.22
TSC22D3	Cerebral Infarction	52.19
CTSD	Cerebral Infarction	52.14
CTSK	Cerebral Infarction	52.13
SOD3	Brain Ischemia	52.12
TOP2A	Cerebral Infarction	52.12
VDAC1	Brain Ischemia	52.12
ANKRD1	Cerebral Infarction	52.1
CAMK2A	Brain Ischemia	52.1
JUP	Cerebral Infarction	52.1
WARS1	Cerebral Infarction	52.1
ASNS	Cerebral Infarction	52.08
ATM	Cerebral Infarction	52.08
CXCR4	Brain Ischemia	52.05

PBK	Cerebral Infarction	52.04
SERPINE2	Cerebral Infarction	52.04
ATF6	Cerebral Infarction	52.03
COL4A1	Brain Ischemia	52.02
NPY	Brain Ischemia	51.92
CCND3	Cerebral Infarction	51.88
CEBPD	Cerebral Infarction	51.88
RAMP2	Cerebral Infarction	51.88
PDGFRB	Brain Ischemia	51.75
BCL2L11	Brain Ischemia	51.74
CSF2	Cerebral Infarction	51.65
NR4A1	Cerebral Infarction	51.58
CD40LG	Brain Ischemia	51.56
ANXA2	Cerebral Infarction	51.5
CAPN1	Brain Ischemia	51.5
CCNE1	Brain Ischemia	51.33
HERPUD1	Cerebral Infarction	51.29
CRK	Cerebral Infarction	51.25
CKB	Brain Ischemia	51.21
HSPA1B	Cerebral Infarction	51.21
MMP14	Brain Ischemia	51.21
MAP2	Cerebral Infarction	51.2
ITGB1	Brain Ischemia	51.15
CENPF	Cerebral Infarction	51.11
CHI3L1	Cerebral Infarction	51.1
MR1	Cerebral Infarction	51.1
E2F1	Brain Ischemia	51.07
MAP2K2	Brain Ischemia	51.02
CXCL5	Cerebral Infarction	50.93
NRP1	Cerebral Infarction	50.93
MAPK10	Brain Ischemia	50.85
GRIN2A	Cerebral Infarction	50.84
MAPT	Brain Ischemia	50.79
IGFBP1	Cerebral Infarction	50.78
ABCC4	Cerebral Infarction	50.75
TNFSF11	Brain Ischemia	50.74
GSN	Cerebral Infarction	50.58
CD86	Cerebral Infarction	50.46
ERN1	Cerebral Infarction	50.43
PDK4	Cerebral Infarction	50.36
NOX4	Brain Ischemia	50.33
MCM6	Cerebral Infarction	50.32
UBE2C	Cerebral Infarction	50.32
IGFBP3	Brain Ischemia	50.3
EGF	Brain Ischemia	50.21
CYP1B1	Cerebral Infarction	50.2
RPS6	Brain Ischemia	50.17
MGLL	Cerebral Infarction	50.07
GRIA2	Brain Ischemia	50.05
FADD	Brain Ischemia	50.03
