# Supplementary material for: Pharmacological Mechanisms Underlying the Therapeutic Effects of Danhong Injection on Cerebral Ischemia
Source: Evid Based Complement Alternat Med. 2021 May 21;2021:5584809. doi: 10.1155/2021/5584809 (PMC8163534; doi:10.1155/2021/5584809)
Supplement: Supplementary Materials — Table S1: the 37 candidate compounds of Danhong injection. Table S2: the 371 putative target proteins for the compounds. Table S3: the 413 IS-associated Homo sapiens target proteins from CTD with an inference score of ≥50. Table S4: the 61 IS-associated target proteins of Homo sapiens from Genecards with an inference score of ≥30. Table S5: degree centrality of nodes in PPI network. Table S6: betweenness centrality of nodes in the PPI network. Table S7: the GO functional enrichment analysis of diterpenoid quinones. Table S8: the KEGG pathway enrichment of diterpenoid quinones. Table S9: the KEGG pathway enrichment of DHI compounds. [file 5584809.f1.zip › 5584809.f1/S4 (2).pdf]

Table S4. The 61 IS-associated target proteins of homo sapiens from Genecards with an inference score of ⩾30

Symbol	Score
BDNF	68.61
TP53	63.78
APP	62.21
MAPT	61.4
APOE	59.78
TNF	57.79
GFAP	57.32
IL6	57.09
VEGFA	56.29
SOD1	49
ACE	47.82
COL4A1	46.35
PIK3CA	45.82
IL10	44.68
MTHFR	43.74
CTNNB1	42.5
PSEN1	42.18
F2	41.44
TLR4	41.38
CD40LG	40.5
CCL2	38.9
NOS3	38.9
SLC1A2	38.83
SLC2A1	38.42
AKT1	38.14
NOTCH3	36.81
SOD2	36.79
NGF	36.2
F5	36.17
TUBB2B	35.91
IDH1	34.82
TSC2	34.46
GRIN2B	34.4
CST3	34.39
GJA1	34.03
PON1	33.93
SCN1A	33.84
EPO	33.79
ENG	33.51
MTOR	33.32
CP	33.15
NPPB	33.05
BRAF	33.04
NF1	32.16
IFNG	31.99
GRIN2A	31.88
COMT	31.84
PDGFRB	31.81
PDGFB	31.69
NOTCH1	31.57
SERPINE1	31.24
SLC6A4	31.17
CASP3	31.14

STAT3	31
MPO	30.89
HTR2A	30.66
NF2	30.4
MIR21	30.4
ELN	30.32
TLR2	30.15
TGFB1	30.14
