# Supplementary material for: Pharmacological Mechanisms Underlying the Therapeutic Effects of Danhong Injection on Cerebral Ischemia
Source: Evid Based Complement Alternat Med. 2021 May 21;2021:5584809. doi: 10.1155/2021/5584809 (PMC8163534; doi:10.1155/2021/5584809)
Supplement: Supplementary Materials — Table S1: the 37 candidate compounds of Danhong injection. Table S2: the 371 putative target proteins for the compounds. Table S3: the 413 IS-associated Homo sapiens target proteins from CTD with an inference score of ≥50. Table S4: the 61 IS-associated target proteins of Homo sapiens from Genecards with an inference score of ≥30. Table S5: degree centrality of nodes in PPI network. Table S6: betweenness centrality of nodes in the PPI network. Table S7: the GO functional enrichment analysis of diterpenoid quinones. Table S8: the KEGG pathway enrichment of diterpenoid quinones. Table S9: the KEGG pathway enrichment of DHI compounds. [file 5584809.f1.zip › 5584809.f1/S5 (2).pdf]

Table S5. Degree centrality of nodes in PPI network

1	HSP90AA1	Degree: 811.0
2	HSP90AB1	Degree: 566.0
3	NTRK1	Degree: 306.0
4	TP53	Degree: 265.0
5	ESR1	Degree: 243.0
6	APP	Degree: 224.0
7	CUL3	Degree: 215.0
8	NR3C1	Degree: 212.0
9	EGFR	Degree: 203.0
10	HSPA8	Degree: 199.0
11	XPO1	Degree: 195.0
12	COPS5	Degree: 195.0
13	YWHAZ	Degree: 180.0
14	MCM2	Degree: 169.0
15	CDC37	Degree: 166.0
16	AR	Degree: 166.0
17	CDK2	Degree: 165.0
18	HSPA4	Degree: 163.0
19	CUL1	Degree: 161.0
20	EP300	Degree: 159.0
21	HSPA5	Degree: 153.0
22	VCP	Degree: 150.0
23	NPM1	Degree: 149.0
24	FN1	Degree: 147.0
25	BRCA1	Degree: 142.0
26	GRB2	Degree: 140.0
27	IKBKG	Degree: 139.0
28	TRAF6	Degree: 138.0
29	MYC	Degree: 136.0
30	HDAC5	Degree: 136.0
31	STUB1	Degree: 136.0
32	TUBB	Degree: 135.0
33	RARA	Degree: 134.0
34	HDAC1	Degree: 134.0
35	MDM2	Degree: 133.0
36	YWHAQ	Degree: 132.0
37	UBE2I	Degree: 131.0
38	HUWE1	Degree: 128.0
39	AKT1	Degree: 128.0
40	CREBBP	Degree: 126.0
41	RNF2	Degree: 125.0
42	SRC	Degree: 124.0
43	YWHAG	Degree: 124.0
44	YWHAE	Degree: 121.0
45	HSPA1A	Degree: 120.0
46	HSPA1B	Degree: 120.0
47	CAND1	Degree: 118.0
48	EEF1A1	Degree: 116.0
49	HSPB1	Degree: 115.0
50	HNRNPA1	Degree: 115.0
51	RELA	Degree: 114.0
52	HNRNPU	Degree: 112.0

53	SNW1	Degree: 111.0
54	PARK2	Degree: 111.0
55	YWHAB	Degree: 109.0
56	MAPK1	Degree: 108.0
57	CDC5L	Degree: 106.0
58	JUN	Degree: 105.0
59	HSPA9	Degree: 105.0
60	SMAD3	Degree: 104.0
61	NFKB1	Degree: 104.0
62	RPS27A	Degree: 102.0
63	VHL	Degree: 102.0
64	FBXO6	Degree: 101.0
65	PRKDC	Degree: 101.0
66	CUL2	Degree: 101.0
67	CDKN1A	Degree: 101.0
68	VCAM1	Degree: 100.0
69	GAPDH	Degree: 99.0
70	RB1	Degree: 97.0
71	PML	Degree: 97.0
72	CUL5	Degree: 97.0
73	FUS	Degree: 95.0
74	HSPD1	Degree: 95.0
75	STAT3	Degree: 93.0
76	NCL	Degree: 92.0
77	GSK3B	Degree: 92.0
78	CLTC	Degree: 92.0
79	CDK1	Degree: 92.0
80	SHC1	Degree: 91.0
81	SP1	Degree: 91.0
82	PARP1	Degree: 91.0
83	RPS3	Degree: 90.0
84	TRAF2	Degree: 90.0
85	FKBP5	Degree: 89.0
86	TRIM28	Degree: 89.0
87	U2AF2	Degree: 88.0
88	MYH9	Degree: 86.0
89	MAP3K3	Degree: 86.0
90	RACK1	Degree: 86.0
91	PPARG	Degree: 86.0
92	VIM	Degree: 86.0
93	CUL4B	Degree: 86.0
94	RAF1	Degree: 85.0
95	IKBKB	Degree: 84.0
96	ABL1	Degree: 84.0
97	HSPA1L	Degree: 84.0
98	RXRA	Degree: 83.0
99	STIP1	Degree: 83.0
100	AURKA	Degree: 82.0
101	DNAJA1	Degree: 82.0
102	UBL4A	Degree: 82.0
103	HDAC2	Degree: 82.0
104	IKBKE	Degree: 81.0
105	MAP3K1	Degree: 81.0
106	TUBA1A	Degree: 81.0

107	CCT2	Degree: 81.0
108	MAPK3	Degree: 81.0
109	CHUK	Degree: 80.0
110	SKP1	Degree: 80.0
111	TARDBP	Degree: 80.0
112	ARRB1	Degree: 80.0
113	HDAC6	Degree: 79.0
114	BAG3	Degree: 78.0
115	HDAC3	Degree: 78.0
116	EEF2	Degree: 77.0
117	PKM	Degree: 77.0
118	CASP3	Degree: 76.0
119	SMARCA4	Degree: 76.0
120	RUVBL1	Degree: 75.0
121	RPS3A	Degree: 75.0
122	RPS4X	Degree: 75.0
123	RPS16	Degree: 75.0
124	PAN2	Degree: 75.0
125	TUBB4B	Degree: 75.0
126	SQSTM1	Degree: 75.0
127	BTRC	Degree: 75.0
128	LRRK2	Degree: 74.0
129	COPS6	Degree: 74.0
130	PCNA	Degree: 74.0
131	CSNK2B	Degree: 73.0
132	NEDD8	Degree: 73.0
133	USP7	Degree: 73.0
134	UBB	Degree: 73.0
135	HIF1A	Degree: 73.0
136	MAPK14	Degree: 72.0
137	FBXW11	Degree: 72.0
138	SNCA	Degree: 72.0
139	EZH2	Degree: 72.0
140	PTEN	Degree: 72.0
141	CUL4A	Degree: 72.0
142	MCM5	Degree: 71.0
143	BAG2	Degree: 71.0
144	TCP1	Degree: 71.0
145	SUZ12	Degree: 71.0
146	TERF1	Degree: 71.0
147	PSMA3	Degree: 71.0
148	TNFRSF1A	Degree: 70.0
149	MAPK8	Degree: 70.0
150	RPS6	Degree: 69.0
151	CASP8	Degree: 69.0
152	SFPQ	Degree: 69.0
153	SIRT1	Degree: 69.0
154	PPP2R1A	Degree: 69.0
155	YWHAH	Degree: 69.0
156	ATF2	Degree: 68.0
157	NCOR1	Degree: 68.0
158	FYN	Degree: 68.0
159	DCUN1D1	Degree: 68.0
160	HIST3H3	Degree: 68.0

161	RPLP0	Degree: 68.0
162	RPS6KB2	Degree: 67.0
163	FLNA	Degree: 67.0
164	TUBG1	Degree: 67.0
165	CDKN2A	Degree: 67.0
166	RPL7	Degree: 66.0
167	ATP5A1	Degree: 66.0
168	NCOR2	Degree: 65.0
169	PIN1	Degree: 65.0
170	RPL5	Degree: 65.0
171	SFN	Degree: 64.0
172	NCOA3	Degree: 64.0
173	H2AFX	Degree: 64.0
174	CDK9	Degree: 64.0
175	HNRNPR	Degree: 63.0
176	RPS15A	Degree: 62.0
177	NCOA1	Degree: 61.0
178	BCL2	Degree: 61.0
179	IRAK1	Degree: 61.0
180	NUDCD3	Degree: 61.0
181	SIN3A	Degree: 61.0
182	SUMO1	Degree: 61.0
183	SLC25A5	Degree: 61.0
184	PSMC5	Degree: 61.0
185	ACTG1	Degree: 61.0
186	PTGES3	Degree: 61.0
187	RPSA	Degree: 60.0
188	SNRNP200	Degree: 59.0
189	CAV1	Degree: 59.0
190	KAT5	Degree: 59.0
191	TUFM	Degree: 59.0
192	PRKCA	Degree: 59.0
193	XIAP	Degree: 59.0
194	RPL3	Degree: 59.0
195	RPL23A	Degree: 59.0
196	MCL1	Degree: 58.0
197	IRS4	Degree: 58.0
198	MAP3K14	Degree: 57.0
199	CRY2	Degree: 57.0
200	TAB2	Degree: 57.0
201	SUMO2	Degree: 57.0
202	MAP3K7	Degree: 57.0
203	TRAF1	Degree: 57.0
204	PRKACA	Degree: 57.0
205	PRKCD	Degree: 57.0
206	CDK4	Degree: 57.0
207	KPNA2	Degree: 56.0
208	ESR2	Degree: 56.0
209	NFKB2	Degree: 56.0
210	CSNK2A2	Degree: 55.0
211	LYN	Degree: 55.0
212	AURKB	Degree: 55.0
213	SPTAN1	Degree: 55.0
214	PRMT5	Degree: 55.0

215	PRKCZ	Degree: 55.0
216	AHSA1	Degree: 55.0
217	KAT2B	Degree: 55.0
218	SKI	Degree: 54.0
219	HDAC4	Degree: 54.0
220	FBXO25	Degree: 54.0
221	PCBP1	Degree: 54.0
222	ATP5B	Degree: 54.0
223	CEP250	Degree: 53.0
224	NR4A1	Degree: 53.0
225	RPS20	Degree: 52.0
226	CASP9	Degree: 52.0
227	ERBB2	Degree: 51.0
228	NOS2	Degree: 51.0
229	SF3B3	Degree: 50.0
230	MAP3K5	Degree: 50.0
231	CACYBP	Degree: 50.0
232	ILK	Degree: 49.0
233	CANX	Degree: 49.0
234	E2F1	Degree: 49.0
235	TBK1	Degree: 49.0
236	NEDD4L	Degree: 49.0
237	EFTUD2	Degree: 49.0
238	ISG15	Degree: 49.0
239	LGALS3BP	Degree: 49.0
240	CEBPB	Degree: 48.0
241	TGFBR1	Degree: 48.0
242	ETS1	Degree: 48.0
243	MAP2K1	Degree: 48.0
244	PHB2	Degree: 48.0
245	CREB1	Degree: 47.0
246	SKP2	Degree: 47.0
247	SMARCC1	Degree: 47.0
248	DAXX	Degree: 47.0
249	TBP	Degree: 47.0
250	DYNC1H1	Degree: 47.0
251	SF3A1	Degree: 47.0
252	FBXW7	Degree: 47.0
253	PGR	Degree: 47.0
254	CDH1	Degree: 47.0
255	RIPK1	Degree: 46.0
256	EPRS	Degree: 46.0
257	HSPA2	Degree: 46.0
258	GAN	Degree: 46.0
259	NCOA6	Degree: 45.0
260	ZBTB16	Degree: 45.0
261	WWOX	Degree: 45.0
262	VDAC1	Degree: 45.0
263	PRPF19	Degree: 45.0
264	DNAJA2	Degree: 45.0
265	CEBPA	Degree: 44.0
266	CFTR	Degree: 44.0
267	BAX	Degree: 44.0
268	SET	Degree: 44.0

269	TERF2	Degree: 44.0
270	NFE2L2	Degree: 44.0
271	TAB1	Degree: 44.0
272	UBE3A	Degree: 44.0
273	HSF1	Degree: 44.0
274	HSPA6	Degree: 44.0
275	CALR	Degree: 43.0
276	JAK2	Degree: 43.0
277	SMARCA2	Degree: 43.0
278	FASN	Degree: 43.0
279	UBE2N	Degree: 43.0
280	PTK2	Degree: 43.0
281	PRPF8	Degree: 43.0
282	USP9X	Degree: 43.0
283	RAD21	Degree: 42.0
284	SMARCB1	Degree: 42.0
285	THRB	Degree: 42.0
286	NFKBIB	Degree: 42.0
287	DNAJC7	Degree: 42.0
288	GRK5	Degree: 42.0
289	BTK	Degree: 42.0
290	ACTN4	Degree: 42.0
291	NUDC	Degree: 42.0
292	RPL17	Degree: 42.0
293	RAN	Degree: 41.0
294	CHEK1	Degree: 41.0
295	SRPK1	Degree: 41.0
296	FKBP4	Degree: 41.0
297	NCOA2	Degree: 41.0
298	TXN	Degree: 41.0
299	ALDOA	Degree: 41.0
300	P4HB	Degree: 41.0
301	ACTN1	Degree: 41.0
302	HMGB1	Degree: 41.0
303	CDC25B	Degree: 41.0
304	HMGA1	Degree: 41.0
305	HNF4A	Degree: 41.0
306	RIPK3	Degree: 40.0
307	HNRNPL	Degree: 40.0
308	BAD	Degree: 40.0
309	MCM3	Degree: 40.0
310	STK11	Degree: 40.0
311	CASP7	Degree: 40.0
312	LDHA	Degree: 40.0
313	ERBB3	Degree: 40.0
314	KIAA1549	Degree: 40.0
315	TPM3	Degree: 40.0
316	THRAP3	Degree: 40.0
317	MED1	Degree: 40.0
318	BIRC2	Degree: 40.0
319	BIRC3	Degree: 40.0
320	PPARGC1A	Degree: 40.0
321	PSMD1	Degree: 40.0
322	ARAF	Degree: 40.0

323	BCL2L1	Degree: 39.0
324	SSB	Degree: 39.0
325	RANBP9	Degree: 39.0
326	UBE2L3	Degree: 39.0
327	BCL6	Degree: 39.0
328	PAK1	Degree: 39.0
329	PGK1	Degree: 39.0
330	CDK5	Degree: 39.0
331	RBM14-RBM4	Degree: 38.0
332	SMARCE1	Degree: 38.0
333	LCK	Degree: 38.0
334	TP53BP1	Degree: 38.0
335	RBM14	Degree: 38.0
336	BRAF	Degree: 38.0
337	QARS	Degree: 37.0
338	DNAJA3	Degree: 37.0
339	MET	Degree: 37.0
340	TNFAIP3	Degree: 37.0
341	HSPH1	Degree: 37.0
342	RIPK2	Degree: 36.0
343	JAK1	Degree: 36.0
344	MYOD1	Degree: 36.0
345	EPAS1	Degree: 36.0
346	TUBB2B	Degree: 36.0
347	PRKAB1	Degree: 36.0
348	SUGT1	Degree: 36.0
349	BCR	Degree: 36.0
350	GNB2	Degree: 36.0
351	MAX	Degree: 35.0
352	STAT5A	Degree: 35.0
353	UBA1	Degree: 35.0
354	ESRRB	Degree: 35.0
355	PPP5C	Degree: 35.0
356	PRKCE	Degree: 35.0
357	FAS	Degree: 35.0
358	HCFC1	Degree: 35.0
359	RPL32	Degree: 35.0
360	AGO2	Degree: 34.0
361	RUNX1	Degree: 34.0
362	DSP	Degree: 34.0
363	COPS2	Degree: 34.0
364	CBX5	Degree: 34.0
365	SRF	Degree: 34.0
366	KMT2A	Degree: 34.0
367	TSG101	Degree: 34.0
368	PRKAA1	Degree: 34.0
369	MAPK7	Degree: 34.0
370	AMBRA1	Degree: 33.0
371	MARS	Degree: 33.0
372	SSBP1	Degree: 33.0
373	TADA2A	Degree: 33.0
374	IQCB1	Degree: 33.0
375	NR0B2	Degree: 33.0
376	NRIP1	Degree: 33.0

377	SGK1	Degree: 32.0
378	CSNK1A1	Degree: 32.0
379	TRADD	Degree: 32.0
380	SUV39H1	Degree: 32.0
381	MDM4	Degree: 32.0
382	EIF4B	Degree: 32.0
383	FOXO1	Degree: 32.0
384	NUMA1	Degree: 32.0
385	SENP3	Degree: 32.0
386	PPARA	Degree: 32.0
387	PPID	Degree: 32.0
388	TNK2	Degree: 32.0
389	MAPK9	Degree: 32.0
390	EIF2AK2	Degree: 32.0
391	MAGED2	Degree: 32.0
392	BCL10	Degree: 32.0
393	CDK6	Degree: 32.0
394	RPAP3	Degree: 31.0
395	BAG1	Degree: 31.0
396	MGMT	Degree: 31.0
397	KEAP1	Degree: 31.0
398	SETDB1	Degree: 31.0
399	PRPF6	Degree: 31.0
400	UBASH3B	Degree: 31.0
401	UQCRC2	Degree: 31.0
402	PRKCB	Degree: 31.0
403	MAPK6	Degree: 31.0
404	USP19	Degree: 31.0
405	KLF5	Degree: 31.0
406	CLU	Degree: 31.0
407	ATF3	Degree: 31.0
408	IGF1R	Degree: 30.0
409	SIAH1	Degree: 30.0
410	CEP76	Degree: 30.0
411	SRSF5	Degree: 30.0
412	MTA2	Degree: 30.0
413	DARS	Degree: 30.0
414	NDRG1	Degree: 30.0
415	PPARD	Degree: 30.0
416	MMS19	Degree: 30.0
417	HSPE1	Degree: 30.0
418	COPS3	Degree: 29.0
419	BECN1	Degree: 29.0
420	CEP57	Degree: 29.0
421	TERT	Degree: 29.0
422	TOP2B	Degree: 29.0
423	UCHL1	Degree: 29.0
424	POU2F1	Degree: 29.0
425	GNB1	Degree: 29.0
426	RBCK1	Degree: 29.0
427	CFLAR	Degree: 29.0
428	CDK13	Degree: 28.0
429	RPS6KB1	Degree: 28.0
430	IRF3	Degree: 28.0

431	SMARCD1	Degree: 28.0
432	MTHFD1	Degree: 28.0
433	AGO3	Degree: 28.0
434	GADD45A	Degree: 28.0
435	SVIL	Degree: 28.0
436	TGFBR2	Degree: 28.0
437	FKBPL	Degree: 28.0
438	NFKBIE	Degree: 28.0
439	LNX1	Degree: 28.0
440	BCL2L11	Degree: 28.0
441	PRKCI	Degree: 28.0
442	YES1	Degree: 28.0
443	ATR	Degree: 28.0
444	CDK3	Degree: 28.0
445	CDK7	Degree: 28.0
446	RPS6KA1	Degree: 27.0
447	UBQLN1	Degree: 27.0
448	SSR4	Degree: 27.0
449	BAG4	Degree: 27.0
450	SUPT5H	Degree: 27.0
451	PRDX6	Degree: 27.0
452	FOXM1	Degree: 27.0
453	GRK2	Degree: 27.0
454	ALK	Degree: 27.0
455	MAP2K7	Degree: 27.0
456	PSMB5	Degree: 27.0
457	BID	Degree: 27.0
458	PDGFRB	Degree: 27.0
459	NR1I2	Degree: 27.0
460	CDC25C	Degree: 27.0
461	PIAS2	Degree: 26.0
462	RAD51	Degree: 26.0
463	LRIF1	Degree: 26.0
464	CAMK2A	Degree: 26.0
465	EPB41L3	Degree: 26.0
466	SREBF1	Degree: 26.0
467	NR1H3	Degree: 26.0
468	FANCA	Degree: 26.0
469	PINK1	Degree: 26.0
470	NR1H2	Degree: 26.0
471	NEK8	Degree: 26.0
472	MAP3K8	Degree: 26.0
473	NPHP4	Degree: 26.0
474	HSP90AA5P	Degree: 26.0
475	AIP	Degree: 25.0
476	RFC4	Degree: 25.0
477	SNRPE	Degree: 25.0
478	NR3C2	Degree: 25.0
479	POLR2E	Degree: 25.0
480	GSK3A	Degree: 25.0
481	PRKD1	Degree: 25.0
482	GATA2	Degree: 25.0
483	BIRC5	Degree: 25.0
484	HRAS	Degree: 25.0

485	TRIM24	Degree: 25.0
486	RPL29	Degree: 25.0
487	SGTA	Degree: 24.0
488	IRF1	Degree: 24.0
489	STAT5B	Degree: 24.0
490	CD4	Degree: 24.0
491	RPS6KA5	Degree: 24.0
492	MED14	Degree: 24.0
493	SPI1	Degree: 24.0
494	CD2AP	Degree: 24.0
495	FKBP8	Degree: 24.0
496	TYK2	Degree: 24.0
497	AKT2	Degree: 24.0
498	BLM	Degree: 24.0
499	CCDC6	Degree: 24.0
500	BRCC3	Degree: 24.0
501	SUMO4	Degree: 24.0
502	RPS6KA3	Degree: 23.0
503	TRIM32	Degree: 23.0
504	MAGEA11	Degree: 23.0
505	UHRF2	Degree: 23.0
506	MAPKAPK2	Degree: 23.0
507	DAPK1	Degree: 23.0
508	DDOST	Degree: 23.0
509	SKIV2L2	Degree: 23.0
510	NUDCD2	Degree: 23.0
511	NR2C2	Degree: 23.0
512	PTK2B	Degree: 23.0
513	GIGYF2	Degree: 23.0
514	VARS	Degree: 23.0
515	AHR	Degree: 23.0
516	MAP3K2	Degree: 23.0
517	PELP1	Degree: 23.0
518	HMGB2	Degree: 23.0
519	EIF3H	Degree: 22.0
520	KDR	Degree: 22.0
521	SMARCD3	Degree: 22.0
522	TTC1	Degree: 22.0
523	DAP3	Degree: 22.0
524	TRAF3IP1	Degree: 22.0
525	GNAI2	Degree: 22.0
526	TBL1XR1	Degree: 22.0
527	HSPA7	Degree: 22.0
528	CDK11B	Degree: 22.0
529	RXRB	Degree: 21.0
530	ST13	Degree: 21.0
531	KRT31	Degree: 21.0
532	TRIP4	Degree: 21.0
533	EEF1AKMT3	Degree: 21.0
534	NR1I3	Degree: 21.0
535	ZBTB17	Degree: 21.0
536	PRKCQ	Degree: 21.0
537	PKN1	Degree: 21.0
538	DYRK2	Degree: 21.0

539	CDK18	Degree: 21.0
540	HCK	Degree: 21.0
541	UNC45A	Degree: 20.0
542	WWP1	Degree: 20.0
543	CRYAB	Degree: 20.0
544	AGO4	Degree: 20.0
545	BRMS1	Degree: 20.0
546	DNMT3L	Degree: 20.0
547	MAP3K11	Degree: 20.0
548	ERBB4	Degree: 20.0
549	FANCC	Degree: 20.0
550	ANLN	Degree: 20.0
551	LARP7	Degree: 20.0
552	PRKAA2	Degree: 20.0
553	MBD3	Degree: 20.0
554	CDC73	Degree: 20.0
555	CHORDC1	Degree: 20.0
556	LMAN1	Degree: 19.0
557	SNRNP40	Degree: 19.0
558	CASP10	Degree: 19.0
559	KCNH2	Degree: 19.0
560	CCND3	Degree: 19.0
561	DYNLT1	Degree: 19.0
562	TDG	Degree: 19.0
563	HP1BP3	Degree: 19.0
564	FGFR1	Degree: 19.0
565	TOMM40	Degree: 19.0
566	PKN2	Degree: 19.0
567	SLC25A4	Degree: 19.0
568	ZAP70	Degree: 19.0
569	GADD45G	Degree: 19.0
570	CLK3	Degree: 19.0
571	TNIP1	Degree: 19.0
572	ARID1A	Degree: 19.0
573	LIMK1	Degree: 18.0
574	RXRG	Degree: 18.0
575	STAT2	Degree: 18.0
576	CAPN2	Degree: 18.0
577	DYRK1B	Degree: 18.0
578	FES	Degree: 18.0
579	MOS	Degree: 18.0
580	AGO1	Degree: 18.0
581	MED6	Degree: 18.0
582	FLII	Degree: 18.0
583	FBXW8	Degree: 18.0
584	PPP3CA	Degree: 18.0
585	BMX	Degree: 18.0
586	HES1	Degree: 18.0
587	ACP1	Degree: 18.0
588	PPP6R3	Degree: 18.0
589	CENPB	Degree: 17.0
590	URI1	Degree: 17.0
591	TJP2	Degree: 17.0
592	CNOT1	Degree: 17.0

593	CAMK2D	Degree: 17.0
594	ITK	Degree: 17.0
595	DIABLO	Degree: 17.0
596	SIM2	Degree: 17.0
597	DAPK3	Degree: 17.0
598	MRTO4	Degree: 17.0
599	MIF	Degree: 17.0
600	EIF4A2	Degree: 17.0
601	TJP1	Degree: 17.0
602	MST1R	Degree: 17.0
603	ETS2	Degree: 17.0
604	FHL3	Degree: 17.0
605	PIH1D1	Degree: 17.0
606	MAP2K2	Degree: 17.0
607	FTH1	Degree: 17.0
608	CDK11A	Degree: 17.0
609	PSMD10	Degree: 17.0
610	ARNTL	Degree: 17.0
611	CDK5R1	Degree: 17.0
612	RAD52	Degree: 16.0
613	CKS1B	Degree: 16.0
614	MAP4K1	Degree: 16.0
615	RPS6KA2	Degree: 16.0
616	CRNKL1	Degree: 16.0
617	MAGEB2	Degree: 16.0
618	MBD1	Degree: 16.0
619	EGLN1	Degree: 16.0
620	MAST2	Degree: 16.0
621	RUNX1T1	Degree: 16.0
622	KCNA5	Degree: 16.0
623	SOCS6	Degree: 16.0
624	GRIP1	Degree: 16.0
625	MED25	Degree: 16.0
626	USP49	Degree: 16.0
627	EIF5B	Degree: 16.0
628	SCRIB	Degree: 16.0
629	TPT1	Degree: 16.0
630	CYCS	Degree: 16.0
631	NOD1	Degree: 16.0
632	FGR	Degree: 16.0
633	FBXW2	Degree: 16.0
634	ASB2	Degree: 16.0
635	MAPK10	Degree: 16.0
636	BLK	Degree: 16.0
637	PTGS2	Degree: 16.0
638	FKBP6	Degree: 16.0
639	PAFAH1B1	Degree: 16.0
640	ANP32A	Degree: 16.0
641	ATP1B1	Degree: 16.0
642	PRPF4B	Degree: 16.0
643	AP3D1	Degree: 16.0
644	FGFR1OP	Degree: 15.0
645	WDR6	Degree: 15.0
646	ZHX1	Degree: 15.0

647	MARCKS	Degree: 15.0
648	BAK1	Degree: 15.0
649	CALD1	Degree: 15.0
650	IRF2	Degree: 15.0
651	PASK	Degree: 15.0
652	HSP90AB2P	Degree: 15.0
653	ERN1	Degree: 15.0
654	NOS3	Degree: 15.0
655	ASB15	Degree: 15.0
656	CAP1	Degree: 15.0
657	PIK3C3	Degree: 15.0
658	PIM1	Degree: 15.0
659	PCGF1	Degree: 15.0
660	AARS	Degree: 15.0
661	PRKAB2	Degree: 15.0
662	PRKAG1	Degree: 15.0
663	XRCC1	Degree: 15.0
664	NEK9	Degree: 15.0
665	TOMM34	Degree: 15.0
666	STK38	Degree: 15.0
667	SAP130	Degree: 15.0
668	CDK14	Degree: 15.0
669	WASL	Degree: 15.0
670	RAG1	Degree: 14.0
671	RET	Degree: 14.0
672	CDC37L1	Degree: 14.0
673	LRP1	Degree: 14.0
674	AXL	Degree: 14.0
675	SPEN	Degree: 14.0
676	WDR76	Degree: 14.0
677	CAMK2G	Degree: 14.0
678	AURKC	Degree: 14.0
679	ANAPC2	Degree: 14.0
680	MID1	Degree: 14.0
681	NR2F2	Degree: 14.0
682	EPHA2	Degree: 14.0
683	RNF40	Degree: 14.0
684	TTC4	Degree: 14.0
685	TADA3	Degree: 14.0
686	GAPVD1	Degree: 14.0
687	DDX54	Degree: 14.0
688	RNF111	Degree: 14.0
689	GNA12	Degree: 14.0
690	HSP90AB3P	Degree: 14.0
691	MGEA5	Degree: 14.0
692	LRSAM1	Degree: 14.0
693	KSR1	Degree: 14.0
694	HK2	Degree: 14.0
695	CEBPE	Degree: 13.0
696	WSB2	Degree: 13.0
697	ASXL1	Degree: 13.0
698	MAP4K4	Degree: 13.0
699	DSN1	Degree: 13.0
700	TRIM37	Degree: 13.0

701	MDH1	Degree: 13.0
702	PSAT1	Degree: 13.0
703	TGFB1I1	Degree: 13.0
704	NKX2-1	Degree: 13.0
705	TOMM70	Degree: 13.0
706	FGFR3	Degree: 13.0
707	FOXL1	Degree: 13.0
708	PACRG	Degree: 13.0
709	AJUBA	Degree: 13.0
710	KSR2	Degree: 13.0
711	PTK6	Degree: 13.0
712	CLK2	Degree: 13.0
713	MLF2	Degree: 13.0
714	HSD17B4	Degree: 13.0
715	TRAF3IP2	Degree: 13.0
716	TRIM41	Degree: 13.0
717	GZMB	Degree: 13.0
718	NLRP12	Degree: 13.0
719	RABEP2	Degree: 12.0
720	PRRC2C	Degree: 12.0
721	CARD8	Degree: 12.0
722	TNK1	Degree: 12.0
723	TXNDC5	Degree: 12.0
724	MATK	Degree: 12.0
725	SSR3	Degree: 12.0
726	IRAK2	Degree: 12.0
727	CAMK2B	Degree: 12.0
728	CCNH	Degree: 12.0
729	BIRC6	Degree: 12.0
730	RCAN1	Degree: 12.0
731	CLOCK	Degree: 12.0
732	KLHL38	Degree: 12.0
733	PRAME	Degree: 12.0
734	EPB41L2	Degree: 12.0
735	RNF10	Degree: 12.0
736	SRA1	Degree: 12.0
737	NANS	Degree: 12.0
738	NTRK3	Degree: 12.0
739	CDK15	Degree: 12.0
740	PRKACB	Degree: 12.0
741	PRKCG	Degree: 12.0
742	MAP2K5	Degree: 12.0
743	DET1	Degree: 12.0
744	APAF1	Degree: 12.0
745	APOB	Degree: 12.0
746	RFWD3	Degree: 12.0
747	TTC5	Degree: 12.0
748	PRDM1	Degree: 12.0
749	NCOA4	Degree: 12.0
750	PBX1	Degree: 12.0
751	CAMKK2	Degree: 12.0
752	NES	Degree: 12.0
753	ECD	Degree: 12.0
754	RAD9A	Degree: 11.0

755	LARP4B	Degree: 11.0
756	LIMK2	Degree: 11.0
757	PAK6	Degree: 11.0
758	KLHL15	Degree: 11.0
759	PSMC3IP	Degree: 11.0
760	ASB3	Degree: 11.0
761	PRKD2	Degree: 11.0
762	SMYD2	Degree: 11.0
763	TFDP3	Degree: 11.0
764	FER	Degree: 11.0
765	PRAM1	Degree: 11.0
766	EPHA4	Degree: 11.0
767	CAMKK1	Degree: 11.0
768	SCO2	Degree: 11.0
769	NPAS2	Degree: 11.0
770	FBXW5	Degree: 11.0
771	NTRK2	Degree: 11.0
772	GPR75-ASB3	Degree: 11.0
773	BUB1B-PAK6	Degree: 11.0
774	POU1F1	Degree: 11.0
775	POU2F2	Degree: 11.0
776	FBXO24	Degree: 11.0
777	FBXO3	Degree: 11.0
778	NR0B1	Degree: 11.0
779	ASB6	Degree: 11.0
780	GBA	Degree: 11.0
781	RALBP1	Degree: 11.0
782	NSD1	Degree: 11.0
783	BTG1	Degree: 11.0
784	FBXL12	Degree: 11.0
785	KLHL13	Degree: 11.0
786	BIRC7	Degree: 11.0
787	FBXL18	Degree: 11.0
788	RNF114	Degree: 10.0
789	KATNA1	Degree: 10.0
790	KDM5A	Degree: 10.0
791	OSGEP	Degree: 10.0
792	CKS2	Degree: 10.0
793	NLRP2	Degree: 10.0
794	IARS2	Degree: 10.0
795	DCAF6	Degree: 10.0
796	NLRP3	Degree: 10.0
797	HDAC8	Degree: 10.0
798	MAFG	Degree: 10.0
799	FBXO17	Degree: 10.0
800	PHF8	Degree: 10.0
801	RAD54L2	Degree: 10.0
802	ATG12	Degree: 10.0
803	SLC2A4	Degree: 10.0
804	FAM83H	Degree: 10.0
805	TSSK6	Degree: 10.0
806	KBTBD7	Degree: 10.0
807	TXN2	Degree: 10.0
808	DCC	Degree: 10.0

809	SYT1	Degree: 10.0
810	NSL1	Degree: 10.0
811	ASB17	Degree: 10.0
812	MAFF	Degree: 10.0
813	SRPK3	Degree: 10.0
814	NR2E3	Degree: 10.0
815	TTC3	Degree: 10.0
816	YTHDC2	Degree: 10.0
817	KLHL22	Degree: 10.0
818	TYRO3	Degree: 10.0
819	MIS12	Degree: 10.0
820	PRKCH	Degree: 10.0
821	MYLK2	Degree: 10.0
822	WDR20	Degree: 10.0
823	GNAQ	Degree: 10.0
824	SMYD3	Degree: 10.0
825	ACVR1B	Degree: 10.0
826	HIP1	Degree: 10.0
827	ICK	Degree: 10.0
828	KLHL26	Degree: 10.0
829	HMOX2	Degree: 10.0
830	LIMD1	Degree: 10.0
831	FNIP1	Degree: 9.0
832	IDE	Degree: 9.0
833	CSF1R	Degree: 9.0
834	FBXO28	Degree: 9.0
835	RHOBTB2	Degree: 9.0
836	NRBF2	Degree: 9.0
837	FOXJ2	Degree: 9.0
838	EIF2AK3	Degree: 9.0
839	STK38L	Degree: 9.0
840	SMG1	Degree: 9.0
841	CAMK4	Degree: 9.0
842	TNKS2	Degree: 9.0
843	TSSK1B	Degree: 9.0
844	TRIM17	Degree: 9.0
845	FBXL2	Degree: 9.0
846	PCGF6	Degree: 9.0
847	CHTF18	Degree: 9.0
848	NR2F6	Degree: 9.0
849	NFRKB	Degree: 9.0
850	NUCB1	Degree: 9.0
851	HERC4	Degree: 9.0
852	WNK4	Degree: 9.0
853	FBXO18	Degree: 9.0
854	ARPC5	Degree: 9.0
855	POLH	Degree: 9.0
856	FBXO4	Degree: 9.0
857	C12orf10	Degree: 9.0
858	MAPK15	Degree: 9.0
859	RPS6KA6	Degree: 9.0
860	BMPR1A	Degree: 9.0
861	TEAD2	Degree: 9.0
862	METTL18	Degree: 9.0

863	ENC1	Degree: 9.0
864	USP50	Degree: 9.0
865	CEP97	Degree: 9.0
866	RHOBTB3	Degree: 9.0
867	MYOCD	Degree: 9.0
868	RNF34	Degree: 9.0
869	PIM2	Degree: 8.0
870	PNRC2	Degree: 8.0
871	CAMKMT	Degree: 8.0
872	ZBTB3	Degree: 8.0
873	TP53RK	Degree: 8.0
874	TNKS	Degree: 8.0
875	CLIP1	Degree: 8.0
876	EDNRA	Degree: 8.0
877	KTN1	Degree: 8.0
878	FOXD4L6	Degree: 8.0
879	FAM103A1	Degree: 8.0
880	CXXC1	Degree: 8.0
881	EPHB1	Degree: 8.0
882	AKT3	Degree: 8.0
883	NOS1	Degree: 8.0
884	DDR2	Degree: 8.0
885	CWC15	Degree: 8.0
886	ZNF496	Degree: 8.0
887	EDRF1	Degree: 8.0
888	PMAIP1	Degree: 8.0
889	FRK	Degree: 8.0
890	ZBTB20	Degree: 8.0
891	ASB4	Degree: 8.0
892	TCEAL2	Degree: 8.0
893	LSM1	Degree: 8.0
894	PIWIL4	Degree: 8.0
895	PTCH1	Degree: 8.0
896	FBXL15	Degree: 8.0
897	HIPK4	Degree: 8.0
898	RCBTB1	Degree: 8.0
899	TTC9C	Degree: 8.0
900	NLRP1	Degree: 8.0
901	HLA-DRA	Degree: 8.0
902	HLA-DRB5	Degree: 8.0
903	PTMS	Degree: 8.0
904	SPSB1	Degree: 8.0
905	TTI2	Degree: 8.0
906	CHD9	Degree: 7.0
907	HECTD3	Degree: 7.0
908	IRAK3	Degree: 7.0
909	PMF1	Degree: 7.0
910	TCF25	Degree: 7.0
911	REV1	Degree: 7.0
912	PDRG1	Degree: 7.0
913	NUAK2	Degree: 7.0
914	PIWIL1	Degree: 7.0
915	SLC12A3	Degree: 7.0
916	VPS18	Degree: 7.0

917	KLHL1	Degree: 7.0
918	[illegible]HOSPHO2-KLHL[illegible]	Degree: 7.0
919	PMF1-BGLAP	Degree: 7.0
920	RNF14	Degree: 7.0
921	KIAA0408	Degree: 7.0
922	MINK1	Degree: 7.0
923	PPIL2	Degree: 7.0
924	MERTK	Degree: 7.0
925	FLT4	Degree: 7.0
926	FBXL3	Degree: 7.0
927	ZBTB9	Degree: 7.0
928	METTL22	Degree: 7.0
929	KLHL23	Degree: 7.0
930	GMEB1	Degree: 7.0
931	MKKS	Degree: 7.0
932	NHLRC1	Degree: 7.0
933	BRSK2	Degree: 6.0
934	CASP12	Degree: 6.0
935	MAP3K6	Degree: 6.0
936	RARRES3	Degree: 6.0
937	CCDC117	Degree: 6.0
938	KLHL36	Degree: 6.0
939	SH3RF2	Degree: 6.0
940	RGS7	Degree: 6.0
941	TUBAL3	Degree: 6.0
942	C8orf44-SGK3	Degree: 6.0
943	HR	Degree: 6.0
944	KLHL29	Degree: 6.0
945	DDR1	Degree: 6.0
946	ZNF423	Degree: 6.0
947	ZBTB49	Degree: 6.0
948	CD3D	Degree: 6.0
949	MECR	Degree: 6.0
950	MYLK4	Degree: 6.0
951	TRIM56	Degree: 6.0
952	BBX	Degree: 6.0
953	FBXO27	Degree: 6.0
954	TRIM49	Degree: 6.0
955	SGK3	Degree: 6.0
956	TMF1	Degree: 6.0
957	RHOBTB1	Degree: 6.0
958	MSX2	Degree: 6.0
959	NFIC	Degree: 6.0
960	PCGF3	Degree: 6.0
961	MS4A2	Degree: 6.0
962	FLG	Degree: 6.0
963	MAP3K12	Degree: 6.0
964	ALDH5A1	Degree: 6.0
965	STK33	Degree: 6.0
966	SGK2	Degree: 6.0
967	TRIM10	Degree: 6.0
968	POR	Degree: 6.0
969	FBXO9	Degree: 6.0
970	FBXO34	Degree: 6.0

971	PRKY	Degree: 6.0
972	ARID5A	Degree: 6.0
973	TSPYL2	Degree: 6.0
974	FBXL14	Degree: 6.0
975	PTGDS	Degree: 6.0
976	BIK	Degree: 6.0
977	PTGS1	Degree: 6.0
978	PNRC1	Degree: 6.0
979	C22orf29	Degree: 6.0
980	ACVRL1	Degree: 6.0
981	ADA	Degree: 6.0
982	SPSB3	Degree: 6.0
983	TRIM74	Degree: 6.0
984	GZMA	Degree: 6.0
985	STK32C	Degree: 6.0
986	ASGR1	Degree: 6.0
987	DYRK4	Degree: 6.0
988	HLA-DRB3	Degree: 6.0
989	HLA-DRB4	Degree: 6.0
990	MAP4K2	Degree: 5.0
991	STK36	Degree: 5.0
992	HOXB1	Degree: 5.0
993	CDKL4	Degree: 5.0
994	RCBTB2	Degree: 5.0
995	RAPSN	Degree: 5.0
996	IFIT1	Degree: 5.0
997	TEKT4	Degree: 5.0
998	KBTBD4	Degree: 5.0
999	NEK11	Degree: 5.0
1000	MBOAT1	Degree: 5.0
1001	PRAG1	Degree: 5.0
1002	GTF2IRD2B	Degree: 5.0
1003	TAOK3	Degree: 5.0
1004	KLHL32	Degree: 5.0
1005	IP6K2	Degree: 5.0
1006	FBXO38	Degree: 5.0
1007	MCF2	Degree: 5.0
1008	KCNQ4	Degree: 5.0
1009	CAV2	Degree: 5.0
1010	TRIM2	Degree: 5.0
1011	KRT85	Degree: 5.0
1012	MUSK	Degree: 5.0
1013	EPHB6	Degree: 5.0
1014	GTF2IRD2	Degree: 5.0
1015	ITGB1BP2	Degree: 5.0
1016	BRSK1	Degree: 5.0
1017	PRR14L	Degree: 5.0
1018	CERK	Degree: 5.0
1019	ROR2	Degree: 5.0
1020	PREB	Degree: 5.0
1021	GRK6	Degree: 5.0
1022	VEGFA	Degree: 5.0
1023	AMHR2	Degree: 5.0
1024	PSKH2	Degree: 5.0

1025	PTGDR	Degree: 5.0
1026	HIF3A	Degree: 5.0
1027	BMF	Degree: 5.0
1028	UNC45B	Degree: 5.0
1029	GUCY1B3	Degree: 5.0
1030	NLRP4	Degree: 5.0
1031	HERC6	Degree: 5.0
1032	MKX	Degree: 5.0
1033	SLFN11	Degree: 5.0
1034	VPS41	Degree: 5.0
1035	EIF2AK1	Degree: 5.0
1036	PI4K2A	Degree: 5.0
1037	BIRC8	Degree: 4.0
1038	IFNGR2	Degree: 4.0
1039	ARMC5	Degree: 4.0
1040	ZMYM1	Degree: 4.0
1041	DTX4	Degree: 4.0
1042	MAP3K15	Degree: 4.0
1043	CYB5A	Degree: 4.0
1044	TRIM11	Degree: 4.0
1045	ZNF483	Degree: 4.0
1046	TSSK3	Degree: 4.0
1047	HRK	Degree: 4.0
1048	TYMP	Degree: 4.0
1049	KLHL14	Degree: 4.0
1050	THAP4	Degree: 4.0
1051	PHF3	Degree: 4.0
1052	NAIP	Degree: 4.0
1053	RGS6	Degree: 4.0
1054	DMPK	Degree: 4.0
1055	KLHL34	Degree: 4.0
1056	DOCK2	Degree: 4.0
1057	TESK1	Degree: 4.0
1058	HOPX	Degree: 4.0
1059	AREL1	Degree: 4.0
1060	EPHA1	Degree: 4.0
1061	TBX22	Degree: 4.0
1062	FLT3	Degree: 4.0
1063	RAB40A	Degree: 4.0
1064	CAMKV	Degree: 4.0
1065	FBXO10	Degree: 4.0
1066	MTRNR2L1	Degree: 4.0
1067	ALG2	Degree: 4.0
1068	PRKX	Degree: 4.0
1069	PIWIL2	Degree: 4.0
1070	MSTO1	Degree: 4.0
1071	PTGIS	Degree: 4.0
1072	KLHL25	Degree: 4.0
1073	KLHL6	Degree: 4.0
1074	ASPRV1	Degree: 4.0
1075	ACVR2B	Degree: 4.0
1076	STARD13	Degree: 4.0
1077	FAM162A	Degree: 4.0
1078	NOX5	Degree: 4.0

1079	GUCY1A2	Degree: 4.0
1080	PIM3	Degree: 4.0
1081	FBXL8	Degree: 4.0
1082	BBC3	Degree: 4.0
1083	FOXP2	Degree: 4.0
1084	ONECUT1	Degree: 4.0
1085	CDKL2	Degree: 4.0
1086	ALPK1	Degree: 3.0
1087	TRIM36	Degree: 3.0
1088	G2E3	Degree: 3.0
1089	CDC14A	Degree: 3.0
1090	CPT2	Degree: 3.0
1091	KLHL10	Degree: 3.0
1092	TRIM7	Degree: 3.0
1093	CD3G	Degree: 3.0
1094	CNOT6	Degree: 3.0
1095	SLC34A1	Degree: 3.0
1096	ECM1	Degree: 3.0
1097	KRT35	Degree: 3.0
1098	POGK	Degree: 3.0
1099	DAD1	Degree: 3.0
1100	PLCE1	Degree: 3.0
1101	WTIP	Degree: 3.0
1102	CERS2	Degree: 3.0
1103	RNF19B	Degree: 3.0
1104	MMP2	Degree: 3.0
1105	TSSK2	Degree: 3.0
1106	TIE1	Degree: 3.0
1107	ZBED4	Degree: 3.0
1108	AIPL1	Degree: 3.0
1109	AHSA2	Degree: 3.0
1110	ZNF74	Degree: 3.0
1111	RPS6KC1	Degree: 3.0
1112	TESK2	Degree: 3.0
1113	GRK4	Degree: 3.0
1114	MAPK4	Degree: 3.0
1115	PRKG2	Degree: 3.0
1116	DMRTA1	Degree: 3.0
1117	MYLK3	Degree: 3.0
1118	FASTK	Degree: 3.0
1119	STRADA	Degree: 3.0
1120	RGS11	Degree: 3.0
1121	TMEM54	Degree: 3.0
1122	PDIK1L	Degree: 3.0
1123	PI4K2B	Degree: 3.0
1124	STYK1	Degree: 3.0
1125	INSRR	Degree: 2.0
1126	KCNA6	Degree: 2.0
1127	KCNG1	Degree: 2.0
1128	DCLK2	Degree: 2.0
1129	KCNS3	Degree: 2.0
1130	ZC3H7B	Degree: 2.0
1131	STARD9	Degree: 2.0
1132	HACE1	Degree: 2.0

1133	CYP1A2	Degree: 2.0
1134	TNNI3K	Degree: 2.0
1135	FPGT-TNNI3K	Degree: 2.0
1136	MZB1	Degree: 2.0
1137	C20orf194	Degree: 2.0
1138	DLX6	Degree: 2.0
1139	RPS6KL1	Degree: 2.0
1140	CAMK1G	Degree: 2.0
1141	MAP3K9	Degree: 2.0
1142	MELK	Degree: 2.0
1143	ACVR1C	Degree: 2.0
1144	GRK7	Degree: 2.0
1145	ZNF215	Degree: 2.0
1146	FBXO40	Degree: 2.0
1147	MYO3B	Degree: 2.0
1148	FBXL13	Degree: 2.0
1149	PSKH1	Degree: 2.0
1150	NPRL2	Degree: 2.0
1151	TRIM73	Degree: 2.0
1152	ISX	Degree: 2.0
1153	KCTD8	Degree: 2.0
1154	9-Mar	Degree: 2.0
1155	COBLL1	Degree: 2.0
1156	PTPRN2	Degree: 2.0
1157	STK32B	Degree: 2.0
1158	SERPINB7	Degree: 1.0
1159	SIM1	Degree: 1.0
1160	NRIP2	Degree: 1.0
1161	IRX4	Degree: 1.0
1162	BOK	Degree: 1.0
