# Supplementary material for: Pharmacological Mechanisms Underlying the Therapeutic Effects of Danhong Injection on Cerebral Ischemia
Source: Evid Based Complement Alternat Med. 2021 May 21;2021:5584809. doi: 10.1155/2021/5584809 (PMC8163534; doi:10.1155/2021/5584809)
Supplement: Supplementary Materials — Table S1: the 37 candidate compounds of Danhong injection. Table S2: the 371 putative target proteins for the compounds. Table S3: the 413 IS-associated Homo sapiens target proteins from CTD with an inference score of ≥50. Table S4: the 61 IS-associated target proteins of Homo sapiens from Genecards with an inference score of ≥30. Table S5: degree centrality of nodes in PPI network. Table S6: betweenness centrality of nodes in the PPI network. Table S7: the GO functional enrichment analysis of diterpenoid quinones. Table S8: the KEGG pathway enrichment of diterpenoid quinones. Table S9: the KEGG pathway enrichment of DHI compounds. [file 5584809.f1.zip › 5584809.f1/S6 (2).pdf]

Table S6. Betweenness centrality of nodes in PPI network

1	HSP90AA1	Betweenness: 440637.1
2	HSP90AB1	Betweenness: 160691.73
3	NR3C1	Betweenness: 42507.926
4	NTRK1	Betweenness: 37304.836
5	APP	Betweenness: 31780.734
6	TP53	Betweenness: 28671.47
7	ESR1	Betweenness: 25899.0
8	XPO1	Betweenness: 19824.178
9	RARA	Betweenness: 17430.82
10	EGFR	Betweenness: 16055.506
11	AR	Betweenness: 14819.815
12	CUL3	Betweenness: 14575.848
13	COPS5	Betweenness: 13171.855
14	GRB2	Betweenness: 12238.085
15	HSPA4	Betweenness: 11545.129
16	HSPA8	Betweenness: 11533.224
17	MCL1	Betweenness: 11030.564
18	CDC37	Betweenness: 10898.5205
19	CDK2	Betweenness: 10280.154
20	BRCA1	Betweenness: 9636.571
21	TRAF6	Betweenness: 9180.394
22	EP300	Betweenness: 9079.927
23	YWHAZ	Betweenness: 8636.88
24	UBE2I	Betweenness: 8109.9062
25	VCP	Betweenness: 7977.6533
26	MCM2	Betweenness: 7928.4497
27	CUL1	Betweenness: 7828.135
28	STUB1	Betweenness: 7452.2935
29	HDAC1	Betweenness: 7271.084
30	AKT1	Betweenness: 7261.8696
31	IKBKG	Betweenness: 7128.492
32	SRC	Betweenness: 7051.1753
33	MDM2	Betweenness: 6653.796
34	HSPA5	Betweenness: 6497.3535
35	CREBBP	Betweenness: 6405.8203
36	MYC	Betweenness: 5954.024
37	BCL2	Betweenness: 5810.999
38	MAPK1	Betweenness: 5714.789
39	NPM1	Betweenness: 5497.8364
40	HSPB1	Betweenness: 5413.9956
41	HDAC5	Betweenness: 5371.241
42	FN1	Betweenness: 5186.6704
43	SNW1	Betweenness: 5114.7563
44	VHL	Betweenness: 5086.989
45	CDC5L	Betweenness: 5084.775
46	RNF2	Betweenness: 5020.2544
47	HUWE1	Betweenness: 4990.0845
48	PPARG	Betweenness: 4975.3257
49	YWHAG	Betweenness: 4962.7554
50	SMAD3	Betweenness: 4766.0737
51	JUN	Betweenness: 4457.718
52	GSK3B	Betweenness: 4219.4995

53	HNRNPA1	Betweenness: 4184.6855
54	YWHAQ	Betweenness: 4139.6704
55	YWHAB	Betweenness: 4023.5115
56	PARK2	Betweenness: 3822.7256
57	RPS27A	Betweenness: 3774.8008
58	TRAF2	Betweenness: 3751.8994
59	YWHAE	Betweenness: 3709.036
60	CAV1	Betweenness: 3689.2769
61	RELA	Betweenness: 3668.1248
62	PRKDC	Betweenness: 3656.4146
63	AURKA	Betweenness: 3640.7385
64	CASP9	Betweenness: 3560.4114
65	CUL2	Betweenness: 3559.2554
66	NFKB1	Betweenness: 3503.0662
67	CDKN1A	Betweenness: 3502.7957
68	BAG3	Betweenness: 3445.957
69	EEF1A1	Betweenness: 3362.1096
70	FKBP5	Betweenness: 3337.2168
71	RB1	Betweenness: 3325.3308
72	CDK1	Betweenness: 3303.1738
73	STAT3	Betweenness: 3282.1816
74	PARP1	Betweenness: 3060.3062
75	TUBB	Betweenness: 3052.0918
76	CAND1	Betweenness: 2988.0044
77	RXRA	Betweenness: 2962.9487
78	ABL1	Betweenness: 2905.3093
79	GAPDH	Betweenness: 2895.5125
80	FYN	Betweenness: 2865.4822
81	IKBKE	Betweenness: 2816.8237
82	RAF1	Betweenness: 2810.151
83	HSPA1A	Betweenness: 2797.278
84	HSPA1B	Betweenness: 2797.278
85	FBXO6	Betweenness: 2788.5178
86	PML	Betweenness: 2783.2527
87	SKP1	Betweenness: 2762.5247
88	HSPD1	Betweenness: 2726.637
89	CASP3	Betweenness: 2708.9983
90	UBE2N	Betweenness: 2699.071
91	MAPK3	Betweenness: 2681.9844
92	SHC1	Betweenness: 2659.5547
93	FUS	Betweenness: 2641.8572
94	CSNK2B	Betweenness: 2557.5906
95	TERF1	Betweenness: 2539.6057
96	HDAC2	Betweenness: 2507.7173
97	CUL4B	Betweenness: 2473.9036
98	HNRNPU	Betweenness: 2465.3564
99	VIM	Betweenness: 2459.267
100	MAPK8	Betweenness: 2424.015
101	CASP8	Betweenness: 2397.0825
102	CLTC	Betweenness: 2383.9426
103	TRIM28	Betweenness: 2380.6387
104	HIST3H3	Betweenness: 2321.1543
105	HDAC6	Betweenness: 2300.3103
106	USP7	Betweenness: 2292.51

107	ARRB1	Betweenness: 2273.8416
108	PTEN	Betweenness: 2201.1333
109	LYN	Betweenness: 2193.225
110	RACK1	Betweenness: 2189.9116
111	TUBA1A	Betweenness: 2177.7898
112	CUL5	Betweenness: 2115.3457
113	COPS6	Betweenness: 2110.0908
114	BTRC	Betweenness: 2105.6304
115	SP1	Betweenness: 2065.783
116	PKM	Betweenness: 2059.052
117	SQSTM1	Betweenness: 2048.5037
118	XIAP	Betweenness: 2027.78
119	CDC25B	Betweenness: 2026.1846
120	MYH9	Betweenness: 2024.2211
121	PIN1	Betweenness: 2022.2913
122	MAP3K1	Betweenness: 2000.3467
123	POR	Betweenness: 1995.4742
124	HDAC3	Betweenness: 1974.1768
125	MAPK6	Betweenness: 1973.9464
126	SRPK1	Betweenness: 1942.6907
127	UBL4A	Betweenness: 1927.7941
128	MAPK14	Betweenness: 1895.8522
129	MAP3K7	Betweenness: 1872.5654
130	STIP1	Betweenness: 1849.3892
131	NCL	Betweenness: 1848.4462
132	ESR2	Betweenness: 1838.0186
133	PCNA	Betweenness: 1816.3177
134	SVIL	Betweenness: 1794.6249
135	CDK9	Betweenness: 1767.7156
136	SIRT1	Betweenness: 1763.3158
137	LRRK2	Betweenness: 1757.7289
138	SMARCA4	Betweenness: 1748.5256
139	FBXW11	Betweenness: 1733.1782
140	EZH2	Betweenness: 1704.1758
141	CRY2	Betweenness: 1690.3455
142	SNCA	Betweenness: 1687.881
143	TAB1	Betweenness: 1678.5216
144	PTGES3	Betweenness: 1643.685
145	UBE2L3	Betweenness: 1633.388
146	MAP3K3	Betweenness: 1632.5736
147	ATF2	Betweenness: 1609.5701
148	DNAJA1	Betweenness: 1602.0637
149	NUDCD3	Betweenness: 1601.5487
150	SUMO2	Betweenness: 1596.3425
151	CCT2	Betweenness: 1588.092
152	CSNK2A2	Betweenness: 1573.3339
153	NEDD4L	Betweenness: 1571.8218
154	VCAM1	Betweenness: 1560.8303
155	U2AF2	Betweenness: 1560.7032
156	HSPA9	Betweenness: 1560.3303
157	RUVBL1	Betweenness: 1559.5269
158	HIF1A	Betweenness: 1548.1396
159	TOMM40	Betweenness: 1542.346
160	PRKCA	Betweenness: 1513.996

161	PRKAA1	Betweenness: 1502.625
162	TADA2A	Betweenness: 1502.3859
163	SIN3A	Betweenness: 1484.3031
164	BAG2	Betweenness: 1463.5831
165	FLNA	Betweenness: 1426.2816
166	PRKACA	Betweenness: 1425.8381
167	CHUK	Betweenness: 1422.042
168	BAX	Betweenness: 1417.0912
169	TRAF1	Betweenness: 1409.0833
170	CUL4A	Betweenness: 1406.673
171	EEF2	Betweenness: 1406.0237
172	PRKCD	Betweenness: 1394.9878
173	PPP2R1A	Betweenness: 1388.3865
174	CDK4	Betweenness: 1369.3243
175	PSMA3	Betweenness: 1361.8646
176	IKBKB	Betweenness: 1350.05
177	SUZ12	Betweenness: 1343.1786
178	HSPA1L	Betweenness: 1338.0098
179	FBXO25	Betweenness: 1324.5892
180	KAT5	Betweenness: 1323.8479
181	SFN	Betweenness: 1309.1359
182	NCOR1	Betweenness: 1293.6074
183	FBXW7	Betweenness: 1284.5118
184	BCL2L1	Betweenness: 1284.4758
185	AURKB	Betweenness: 1278.1497
186	VDAC1	Betweenness: 1271.4757
187	NR4A1	Betweenness: 1270.2726
188	CDKN2A	Betweenness: 1259.4236
189	SUMO1	Betweenness: 1240.5234
190	TNFRSF1A	Betweenness: 1235.692
191	SFPQ	Betweenness: 1215.4983
192	YWHAH	Betweenness: 1210.8148
193	PPARA	Betweenness: 1205.3073
194	UBE3A	Betweenness: 1192.9796
195	TARDBP	Betweenness: 1170.7473
196	TGFBR1	Betweenness: 1156.3062
197	TCP1	Betweenness: 1129.4542
198	MAP3K5	Betweenness: 1109.9053
199	PGR	Betweenness: 1067.4154
200	KAT2B	Betweenness: 1062.9583
201	PSMC5	Betweenness: 1062.2468
202	UBB	Betweenness: 1046.6187
203	NR1H3	Betweenness: 1017.99854
204	ETS1	Betweenness: 1007.9921
205	MCM5	Betweenness: 999.8001
206	NFE2L2	Betweenness: 998.63586
207	PRMT5	Betweenness: 992.3942
208	TUBG1	Betweenness: 971.08124
209	NCOA3	Betweenness: 970.74805
210	CDH1	Betweenness: 967.7587
211	ERBB2	Betweenness: 946.6401
212	PTK2	Betweenness: 937.74194
213	H2AFX	Betweenness: 935.0362
214	NCOA1	Betweenness: 930.0294

215	BAD	Betweenness: 927.03894
216	NCOR2	Betweenness: 922.04535
217	SUPT5H	Betweenness: 922.0207
218	ATP5B	Betweenness: 915.1482
219	PRKCZ	Betweenness: 907.6159
220	ATP5A1	Betweenness: 906.9447
221	PAN2	Betweenness: 901.2258
222	SPTAN1	Betweenness: 891.7318
223	CACYBP	Betweenness: 890.93317
224	NFKBIB	Betweenness: 885.1327
225	RPL5	Betweenness: 881.4325
226	IRAK1	Betweenness: 854.0701
227	TAB2	Betweenness: 851.1671
228	HCFC1	Betweenness: 835.72546
229	THRB	Betweenness: 832.4237
230	NUMA1	Betweenness: 826.14056
231	JAK2	Betweenness: 824.742
232	HMGB1	Betweenness: 821.5736
233	ACTG1	Betweenness: 820.4995
234	SKI	Betweenness: 816.36456
235	PAK1	Betweenness: 814.3623
236	TUBB4B	Betweenness: 808.4658
237	HDAC4	Betweenness: 808.1227
238	PCBP1	Betweenness: 808.0908
239	RPS16	Betweenness: 806.4039
240	NR1I2	Betweenness: 806.2514
241	CANX	Betweenness: 805.18475
242	KPNA2	Betweenness: 805.0691
243	PPARD	Betweenness: 804.4205
244	RPS3A	Betweenness: 797.5739
245	DNAJA3	Betweenness: 789.2242
246	DCUN1D1	Betweenness: 785.03503
247	HSF1	Betweenness: 784.6556
248	NR1I3	Betweenness: 782.3391
249	DYNC1H1	Betweenness: 775.94586
250	HNF4A	Betweenness: 775.5786
251	SF3B3	Betweenness: 775.3069
252	BCL6	Betweenness: 770.3575
253	NUDC	Betweenness: 769.75714
254	LCK	Betweenness: 756.3032
255	ZBTB16	Betweenness: 755.43604
256	CALR	Betweenness: 750.13837
257	SKP2	Betweenness: 746.2545
258	CREB1	Betweenness: 745.1523
259	NEDD8	Betweenness: 743.94995
260	TBK1	Betweenness: 742.1855
261	MYOD1	Betweenness: 741.7917
262	HMGA1	Betweenness: 741.5134
263	HNRNPR	Betweenness: 739.17303
264	BTK	Betweenness: 730.5949
265	RPS6	Betweenness: 729.7297
266	CEBPA	Betweenness: 715.13763
267	ATF3	Betweenness: 713.58093
268	ATP1B1	Betweenness: 709.996

269	SETDB1	Betweenness: 704.1297
270	RPS4X	Betweenness: 693.8852
271	PRKAB1	Betweenness: 686.70447
272	RPS3	Betweenness: 682.16046
273	ACTN1	Betweenness: 680.5178
274	PHB2	Betweenness: 679.92395
275	TERF2	Betweenness: 672.2674
276	RPSA	Betweenness: 670.51483
277	SET	Betweenness: 670.021
278	CDC25C	Betweenness: 662.4289
279	GRK2	Betweenness: 661.91583
280	USP9X	Betweenness: 654.4199
281	TNFAIP3	Betweenness: 648.3428
282	MAP2K1	Betweenness: 646.9565
283	SNRNP200	Betweenness: 636.6966
284	RPS6KB2	Betweenness: 634.09534
285	E2F1	Betweenness: 629.96063
286	TSG101	Betweenness: 622.9818
287	TBP	Betweenness: 621.10626
288	FAS	Betweenness: 618.58685
289	MAP3K14	Betweenness: 618.38354
290	DAXX	Betweenness: 616.6338
291	GNB2	Betweenness: 615.2377
292	SREBF1	Betweenness: 612.6572
293	RAD21	Betweenness: 611.76074
294	NCOA2	Betweenness: 606.2399
295	CBX5	Betweenness: 605.06415
296	IRS4	Betweenness: 603.8319
297	TUFM	Betweenness: 600.90906
298	ILK	Betweenness: 600.2319
299	SIAH1	Betweenness: 589.8639
300	RAN	Betweenness: 583.37634
301	CAMK2A	Betweenness: 575.84717
302	TPM3	Betweenness: 573.2901
303	COPS2	Betweenness: 569.7982
304	PGK1	Betweenness: 569.0419
305	CDK5	Betweenness: 563.7585
306	BECN1	Betweenness: 559.741
307	FKBP4	Betweenness: 557.08124
308	EPAS1	Betweenness: 554.52423
309	RIPK2	Betweenness: 554.1743
310	NFKB2	Betweenness: 553.68695
311	MAX	Betweenness: 543.6215
312	IRF3	Betweenness: 542.8599
313	ZBTB17	Betweenness: 540.2583
314	SSBP1	Betweenness: 537.6856
315	STK11	Betweenness: 533.6479
316	AHSA1	Betweenness: 531.5825
317	TRIM10	Betweenness: 528.83673
318	CDK7	Betweenness: 524.309
319	CASP7	Betweenness: 518.66473
320	SUGT1	Betweenness: 518.5918
321	RIPK1	Betweenness: 517.3433
322	SF3A1	Betweenness: 513.56714

323	ACTN4	Betweenness: 507.67526
324	MED1	Betweenness: 507.47766
325	ARAF	Betweenness: 505.87582
326	RANBP9	Betweenness: 504.72256
327	EIF2AK2	Betweenness: 492.4262
328	LNX1	Betweenness: 492.29373
329	BAG1	Betweenness: 484.62238
330	CDK6	Betweenness: 484.58017
331	THRAP3	Betweenness: 483.59735
332	CEP250	Betweenness: 483.23456
333	IRAK3	Betweenness: 481.3448
334	DNAJC7	Betweenness: 477.04697
335	CYB5A	Betweenness: 476.57135
336	HSPE1	Betweenness: 468.3187
337	SLC25A5	Betweenness: 467.9846
338	USP19	Betweenness: 467.71176
339	NR0B2	Betweenness: 465.76254
340	GNAI2	Betweenness: 464.9405
341	WWOX	Betweenness: 463.19058
342	PSMD1	Betweenness: 462.42963
343	LRIF1	Betweenness: 461.32855
344	SMARCB1	Betweenness: 459.6281
345	GNB1	Betweenness: 458.89273
346	HSPA2	Betweenness: 457.6507
347	ISG15	Betweenness: 454.46085
348	BCR	Betweenness: 454.35233
349	KMT2A	Betweenness: 451.36063
350	CAMK2D	Betweenness: 451.25385
351	NR2C2	Betweenness: 450.67307
352	RPL3	Betweenness: 450.1499
353	TGFBR2	Betweenness: 447.31683
354	CD2AP	Betweenness: 445.28918
355	TNK2	Betweenness: 441.9108
356	EFTUD2	Betweenness: 441.56717
357	AGO2	Betweenness: 441.3592
358	GRK5	Betweenness: 437.79034
359	BIRC2	Betweenness: 436.50256
360	ANAPC2	Betweenness: 433.57376
361	SRF	Betweenness: 432.71985
362	MMS19	Betweenness: 432.038
363	BIRC5	Betweenness: 431.62592
364	SMARCC1	Betweenness: 431.62433
365	FASN	Betweenness: 431.3166
366	CEP76	Betweenness: 430.42773
367	PRKAA2	Betweenness: 430.2889
368	PPARGC1A	Betweenness: 426.59164
369	BCL2L11	Betweenness: 426.07187
370	CYCS	Betweenness: 423.96362
371	PRPF19	Betweenness: 423.93103
372	EPRS	Betweenness: 416.72363
373	FANCC	Betweenness: 411.13568
374	CFLAR	Betweenness: 409.9203
375	PRKCE	Betweenness: 407.92896
376	MAGEA11	Betweenness: 405.22528

377	PTGS2	Betweenness: 404.50833
378	FOXD4L6	Betweenness: 401.78275
379	MET	Betweenness: 400.5226
380	PIAS2	Betweenness: 390.12152
381	FOXM1	Betweenness: 390.11914
382	RPLP0	Betweenness: 389.65872
383	SMARCE1	Betweenness: 389.41763
384	TUBB2B	Betweenness: 388.88306
385	AIP	Betweenness: 388.5059
386	TXN	Betweenness: 385.95087
387	KIAA1549	Betweenness: 378.28958
388	RPL23A	Betweenness: 378.00574
389	BID	Betweenness: 375.6477
390	YES1	Betweenness: 367.87848
391	NCOA6	Betweenness: 366.37784
392	CSNK1A1	Betweenness: 365.8371
393	CHEK1	Betweenness: 359.86142
394	RIPK3	Betweenness: 357.58588
395	RPL7	Betweenness: 357.53058
396	LDHA	Betweenness: 357.22092
397	UCHL1	Betweenness: 356.6549
398	RPS6KA1	Betweenness: 355.15445
399	MTA2	Betweenness: 353.38116
400	MAPK7	Betweenness: 349.98044
401	BIRC3	Betweenness: 345.6505
402	KDR	Betweenness: 344.39722
403	SSB	Betweenness: 343.2186
404	RBCK1	Betweenness: 341.1103
405	SGTA	Betweenness: 337.61282
406	KEAP1	Betweenness: 336.7505
407	MCM3	Betweenness: 336.60736
408	CLU	Betweenness: 336.46384
409	UBQLN1	Betweenness: 335.2715
410	RBM14-RBM4	Betweenness: 334.71445
411	RBM14	Betweenness: 334.71445
412	NUDCD2	Betweenness: 333.21286
413	TEAD2	Betweenness: 332.42407
414	PRPF8	Betweenness: 329.32263
415	NOD1	Betweenness: 329.27695
416	PRDX6	Betweenness: 329.19952
417	RUNX1	Betweenness: 327.11752
418	WWP1	Betweenness: 326.543
419	CEBPB	Betweenness: 325.76602
420	DIABLO	Betweenness: 322.7569
421	NRIP1	Betweenness: 319.83997
422	SENP3	Betweenness: 318.7627
423	SGK1	Betweenness: 317.69354
424	P4HB	Betweenness: 312.4189
425	APAF1	Betweenness: 310.74298
426	GSK3A	Betweenness: 310.3426
427	SUV39H1	Betweenness: 309.96918
428	SMARCA2	Betweenness: 307.19952
429	TP53BP1	Betweenness: 306.47275
430	PCGF1	Betweenness: 303.67474

431	JAK1	Betweenness: 301.80737
432	GAN	Betweenness: 301.65152
433	CFTR	Betweenness: 298.34442
434	EIF3H	Betweenness: 298.05917
435	BRAF	Betweenness: 297.679
436	DSP	Betweenness: 294.68506
437	CDK13	Betweenness: 291.96347
438	TRIM32	Betweenness: 291.18646
439	UBA1	Betweenness: 287.48035
440	DYRK2	Betweenness: 287.01672
441	DNAJA2	Betweenness: 286.4951
442	MTRNR2L1	Betweenness: 285.18927
443	AKT2	Betweenness: 280.40463
444	ATR	Betweenness: 278.87045
445	POLR2E	Betweenness: 273.77606
446	PPP5C	Betweenness: 272.18442
447	KBTBD7	Betweenness: 268.98276
448	MAPK9	Betweenness: 268.0797
449	FKBP8	Betweenness: 265.29684
450	QARS	Betweenness: 265.2232
451	CRYAB	Betweenness: 264.98633
452	POU2F1	Betweenness: 261.72803
453	STAT5B	Betweenness: 260.8781
454	TTC1	Betweenness: 259.25943
455	MAP2K7	Betweenness: 256.96573
456	NR3C2	Betweenness: 256.40445
457	BCL10	Betweenness: 256.24033
458	LGALS3BP	Betweenness: 256.13242
459	MDM4	Betweenness: 255.21666
460	DYNLT1	Betweenness: 255.17406
461	HNRNPL	Betweenness: 254.1146
462	KRT31	Betweenness: 253.74532
463	RPAP3	Betweenness: 253.288
464	UQCRC2	Betweenness: 253.12088
465	CNOT1	Betweenness: 253.10786
466	CAMK2B	Betweenness: 252.5428
467	EIF4B	Betweenness: 251.777
468	ESRRB	Betweenness: 248.58606
469	AMBRA1	Betweenness: 248.09247
470	MGMT	Betweenness: 247.22556
471	PIH1D1	Betweenness: 246.12872
472	FER	Betweenness: 244.8566
473	FANCA	Betweenness: 244.0019
474	RPS6KB1	Betweenness: 243.07472
475	CEP57	Betweenness: 241.29655
476	TYK2	Betweenness: 239.74876
477	HSPA6	Betweenness: 239.72227
478	RUNX1T1	Betweenness: 239.33258
479	ALDOA	Betweenness: 238.60077
480	NOS2	Betweenness: 237.9601
481	STAT5A	Betweenness: 237.67368
482	TRIM37	Betweenness: 235.92885
483	BLM	Betweenness: 232.32948
484	NR1H2	Betweenness: 231.17159

485	FGFR1OP	Betweenness: 230.95265
486	DET1	Betweenness: 228.46417
487	CAMK2G	Betweenness: 227.94336
488	RPS15A	Betweenness: 227.46184
489	FKBPL	Betweenness: 227.02122
490	PINK1	Betweenness: 226.22174
491	RAD51	Betweenness: 224.11685
492	PKN1	Betweenness: 221.72803
493	GATA2	Betweenness: 221.31787
494	PPID	Betweenness: 219.60074
495	SNRPE	Betweenness: 219.07643
496	EPB41L3	Betweenness: 218.62404
497	ARNTL	Betweenness: 215.1777
498	PRKCI	Betweenness: 215.00108
499	CKS1B	Betweenness: 214.77536
500	CCDC6	Betweenness: 213.42174
501	EGLN1	Betweenness: 212.48518
502	MOS	Betweenness: 211.42183
503	BRMS1	Betweenness: 210.09901
504	GADD45A	Betweenness: 209.7814
505	SMARCD3	Betweenness: 208.36693
506	PRKD1	Betweenness: 206.27075
507	TRADD	Betweenness: 206.10197
508	NEK9	Betweenness: 205.65672
509	DSN1	Betweenness: 204.0686
510	ERBB3	Betweenness: 203.48871
511	NDRG1	Betweenness: 202.56607
512	PRKCB	Betweenness: 202.12686
513	PDGFRB	Betweenness: 199.0777
514	CCND3	Betweenness: 198.78316
515	VARS	Betweenness: 197.7962
516	PKN2	Betweenness: 197.68103
517	RPS6KA5	Betweenness: 196.33647
518	IRF1	Betweenness: 193.37784
519	BAG4	Betweenness: 193.30827
520	PRPF6	Betweenness: 191.67847
521	PAFAH1B1	Betweenness: 188.58557
522	TP53RK	Betweenness: 188.135
523	RXRB	Betweenness: 187.84213
524	DDOST	Betweenness: 186.8436
525	CDK18	Betweenness: 186.81306
526	PSMB5	Betweenness: 184.58435
527	CEBPE	Betweenness: 183.89438
528	CAPN2	Betweenness: 183.4022
529	CRNKL1	Betweenness: 183.03148
530	LIMK1	Betweenness: 182.86362
531	HMGB2	Betweenness: 182.511
532	CDK11B	Betweenness: 182.20395
533	PIK3C3	Betweenness: 181.90302
534	COPS3	Betweenness: 181.19179
535	CDK3	Betweenness: 180.4588
536	UNC45A	Betweenness: 179.31789
537	BRCC3	Betweenness: 179.27051
538	RFC4	Betweenness: 179.02617

539	AHR	Betweenness: 178.8817
540	MAPKAPK2	Betweenness: 178.71683
541	BBX	Betweenness: 177.15492
542	NPAS2	Betweenness: 176.93529
543	CENPB	Betweenness: 176.85394
544	FBXL12	Betweenness: 176.80711
545	STAT2	Betweenness: 172.91876
546	RPS6KA3	Betweenness: 172.76744
547	TERT	Betweenness: 172.68881
548	SCRIB	Betweenness: 171.88606
549	FLII	Betweenness: 171.8539
550	LRSAM1	Betweenness: 171.04097
551	PRKAB2	Betweenness: 170.73436
552	SRSF5	Betweenness: 167.50316
553	HRAS	Betweenness: 166.32806
554	RPS20	Betweenness: 165.78697
555	CD4	Betweenness: 164.12654
556	AGO3	Betweenness: 163.78156
557	NFRKB	Betweenness: 163.33731
558	MED14	Betweenness: 161.22726
559	MAFG	Betweenness: 159.50392
560	PTK2B	Betweenness: 159.4451
561	HK2	Betweenness: 158.90166
562	NR2F2	Betweenness: 157.1144
563	ALK	Betweenness: 155.84714
564	CHORDC1	Betweenness: 153.65773
565	TBL1XR1	Betweenness: 152.80223
566	MARS	Betweenness: 152.54482
567	TNIP1	Betweenness: 150.68764
568	HSP90AA5P	Betweenness: 150.25674
569	UHRF2	Betweenness: 149.74759
570	HSPH1	Betweenness: 148.10548
571	BAK1	Betweenness: 147.95813
572	SNRNP40	Betweenness: 147.75961
573	CLK3	Betweenness: 146.91534
574	NOS3	Betweenness: 146.38219
575	MIS12	Betweenness: 146.36826
576	CDK11A	Betweenness: 145.54187
577	CDK14	Betweenness: 144.70567
578	MAP3K2	Betweenness: 142.0642
579	FBXW2	Betweenness: 142.05927
580	PPP3CA	Betweenness: 141.96233
581	RAG1	Betweenness: 141.90894
582	ERN1	Betweenness: 140.71176
583	CDK5R1	Betweenness: 139.68881
584	KLF5	Betweenness: 139.31723
585	SMARCD1	Betweenness: 139.20868
586	TRIM24	Betweenness: 137.03064
587	RGS7	Betweenness: 136.62277
588	DNMT3L	Betweenness: 136.60907
589	SSR4	Betweenness: 136.46031
590	ACP1	Betweenness: 135.17188
591	ZAP70	Betweenness: 132.98027
592	RPL32	Betweenness: 132.38622

593	GRK6	Betweenness: 132.24406
594	ERBB4	Betweenness: 131.98325
595	TOP2B	Betweenness: 130.79782
596	FHL3	Betweenness: 130.44171
597	IQCB1	Betweenness: 130.27275
598	MAGED2	Betweenness: 129.43738
599	NEK8	Betweenness: 127.686935
600	RXRG	Betweenness: 127.08785
601	FOXO1	Betweenness: 125.996414
602	ANLN	Betweenness: 125.982605
603	CDC73	Betweenness: 124.796104
604	EIF4A2	Betweenness: 124.428665
605	LARP4B	Betweenness: 122.42365
606	PRKACB	Betweenness: 122.07839
607	PRKAG1	Betweenness: 119.773544
608	DYRK1B	Betweenness: 119.25845
609	NFKBIE	Betweenness: 118.263565
610	NLRP3	Betweenness: 117.57421
611	ASB2	Betweenness: 117.23584
612	SPEN	Betweenness: 115.73975
613	EIF5B	Betweenness: 115.2486
614	HCK	Betweenness: 114.66905
615	IRF2	Betweenness: 114.50486
616	PRKD2	Betweenness: 113.401276
617	FKBP6	Betweenness: 113.31137
618	MAP3K11	Betweenness: 111.855865
619	MAP3K8	Betweenness: 111.830986
620	NUCB1	Betweenness: 111.60313
621	RPS6KA6	Betweenness: 109.78623
622	AREL1	Betweenness: 109.26784
623	RPL29	Betweenness: 108.620346
624	FOXL1	Betweenness: 108.528854
625	TOMM70	Betweenness: 108.17293
626	FGFR1	Betweenness: 107.214035
627	PRAME	Betweenness: 106.557465
628	TRIP4	Betweenness: 106.12832
629	PRKCQ	Betweenness: 105.90261
630	IGF1R	Betweenness: 105.064
631	GAPVD1	Betweenness: 103.65023
632	TJP1	Betweenness: 103.57295
633	RNF111	Betweenness: 103.27266
634	PI4K2A	Betweenness: 103.20062
635	MAPK15	Betweenness: 102.99798
636	MTHFD1	Betweenness: 101.585266
637	ACVR1B	Betweenness: 100.64717
638	FASTK	Betweenness: 100.41409
639	MAST2	Betweenness: 100.32187
640	GIGYF2	Betweenness: 99.87466
641	DARS	Betweenness: 99.830765
642	RPL17	Betweenness: 99.72543
643	PRPF4B	Betweenness: 99.56024
644	ETS2	Betweenness: 98.917816
645	FBXL18	Betweenness: 98.88732
646	NPHP4	Betweenness: 98.842064

647	MBD3	Betweenness: 98.775635
648	CAMKK2	Betweenness: 98.74887
649	DDX54	Betweenness: 98.49888
650	MAPK10	Betweenness: 98.00617
651	UBASH3B	Betweenness: 97.60006
652	DAP3	Betweenness: 97.59974
653	TJP2	Betweenness: 97.032166
654	BLK	Betweenness: 96.919525
655	RPS6KA2	Betweenness: 95.93802
656	PASK	Betweenness: 95.90647
657	RHOBTB3	Betweenness: 93.41403
658	WDR6	Betweenness: 93.34732
659	GADD45G	Betweenness: 92.8679
660	LARP7	Betweenness: 91.74114
661	AKT3	Betweenness: 91.314095
662	CARD8	Betweenness: 90.19892
663	SPI1	Betweenness: 88.26079
664	NLRP2	Betweenness: 87.17685
665	ASB3	Betweenness: 87.13984
666	GPR75-ASB3	Betweenness: 87.13984
667	HES1	Betweenness: 86.62942
668	FTH1	Betweenness: 86.4668
669	AP3D1	Betweenness: 86.01842
670	LMAN1	Betweenness: 85.86893
671	SLFN11	Betweenness: 85.73468
672	SKIV2L2	Betweenness: 85.43977
673	PELP1	Betweenness: 84.337204
674	EPHA2	Betweenness: 84.277374
675	GNAQ	Betweenness: 82.88974
676	KLHL38	Betweenness: 80.84623
677	SRPK3	Betweenness: 80.11578
678	DAPK1	Betweenness: 79.94014
679	HMOX2	Betweenness: 79.36574
680	ASB15	Betweenness: 78.52636
681	WASL	Betweenness: 78.52636
682	PPP6R3	Betweenness: 77.80467
683	DAPK3	Betweenness: 77.73541
684	ST13	Betweenness: 77.72932
685	MAP3K6	Betweenness: 76.99678
686	MAP3K15	Betweenness: 76.5128
687	TFDP3	Betweenness: 76.22939
688	BMX	Betweenness: 75.2264
689	SUMO4	Betweenness: 75.19687
690	URI1	Betweenness: 74.6959
691	ACVRL1	Betweenness: 74.365906
692	CALD1	Betweenness: 73.91324
693	HP1BP3	Betweenness: 73.631516
694	CASP10	Betweenness: 73.276344
695	ECD	Betweenness: 73.15077
696	ATG12	Betweenness: 72.51468
697	ARID1A	Betweenness: 72.33104
698	HSD17B4	Betweenness: 72.3137
699	FBXW5	Betweenness: 71.927826
700	TPT1	Betweenness: 71.74973

701	RALBP1	Betweenness: 71.57408
702	FBXL3	Betweenness: 71.14605
703	MED6	Betweenness: 71.088486
704	ZHX1	Betweenness: 71.01195
705	TRAF3IP2	Betweenness: 71.00963
706	AJUBA	Betweenness: 70.98419
707	STARD13	Betweenness: 70.13126
708	MID1	Betweenness: 70.092255
709	AARS	Betweenness: 69.96043
710	NSD1	Betweenness: 69.91382
711	PRKCG	Betweenness: 68.4625
712	TTC3	Betweenness: 67.3024
713	PBX1	Betweenness: 67.06462
714	TCF25	Betweenness: 66.95066
715	RNF10	Betweenness: 66.50653
716	PRAM1	Betweenness: 66.17613
717	KCNH2	Betweenness: 65.71874
718	TSSK1B	Betweenness: 65.32298
719	RCAN1	Betweenness: 65.24107
720	NES	Betweenness: 65.16763
721	HLA-DRA	Betweenness: 64.731766
722	MBD1	Betweenness: 64.56435
723	NCOA4	Betweenness: 64.56419
724	AGO1	Betweenness: 64.41321
725	KSR1	Betweenness: 64.06421
726	EDRF1	Betweenness: 63.87075
727	MAFF	Betweenness: 63.748257
728	TRIM74	Betweenness: 63.733192
729	SLC25A4	Betweenness: 63.58382
730	FOXJ2	Betweenness: 63.107456
731	RNF34	Betweenness: 62.802914
732	ANP32A	Betweenness: 62.13373
733	FAM83H	Betweenness: 61.706387
734	PHF8	Betweenness: 61.500374
735	TADA3	Betweenness: 61.224995
736	BIK	Betweenness: 60.43303
737	AURKC	Betweenness: 60.260803
738	TNKS2	Betweenness: 59.809566
739	CXXC1	Betweenness: 59.779167
740	ALG2	Betweenness: 59.50323
741	BMPR1A	Betweenness: 59.375614
742	PIM1	Betweenness: 59.082882
743	ZNF496	Betweenness: 58.980946
744	CDK15	Betweenness: 58.980293
745	BTG1	Betweenness: 58.67725
746	FES	Betweenness: 58.498104
747	HLA-DRB5	Betweenness: 58.496284
748	SOCS6	Betweenness: 58.49106
749	MED25	Betweenness: 58.471046
750	MAGEB2	Betweenness: 57.781593
751	TGFB1I1	Betweenness: 57.238754
752	ITGB1BP2	Betweenness: 57.128616
753	GRIP1	Betweenness: 56.9275
754	STK38L	Betweenness: 56.843628

755	SMYD2	Betweenness: 56.33934
756	MLF2	Betweenness: 56.3105
757	TTC5	Betweenness: 56.217995
758	HLA-DRB3	Betweenness: 55.853527
759	HLA-DRB4	Betweenness: 55.853527
760	KCNA5	Betweenness: 55.702637
761	TRIM41	Betweenness: 55.638798
762	WDR76	Betweenness: 55.181267
763	TYRO3	Betweenness: 54.8826
764	FNIP1	Betweenness: 54.78221
765	RNF40	Betweenness: 54.493797
766	PSMD10	Betweenness: 54.3852
767	APOB	Betweenness: 54.339138
768	OSGEP	Betweenness: 54.178253
769	MRTO4	Betweenness: 53.63041
770	NRBF2	Betweenness: 53.213905
771	CAMK4	Betweenness: 52.6849
772	EDNRA	Betweenness: 52.561924
773	NSL1	Betweenness: 52.552334
774	MYOCD	Betweenness: 52.404778
775	XRCC1	Betweenness: 51.738224
776	BIRC6	Betweenness: 51.7313
777	MAP2K2	Betweenness: 51.29566
778	AGO4	Betweenness: 51.2862
779	SSR3	Betweenness: 51.282066
780	CD3D	Betweenness: 51.23405
781	ZBTB20	Betweenness: 50.68071
782	TDG	Betweenness: 50.435516
783	SRA1	Betweenness: 50.177696
784	HSP90AB2P	Betweenness: 49.90335
785	HERC4	Betweenness: 49.62895
786	MAP4K4	Betweenness: 49.59275
787	PSAT1	Betweenness: 49.453915
788	LIMD1	Betweenness: 48.691322
789	TRAF3IP1	Betweenness: 48.532917
790	CLOCK	Betweenness: 48.250057
791	DCAF6	Betweenness: 47.924927
792	MAPK4	Betweenness: 47.47035
793	GZMB	Betweenness: 47.074448
794	PRDM1	Betweenness: 46.88497
795	ASXL1	Betweenness: 46.31928
796	FBXW8	Betweenness: 45.981346
797	SPSB1	Betweenness: 45.41552
798	PTMS	Betweenness: 45.404972
799	ICK	Betweenness: 45.305405
800	TNKS	Betweenness: 45.165207
801	TXNDC5	Betweenness: 44.296406
802	EPHB1	Betweenness: 44.05849
803	SAP130	Betweenness: 43.9619
804	SLC2A4	Betweenness: 43.897156
805	USP49	Betweenness: 43.154182
806	SMYD3	Betweenness: 42.60988
807	HSP90AB3P	Betweenness: 42.591072
808	CLK2	Betweenness: 42.573406

809	NTRK3	Betweenness: 41.662136
810	EEF1AKMT3	Betweenness: 41.09896
811	PCGF6	Betweenness: 40.97284
812	CEP97	Betweenness: 40.80083
813	CAV2	Betweenness: 40.25537
814	MAP4K1	Betweenness: 40.0736
815	TTC4	Betweenness: 39.668972
816	HSPA7	Betweenness: 39.47951
817	MIF	Betweenness: 39.234905
818	NANS	Betweenness: 39.110954
819	DDR1	Betweenness: 38.60366
820	SMG1	Betweenness: 38.546326
821	NFIC	Betweenness: 38.405266
822	RAD9A	Betweenness: 37.769863
823	GNA12	Betweenness: 37.32493
824	COBLL1	Betweenness: 36.528053
825	TRIM36	Betweenness: 35.969048
826	CHTF18	Betweenness: 35.921738
827	PTGIS	Betweenness: 35.83564
828	ASB17	Betweenness: 35.658722
829	KSR2	Betweenness: 35.630505
830	HOPX	Betweenness: 35.467785
831	PIM2	Betweenness: 35.42313
832	RNF114	Betweenness: 35.38855
833	HIPK4	Betweenness: 35.230343
834	ENC1	Betweenness: 34.940994
835	EIF2AK3	Betweenness: 34.079067
836	TRIM11	Betweenness: 33.981
837	SCO2	Betweenness: 33.903736
838	TRIM2	Betweenness: 33.79687
839	NTRK2	Betweenness: 33.311874
840	FAM103A1	Betweenness: 32.911995
841	MDH1	Betweenness: 32.84501
842	HIP1	Betweenness: 32.57912
843	PRKCH	Betweenness: 32.28084
844	AXL	Betweenness: 32.24202
845	NR2F6	Betweenness: 32.202637
846	FGFR3	Betweenness: 32.158035
847	ZBTB9	Betweenness: 32.13709
848	IDE	Betweenness: 31.906687
849	MST1R	Betweenness: 31.445854
850	PSMC3IP	Betweenness: 31.345078
851	PTK6	Betweenness: 31.300985
852	PPIL2	Betweenness: 30.870228
853	WSB2	Betweenness: 30.791906
854	RGS6	Betweenness: 30.689398
855	RET	Betweenness: 30.42435
856	MAP2K5	Betweenness: 30.389399
857	RABEP2	Betweenness: 30.30441
858	POU2F2	Betweenness: 29.853493
859	WNK4	Betweenness: 29.393091
860	C12orf10	Betweenness: 29.296328
861	VEGFA	Betweenness: 29.126385
862	ZNF483	Betweenness: 28.799692

863	G2E3	Betweenness: 28.645456
864	MGEA5	Betweenness: 28.547173
865	MKX	Betweenness: 28.441425
866	RNF14	Betweenness: 28.385523
867	FGR	Betweenness: 28.336731
868	NKX2-1	Betweenness: 28.24197
869	ITK	Betweenness: 28.203592
870	EPB41L2	Betweenness: 28.201775
871	FBXL15	Betweenness: 28.144089
872	CAMKK1	Betweenness: 28.055704
873	HDAC8	Betweenness: 27.931332
874	SIM2	Betweenness: 27.828154
875	ARPC5	Betweenness: 27.636488
876	PRRC2C	Betweenness: 27.600424
877	RAD52	Betweenness: 27.113928
878	MARCKS	Betweenness: 27.091385
879	WDR20	Betweenness: 27.018398
880	KLHL13	Betweenness: 26.758957
881	TNK1	Betweenness: 26.45798
882	TOMM34	Betweenness: 26.313997
883	CLIP1	Betweenness: 26.189018
884	ALDH5A1	Betweenness: 25.621725
885	TRIM7	Betweenness: 25.403645
886	DMRTA1	Betweenness: 25.340351
887	BIRC7	Betweenness: 25.05987
888	DCC	Betweenness: 24.682177
889	ARID5A	Betweenness: 24.581291
890	NLRP1	Betweenness: 24.461758
891	KIAA0408	Betweenness: 23.672878
892	PTCH1	Betweenness: 23.51619
893	NR0B1	Betweenness: 23.357159
894	GZMA	Betweenness: 23.346369
895	KATNA1	Betweenness: 22.970203
896	LRP1	Betweenness: 22.685026
897	CNOT6	Betweenness: 22.209736
898	STK38	Betweenness: 22.103088
899	PTGS1	Betweenness: 21.951624
900	ZBTB3	Betweenness: 21.859102
901	HERC6	Betweenness: 21.741856
902	KLHL26	Betweenness: 21.633331
903	POU1F1	Betweenness: 21.326323
904	EPHA4	Betweenness: 21.232794
905	RNF19B	Betweenness: 20.693571
906	TRIM17	Betweenness: 20.579348
907	TXN2	Betweenness: 20.527666
908	IRAK2	Betweenness: 20.299145
909	MSX2	Betweenness: 20.286762
910	PMF1	Betweenness: 20.160376
911	PMF1-BGLAP	Betweenness: 20.160376
912	TMF1	Betweenness: 20.114233
913	CDC37L1	Betweenness: 19.652487
914	CAP1	Betweenness: 19.575787
915	PAK6	Betweenness: 19.26245
916	BUB1B-PAK6	Betweenness: 19.26245

917	ASB6	Betweenness: 19.22559
918	CCNH	Betweenness: 18.850271
919	PIM3	Betweenness: 18.428791
920	FLT3	Betweenness: 18.324512
921	FBXO18	Betweenness: 18.316872
922	FBXL2	Betweenness: 18.248173
923	VPS18	Betweenness: 17.956415
924	SYT1	Betweenness: 17.83026
925	RAD54L2	Betweenness: 17.613365
926	PCGF3	Betweenness: 17.589882
927	MINK1	Betweenness: 17.544586
928	MERTK	Betweenness: 17.415634
929	LSM1	Betweenness: 17.406769
930	USP50	Betweenness: 17.320715
931	PACRG	Betweenness: 17.277332
932	POLH	Betweenness: 17.22916
933	PMAIP1	Betweenness: 16.997496
934	GMEB1	Betweenness: 16.823349
935	RPS6KC1	Betweenness: 16.708582
936	FOXP2	Betweenness: 16.680828
937	TCEAL2	Betweenness: 16.648563
938	FBXO17	Betweenness: 16.600582
939	KTN1	Betweenness: 16.566652
940	IARS2	Betweenness: 16.232485
941	C8orf44-SGK3	Betweenness: 16.128971
942	SGK3	Betweenness: 16.128971
943	ECM1	Betweenness: 15.898632
944	PTGDR	Betweenness: 15.443763
945	PLCE1	Betweenness: 15.33589
946	PREB	Betweenness: 15.029052
947	FBXO28	Betweenness: 14.991324
948	SGK2	Betweenness: 14.986885
949	FBXO3	Betweenness: 14.942539
950	RARRES3	Betweenness: 13.545614
951	PTGDS	Betweenness: 13.365463
952	FLG	Betweenness: 13.2534275
953	PNRC2	Betweenness: 13.233233
954	ASGR1	Betweenness: 12.976962
955	MCF2	Betweenness: 12.84928
956	REV1	Betweenness: 12.690011
957	IFNGR2	Betweenness: 12.6410475
958	NR2E3	Betweenness: 12.310203
959	TTC9C	Betweenness: 12.126687
960	MATK	Betweenness: 12.051113
961	BRSK2	Betweenness: 11.978362
962	CHD9	Betweenness: 11.898258
963	PRKX	Betweenness: 11.867471
964	RFWD3	Betweenness: 11.698492
965	PIWIL4	Betweenness: 11.076938
966	SPSB3	Betweenness: 11.076094
967	CPT2	Betweenness: 10.650807
968	HECTD3	Betweenness: 10.612655
969	NLRP12	Betweenness: 10.5740185
970	CWC15	Betweenness: 10.573329

971	PRAG1	Betweenness: 10.352616
972	MAP3K12	Betweenness: 10.276871
973	FBXO24	Betweenness: 9.8160515
974	TTI2	Betweenness: 9.801534
975	MSTO1	Betweenness: 9.704628
976	DMPK	Betweenness: 9.150653
977	MYLK2	Betweenness: 8.976293
978	TEKT4	Betweenness: 8.723654
979	THAP4	Betweenness: 8.55725
980	FRK	Betweenness: 8.466813
981	PRKY	Betweenness: 8.432375
982	NOS1	Betweenness: 8.422812
983	GBA	Betweenness: 8.104015
984	LIMK2	Betweenness: 7.878439
985	VPS41	Betweenness: 7.834812
986	CKS2	Betweenness: 7.652091
987	RCBTB2	Betweenness: 7.378415
988	GUCY1B3	Betweenness: 7.2839613
989	MYLK3	Betweenness: 7.2290916
990	PNRC1	Betweenness: 7.1238966
991	ADA	Betweenness: 7.0627294
992	ASPRV1	Betweenness: 6.827746
993	CAMKMT	Betweenness: 6.680181
994	RHOBTB2	Betweenness: 6.6391215
995	STARD9	Betweenness: 6.5584984
996	RAPSN	Betweenness: 6.429835
997	FBXO38	Betweenness: 6.0841293
998	C22orf29	Betweenness: 6.046808
999	KDM5A	Betweenness: 5.9945583
1000	MECR	Betweenness: 5.9238076
1001	METTL18	Betweenness: 5.919988
1002	CASP12	Betweenness: 5.76327
1003	PDRG1	Betweenness: 5.725828
1004	TUBAL3	Betweenness: 5.5318866
1005	WTIP	Betweenness: 5.490431
1006	ZNF423	Betweenness: 5.3930554
1007	DDR2	Betweenness: 5.3084526
1008	TSPYL2	Betweenness: 5.2942395
1009	PHF3	Betweenness: 5.157961
1010	ZNF74	Betweenness: 5.0962143
1011	KLHL29	Betweenness: 5.022007
1012	TRIM56	Betweenness: 4.9346514
1013	MYLK4	Betweenness: 4.9299917
1014	STK32C	Betweenness: 4.765385
1015	ZBTB49	Betweenness: 4.6133895
1016	BRSK1	Betweenness: 4.2973027
1017	PI4K2B	Betweenness: 4.2924395
1018	CD3G	Betweenness: 4.263198
1019	DYRK4	Betweenness: 4.2148237
1020	DAD1	Betweenness: 4.100561
1021	MBOAT1	Betweenness: 3.874322
1022	PIWIL1	Betweenness: 3.825979
1023	YTHDC2	Betweenness: 3.80713
1024	STK36	Betweenness: 3.7855797

1025	NHLRC1	Betweenness: 3.7225924
1026	SH3RF2	Betweenness: 3.6839066
1027	NUAK2	Betweenness: 3.6400256
1028	KLHL1	Betweenness: 3.6048656
1029	KLHL15	Betweenness: 3.5806797
1030	SLC12A3	Betweenness: 3.5232325
1031	NAIP	Betweenness: 3.341256
1032	HR	Betweenness: 3.2565336
1033	KLHL36	Betweenness: 3.2244575
1034	ONECUT1	Betweenness: 3.1971254
1035	KRT85	Betweenness: 3.1920443
1036	BIRC8	Betweenness: 3.0718503
1037	IP6K2	Betweenness: 2.9986665
1038	TSSK6	Betweenness: 2.9902303
1039	CSF1R	Betweenness: 2.9132645
1040	CDKL2	Betweenness: 2.833938
1041	KLHL22	Betweenness: 2.8224425
1042	CERK	Betweenness: 2.7025104
1043	METTL22	Betweenness: 2.591892
1044	HOXB1	Betweenness: 2.5697334
1045	PRR14L	Betweenness: 2.5459504
1046	EPHB6	Betweenness: 2.5453186
1047	FBXO4	Betweenness: 2.511877
1048	BMF	Betweenness: 2.5061526
1049	NOX5	Betweenness: 2.408999
1050	NLRP4	Betweenness: 2.3766704
1051	TRIM49	Betweenness: 2.3221035
1052	FBXO34	Betweenness: 2.321669
1053	HACE1	Betweenness: 2.2845418
1054	KLHL32	Betweenness: 2.0543785
1055	MZB1	Betweenness: 2.00886
1056	FLT4	Betweenness: 1.999292
1057	NEK11	Betweenness: 1.962155
1058	DOCK2	Betweenness: 1.9044006
1059	MUSK	Betweenness: 1.8087037
1060	CERS2	Betweenness: 1.5724645
1061	MS4A2	Betweenness: 1.431748
1062	FAM162A	Betweenness: 1.3442044
1063	[illegible]HOSPHO2-KLHL[illegible]	Betweenness: 1.3055259
1064	KLHL23	Betweenness: 1.3055259
1065	IFIT1	Betweenness: 1.285414
1066	GUCY1A2	Betweenness: 1.2627337
1067	STK33	Betweenness: 1.1648574
1068	ZMYM1	Betweenness: 1.1285586
1069	KRT35	Betweenness: 1.0991702
1070	FBXO27	Betweenness: 1.0698938
1071	TMEM54	Betweenness: 1.0608778
1072	CDKL4	Betweenness: 1.0513469
1073	CDC14A	Betweenness: 1.0394175
1074	ASB4	Betweenness: 1.0342323
1075	MKKS	Betweenness: 0.94223875
1076	MMP2	Betweenness: 0.9223496
1077	DTX4	Betweenness: 0.8618964
1078	UNC45B	Betweenness: 0.84689814

1079	RCBTB1	Betweenness: 0.8428422
1080	TESK1	Betweenness: 0.70378596
1081	TESK2	Betweenness: 0.70378596
1082	TAOK3	Betweenness: 0.67805326
1083	AMHR2	Betweenness: 0.6645474
1084	TSSK3	Betweenness: 0.6107384
1085	FBXL14	Betweenness: 0.5746685
1086	PSKH2	Betweenness: 0.4637709
1087	ROR2	Betweenness: 0.4583132
1088	STRADA	Betweenness: 0.43926278
1089	FBXO9	Betweenness: 0.3770287
1090	MAP4K2	Betweenness: 0.31822512
1091	PTPRN2	Betweenness: 0.3163481
1092	EIF2AK1	Betweenness: 0.29117364
1093	KLHL34	Betweenness: 0.28466168
1094	KLHL6	Betweenness: 0.28466168
1095	KBTBD4	Betweenness: 0.24108693
1096	CCDC117	Betweenness: 0.23470594
1097	ACVR2B	Betweenness: 0.19158201
1098	KCNQ4	Betweenness: 0.19120926
1099	GTF2IRD2B	Betweenness: 0.13675214
1100	GTF2IRD2	Betweenness: 0.13675214
1101	KLHL10	Betweenness: 0.08058194
1102	KLHL14	Betweenness: 0.08058194
1103	ZBED4	Betweenness: 0.08058194
1104	KLHL25	Betweenness: 0.08058194
1105	CAMKV	Betweenness: 0.06869726
1106	RHOBTB1	Betweenness: 0.06764841
1107	ARMC5	Betweenness: 0.066362716
1108	TYMP	Betweenness: 0.057142857
1109	POGK	Betweenness: 0.049829334
1110	TSSK2	Betweenness: 0.049829334
1111	TBX22	Betweenness: 0.049829334
1112	RAB40A	Betweenness: 0.048780486
1113	STYK1	Betweenness: 0.04347826
1114	FBXO10	Betweenness: 0.018867925
1115	ALPK1	Betweenness: 0.0
1116	SERPINB7	Betweenness: 0.0
1117	HRK	Betweenness: 0.0
1118	INSRR	Betweenness: 0.0
1119	KCNA6	Betweenness: 0.0
1120	KCNG1	Betweenness: 0.0
1121	DCLK2	Betweenness: 0.0
1122	SIM1	Betweenness: 0.0
1123	KCNS3	Betweenness: 0.0
1124	ZC3H7B	Betweenness: 0.0
1125	SLC34A1	Betweenness: 0.0
1126	CYP1A2	Betweenness: 0.0
1127	TNNI3K	Betweenness: 0.0
1128	FPGT-TNNI3K	Betweenness: 0.0
1129	C20orf194	Betweenness: 0.0
1130	DLX6	Betweenness: 0.0
1131	RPS6KL1	Betweenness: 0.0
1132	NRIP2	Betweenness: 0.0

1133	CAMK1G	Betweenness: 0.0
1134	MAP3K9	Betweenness: 0.0
1135	MELK	Betweenness: 0.0
1136	TIE1	Betweenness: 0.0
1137	IRX4	Betweenness: 0.0
1138	EPHA1	Betweenness: 0.0
1139	AIPL1	Betweenness: 0.0
1140	ACVR1C	Betweenness: 0.0
1141	AHSA2	Betweenness: 0.0
1142	GRK7	Betweenness: 0.0
1143	ZNF215	Betweenness: 0.0
1144	GRK4	Betweenness: 0.0
1145	FBXO40	Betweenness: 0.0
1146	MYO3B	Betweenness: 0.0
1147	FBXL13	Betweenness: 0.0
1148	PRKG2	Betweenness: 0.0
1149	PIWIL2	Betweenness: 0.0
1150	PSKH1	Betweenness: 0.0
1151	BOK	Betweenness: 0.0
1152	HIF3A	Betweenness: 0.0
1153	NPRL2	Betweenness: 0.0
1154	TRIM73	Betweenness: 0.0
1155	RGS11	Betweenness: 0.0
1156	ISX	Betweenness: 0.0
1157	KCTD8	Betweenness: 0.0
1158	9-Mar	Betweenness: 0.0
1159	PDIK1L	Betweenness: 0.0
1160	FBXL8	Betweenness: 0.0
1161	BBC3	Betweenness: 0.0
1162	STK32B	Betweenness: 0.0
