# Supplementary material for: Pharmacological Mechanisms Underlying the Therapeutic Effects of Danhong Injection on Cerebral Ischemia
Source: Evid Based Complement Alternat Med. 2021 May 21;2021:5584809. doi: 10.1155/2021/5584809 (PMC8163534; doi:10.1155/2021/5584809)
Supplement: Supplementary Materials — Table S1: the 37 candidate compounds of Danhong injection. Table S2: the 371 putative target proteins for the compounds. Table S3: the 413 IS-associated Homo sapiens target proteins from CTD with an inference score of ≥50. Table S4: the 61 IS-associated target proteins of Homo sapiens from Genecards with an inference score of ≥30. Table S5: degree centrality of nodes in PPI network. Table S6: betweenness centrality of nodes in the PPI network. Table S7: the GO functional enrichment analysis of diterpenoid quinones. Table S8: the KEGG pathway enrichment of diterpenoid quinones. Table S9: the KEGG pathway enrichment of DHI compounds. [file 5584809.f1.zip › 5584809.f1/S7 (1).pdf]

Table S7. The GO functional enrichment analysis of diterpenoid quinones

Expected value (E): Based on the number of genes in the inserted gene list (L) multiplied with the number of genes in the GO set (GO) and divided by the number of genes in the reference gene set (RG).
EnrichmentRatio (RE): If the observed value (O) exceeded the expected value (E), the RE = O/E.
Count: the number of genes enriched in one GO entry among all input genes.

Category	Set_ID	Description	Count	Expect	EnrichmentRatio	pValue	FDR	geneID
BP	GO:0071466	cellular response to xenobiotic stimulus	4	0.082093135	48.7251462	7.26E-07	0.006597952	CASP9;NR3C1;CYP1A2;PTGS1
	GO:0009410	response to xenobiotic stimulus	4	0.135381661	29.54609929	5.33E-06	0.024216341	CASP9;NR3C1;CYP1A2;PTGS1
	GO:0033993	response to lipid	4	0.431108977	9.278396437	4.92E-04	0.27126587	CASP9;NR3C1;RARA;CYP1A2
	GO:0014070	response to organic cyclic compound	4	0.434469515	9.206629834	5.07E-04	0.27126587	CASP9;NR3C1;RARA;CYP1A2
	GO:0009725	response to hormone	4	0.461833893	8.661122661	6.41E-04	0.298647864	CASP9;NR3C1;RARA;CYP1A2
	GO:0042493	response to drug	4	0.477196351	8.382293763	7.26E-04	0.314127138	CASP9;NR3C1;RARA;CYP1A2
	GO:1901700	response to oxygen-containing compound	4	0.74699952	5.354755784	0.003896356	0.526727584	CASP9;NR3C1;RARA;CYP1A2
	GO:0009719	response to endogenous stimulus	4	0.765722516	5.223824451	0.004268	0.562107184	CASP9;NR3C1;RARA;CYP1A2
	GO:0044093	positive regulation of molecular function	4	0.824291887	4.852649971	0.005590567	0.63529803	CASP9;CDC25B;RARA;HSP90AA1
	GO:0007049	cell cycle	4	0.834853577	4.791259344	0.005856205	0.642700058	NR3C1;CDC25B;RARA;HSP90AA1
	GO:0006915	apoptotic process	4	0.917426788	4.360020931	0.008242937	0.669076257	CASP9;NR3C1;RARA;MCL1
	GO:0032355	response to estradiol	3	0.060489678	49.5952381	2.30E-05	0.069674254	CASP9;RARA;CYP1A2
	GO:0071383	cellular response to steroid hormone stimulus	3	0.123379741	24.3151751	1.92E-04	0.27126587	CASP9;NR3C1;RARA
	GO:0046677	response to antibiotic	3	0.151704273	19.77531646	3.52E-04	0.27126587	CASP9;RARA;HSP90AA1
	GO:0032496	response to lipopolysaccharide	3	0.152184349	19.71293375	3.56E-04	0.27126587	CASP9;RARA;CYP1A2
	GO:0002237	response to molecule of bacterial origin	3	0.158425348	18.93636364	4.00E-04	0.27126587	CASP9;RARA;CYP1A2
	GO:0048545	response to steroid hormone	3	0.186269803	16.1056701	6.43E-04	0.298647864	CASP9;NR3C1;RARA
	GO:0071407	cellular response to organic cyclic compound	3	0.257321171	11.65858209	0.00164219	0.497638357	CASP9;NR3C1;RARA
	GO:0071396	cellular response to lipid	3	0.28468555	10.53794266	0.002196098	0.526727584	CASP9;NR3C1;RARA
	GO:0009617	response to bacterium	3	0.285645703	10.50252101	0.002217413	0.526727584	CASP9;RARA;CYP1A2
	GO:0080135	regulation of cellular response to stress	3	0.313970235	9.555045872	0.0029063	0.526727584	CASP9;MCL1;HSP90AA1
	GO:0032870	cellular response to hormone stimulus	3	0.330772924	9.069666183	0.003371856	0.526727584	CASP9;NR3C1;RARA
	GO:0051707	response to other organism	3	0.430628901	6.966555184	0.007098424	0.666055747	CASP9;RARA;CYP1A2
	GO:0043207	response to external biotic stimulus	3	0.431589054	6.95105673	0.007142752	0.666055747	CASP9;RARA;CYP1A2
	GO:0009607	response to biotic stimulus	3	0.444551128	6.74838013	0.007757874	0.666175959	CASP9;RARA;CYP1A2
	GO:1901701	cellular response to oxygen-containing compound	3	0.515602496	5.818435754	0.011699582	0.743568088	CASP9;NR3C1;RARA
	GO:0034097	response to cytokine	3	0.528084494	5.680909091	0.012494854	0.752256392	RARA;MCL1;HSP90AA1
	GO:0051726	regulation of cell cycle	3	0.530964954	5.650090416	0.012682854	0.758551482	CDC25B;RARA;HSP90AA1
	GO:0071495	cellular response to endogenous stimulus	3	0.646663466	4.639198218	0.021664557	0.936195849	CASP9;NR3C1;RARA
	GO:0080134	regulation of response to stress	3	0.653384542	4.591476855	0.022274165	0.936195849	CASP9;MCL1;HSP90AA1
	GO:0043085	positive regulation of catalytic activity	3	0.662506001	4.52826087	0.023117234	0.957966202	CASP9;CDC25B;HSP90AA1
	GO:0042981	regulation of apoptotic process	3	0.727796447	4.122031662	0.029685811	1	CASP9;RARA;MCL1
	GO:0043067	regulation of programmed cell death	3	0.734517523	4.084313725	0.030415565	1	CASP9;RARA;MCL1
	GO:0032270	positive regulation of cellular protein metabolic process	3	0.740758521	4.049902787	0.031102199	1	CASP9;CDC25B;HSP90AA1
	GO:0042127	regulation of cell proliferation	3	0.750840134	3.995524297	0.032229716	1	CDC25B;RARA;PTGS1
	GO:0051247	positive regulation of protein metabolic process	3	0.787325972	3.810365854	0.036500013	1	CASP9;CDC25B;HSP90AA1
	GO:0010941	regulation of cell death	3	0.791166587	3.791868932	0.036966842	1	CASP9;RARA;MCL1
	GO:0010557	positive regulation of macromolecule biosynthetic process	3	0.876620259	3.422234392	0.048208047	1	NR3C1;RARA;HSP90AA1
	GO:0045935	positive regulation of nucleobase-containing compound metabolic process	3	0.886701872	3.38332431	0.049641906	1	NR3C1;RARA;HSP90AA1
CC	GO:0043005	neuron projection	3	0.585687489	5.122185559	0.016580349	1	RARA;HSP90AA1;PTGS1
	GO:0044463	cell projection part	3	0.647984994	4.629736842	0.021785285	1	RARA;HSP90AA1;PTGS1
	GO:0120038	plasma membrane bounded cell projection part	3	0.647984994	4.629736842	0.021785285	1	RARA;HSP90AA1;PTGS1
	GO:0005739	mitochondrion	3	0.707099415	4.242684887	0.027503431	1	CASP9;NR3C1;MCL1
	GO:0097458	neuron part	3	0.768487467	3.903772189	0.03425996	1	RARA;HSP90AA1;PTGS1
MF	GO:0019901	protein kinase binding	5	0.302855772	16.50950872	3.90E-06	0.006575158	CASP9;NR3C1;CDC25B;RARA;HSP90AA1
	GO:0019900	kinase binding	5	0.3412527	14.65189873	7.01E-06	0.006575158	CASP9;NR3C1;CDC25B;RARA;HSP90AA1
	GO:0019904	protein domain specific binding	4	0.328293737	12.18421053	1.72E-04	0.08442158	CASP9;RARA;MCL1;HSP90AA1
	GO:0046914	transition metal ion binding	3	0.507799376	5.907844991	0.011218332	0.68530797	NR3C1;RARA;CYP1A2
	GO:0046983	protein dimerization activity	3	0.609551236	4.921653543	0.018474427	0.828840661	RARA;MCL1;HSP90AA1
	GO:0003723	RNA binding	3	0.76937845	3.899251404	0.034362249	1	NR3C1;RARA;HSP90AA1
