# Supplementary material for: Pharmacological Mechanisms Underlying the Therapeutic Effects of Danhong Injection on Cerebral Ischemia
Source: Evid Based Complement Alternat Med. 2021 May 21;2021:5584809. doi: 10.1155/2021/5584809 (PMC8163534; doi:10.1155/2021/5584809)
Supplement: Supplementary Materials — Table S1: the 37 candidate compounds of Danhong injection. Table S2: the 371 putative target proteins for the compounds. Table S3: the 413 IS-associated Homo sapiens target proteins from CTD with an inference score of ≥50. Table S4: the 61 IS-associated target proteins of Homo sapiens from Genecards with an inference score of ≥30. Table S5: degree centrality of nodes in PPI network. Table S6: betweenness centrality of nodes in the PPI network. Table S7: the GO functional enrichment analysis of diterpenoid quinones. Table S8: the KEGG pathway enrichment of diterpenoid quinones. Table S9: the KEGG pathway enrichment of DHI compounds. [file 5584809.f1.zip › 5584809.f1/S8 (1).pdf]

Table S8. The KEGG pathway enrichment of diterpenoid quinones

Expected value (E): Based on the number of genes in the inserted gene list (L) multiplied with the number of genes in the GO set (GO) and divided by the number of genes in the reference gene set (RG).

EnrichmentRatio (RE): If the observed value (O) exceeded the expected value (E), the RE = O/E .

Count: the number of genes enriched in one GO entry among all input genes.

Set_ID	Description	Cound	Expect	EnrichmentRatio	pValue	FDR	geneID
hsa04151	PI3K-Akt signaling pathway	3	0.389492504	7.702330508	0.005335667	0.369677036	CASP9;MCL1;HSP90AA1
hsa05200	Pathways in cancer	3	0.576536928	5.203482824	0.015849202	0.574093319	CASP9;RARA;HSP90AA1
