# Supplementary material for: Pharmacological Mechanisms Underlying the Therapeutic Effects of Danhong Injection on Cerebral Ischemia
Source: Evid Based Complement Alternat Med. 2021 May 21;2021:5584809. doi: 10.1155/2021/5584809 (PMC8163534; doi:10.1155/2021/5584809)
Supplement: Supplementary Materials — Table S1: the 37 candidate compounds of Danhong injection. Table S2: the 371 putative target proteins for the compounds. Table S3: the 413 IS-associated Homo sapiens target proteins from CTD with an inference score of ≥50. Table S4: the 61 IS-associated target proteins of Homo sapiens from Genecards with an inference score of ≥30. Table S5: degree centrality of nodes in PPI network. Table S6: betweenness centrality of nodes in the PPI network. Table S7: the GO functional enrichment analysis of diterpenoid quinones. Table S8: the KEGG pathway enrichment of diterpenoid quinones. Table S9: the KEGG pathway enrichment of DHI compounds. [file 5584809.f1.zip › 5584809.f1/S9 (1).pdf]

Table S9. The KEGG pathway enrichment of DHI compounds

Expected value (E): Based on the number of genes in the inserted gene list (L) multiplied with the number of genes in the GO set (GO) and divided by the number of genes in the reference gene set (RG).

EnrichmentRatio (RE): If the observed value (O) exceeded the expected value (E), the RE = O/E .

Set_ID	Description	Count	Expect	EnrichmentRatio	pValue	FDR	GeneID
hsa00982	Drug metabolism	4	0.128730574	31.07264957	5.91E-06	0.001926182	MAOB;MAOA;CYP1A2;CYP2C19
hsa04726	Serotonergic synapse	4	0.205611333	19.4541806	3.80E-05	0.004614926	MAOB;MAOA;PTGS1;CYP2C19
hsa04151	PI3K-Akt signaling pathway	4	0.63292532	6.31986093	0.002781846	0.06477726	CASP9;MCL1;HSP90AA1;RELA
hsa05200	Pathways in cancer	4	0.936872507	4.269524369	0.01128723	0.150199578	CASP9;RARA;HSP90AA1;RELA
hsa00380	Tryptophan metabolism	3	0.071516985	41.94807692	4.25E-05	0.004614926	MAOB;MAOA;CYP1A2
hsa05030	Cocaine addiction	3	0.087608307	34.2433281	7.85E-05	0.006394562	MAOB;MAOA;RELA
hsa05215	Prostate cancer	3	0.173428689	17.29817605	5.97E-04	0.032436975	CASP9;HSP90AA1;RELA
hsa04659	Th17 cell differentiation	3	0.191307936	15.68152408	7.96E-04	0.032436975	RARA;HSP90AA1;RELA
hsa04210	Apoptosis	3	0.24315775	12.33766968	0.001595686	0.052019364	CASP9;MCL1;RELA
hsa05206	MicroRNAs in cancer	3	0.268188695	11.18615385	0.00211445	0.055553906	CDC25B;MCL1;ABCB1
